# A continental-scale survey of *Wolbachia* infections in blue butterflies reveals evidence of interspecific transfer and invasion dynamics

**DOI:** 10.1093/g3journal/jkac213

**Published:** 2022-08-17

**Authors:** Vivaswat Shastry, Katherine L Bell, C Alex Buerkle, James A Fordyce, Matthew L Forister, Zachariah Gompert, Sarah L Lebeis, Lauren K Lucas, Zach H Marion, Chris C Nice

**Affiliations:** Committee on Genetics, Genomics and Systems Biology, University of Chicago, Chicago, IL 60637, USA; Department of Biology, University of Nevada, Reno, NV 89557, USA; Department of Botany, University of Wyoming, Laramie, WY 82071, USA; Department of Ecology & Evolutionary Biology, University of Tennessee, Knoxville, TN 37996, USA; Department of Biology, University of Nevada, Reno, NV 89557, USA; Department of Biology, Utah State University, Logan, UT 84322, USA; Department of Microbiology & Molecular Genetics, Michigan State University, East Lansing, MI 48824, USA; Department of Biology, Utah State University, Logan, UT 84322, USA; Bio-Protection Research Centre, School of Biological Sciences, University of Canterbury, Christchurch, New Zealand; Department of Biology, Population and Conservation Biology, Texas State University, San Marcos, TX 78666, USA

**Keywords:** *Wolbachia*, *Lycaeides*, infection acquisition, geography of infection, GBS data, host parasite interactions

## Abstract

Infections by maternally inherited bacterial endosymbionts, especially *Wolbachia*, are common in insects and other invertebrates but infection dynamics across species ranges are largely under studied. Specifically, we lack a broad understanding of the origin of *Wolbachia* infections in novel hosts, and the historical and geographical dynamics of infections that are critical for identifying the factors governing their spread. We used Genotype-by-Sequencing data from previous population genomics studies for range-wide surveys of *Wolbachia* presence and genetic diversity in North American butterflies of the genus *Lycaeides*. As few as one sequence read identified by assembly to a *Wolbachia* reference genome provided high accuracy in detecting infections in host butterflies as determined by confirmatory PCR tests, and maximum accuracy was achieved with a threshold of only 5 sequence reads per host individual. Using this threshold, we detected *Wolbachia* in all but 2 of the 107 sampling localities spanning the continent, with infection frequencies within populations ranging from 0% to 100% of individuals, but with most localities having high infection frequencies (mean = 91% infection rate). Three major lineages of *Wolbachia* were identified as separate strains that appear to represent 3 separate invasions of *Lycaeides* butterflies by *Wolbachia*. Overall, we found extensive evidence for acquisition of *Wolbachia* through interspecific transfer between host lineages. Strain *wLyc*C was confined to a single butterfly taxon, hybrid lineages derived from it, and closely adjacent populations in other taxa. While the other 2 strains were detected throughout the rest of the continent, strain *wLyc*B almost always co-occurred with *wLyc*A. Our demographic modeling suggests *wLyc*B is a recent invasion. Within strain *wLyc*A, the 2 most frequent haplotypes are confined almost exclusively to separate butterfly taxa with haplotype A1 observed largely in *Lycaeides melissa* and haplotype A2 observed most often in *Lycaeides idas* localities, consistent with either cladogenic mode of infection acquisition from a common ancestor or by hybridization and accompanying mutation. More than 1 major *Wolbachia* strain was observed in 15 localities. These results demonstrate the utility of using resequencing data from hosts to quantify *Wolbachia* genetic variation and infection frequency and provide evidence of multiple colonizations of novel hosts through hybridization between butterfly lineages and complex dynamics between *Wolbachia* strains.

## Introduction

The endosymbiotic bacteria in the genus *Wolbachia* (Hertig and Wolbach 1924; Hertig 1936) have been studied for their phenotypic effects on their invertebrate hosts, mostly arthropods and nematodes (Yen and Barr 1971; Charlat *et al.* 2003; Moran *et al.* 2008; Werren *et al.* 2008; Kriesner *et al.* 2013). The impacts on hosts include extraordinary reproductive manipulation as well as mutualistic interactions (Werren *et al.* 2008). Reproductive manipulations include cytoplasmic incompatibility (mortality of host embryos when infected males mate with uninfected females), feminization, sex ratio distortion, and male killing. Mutualistic interactions are observed when *Wolbachia* infections protect hosts from viral attack (Hedges *et al.* 2008; Teixeira *et al.* 2008) or facilitate sequestration of vital nutrients (Brownlie *et al.* 2009; Hosokawa *et al.* 2010). These interactions have spurred development of evolutionary models to explain the persistence of infection within populations and the spread of infections across populations, lineages, and taxa (Turelli and Hoffmann 1991; Turelli 1994; Kriesner *et al.* 2013, 2016). The manipulation of host biology has even been harnessed to control pest insect populations, including insects that vector human diseases (e.g. Kambris *et al.* 2009; Hoffmann *et al.* 2011; Iturbe-Ormaetxe *et al.* 2011; Walker and Moreira 2011; Ross *et al.* 2019). However, despite nearly a half a century of research on the phenotypic effects of *Wolbachia* on their hosts, we have a relatively poor understanding of (1) how *Wolbachia* infects novel hosts and (2) the historical biogeography of infection dynamics within and among host lineages.

*Wolbachia* infections occur in more than half of insect species (Werren *et al.* 1995; Hilgenboecker *et al.* 2008; Weinert *et al.* 2015; Zug and Hammerstein 2015; Bailly-Bechet *et al.* 2017) and acquisition by novel hosts can occur in multiple ways. Cladogenic acquisition, also known as cospeciation or codivergence, occurs when infections are acquired from an ancestral lineage, resulting in sister taxa sharing common *Wolbachia* strains or genotypes (Cooper *et al.* 2019; Sanaei *et al.* 2021). Introgressive acquisition occurs through reproductive exchange between lineages (i.e. via hybrid formation) and constitute host shifts (Cooper *et al.* 2019; Sanaei *et al.* 2021). Alternatively, horizontal transfer might result from parasitoid (Heath *et al.* 1999; Stevens *et al.* 2001; Duron *et al.* 2010; Gehrer and Vorburger 2012; Gupta *et al.* 2021) or ectoparasite attack (Hoy and Jeyaprakash 2005; Le Clec’h *et al.* 2013; Gupta *et al.* 2021), or possibly through predation and other food sources (Huigens *et al.* 2000, 2004; Gerth *et al.* 2013). Evidence for cladogenic acquisition is sparse (Raychoudhury *et al.* 2009; Gerth *et al.* 2013; Turelli *et al.* 2018) and requires phylogenetic information from *Wolbachia* and hosts. Distinguishing between introgressive acquisition and the various pathways of horizontal transfer not involving reproductive interaction can be accomplished by comparisons of divergence times of *Wolbachia* and mitochondrial DNA (mtDNA) (Conner *et al.* 2017; Turelli *et al.* 2018).

Once acquired, the dynamics of *Wolbachia* prevalence in a population are presumably governed by the phenotypic effects on hosts, host immune responses to infections, coevolution, and the fidelity of *Wolbachia* transmission from host mother to offspring (Turelli 1994; Weeks *et al.* 2007; Jaenike 2009; Hoffmann 2020; Sanaei *et al.* 2021). For example, models of cytoplasmic incompatibility-inducing strains, where infected females experience fitness effects that are dependent on the frequency of infected males predict that prevalence within a host population can exhibit decreases in frequency when infections are rare, but rapidly increase when infection frequency is above an unstable threshold frequency. Such “bistable” infection dynamics could produce variation in prevalence among host populations that depend on initial infection frequencies where some populations have low to nonexistent infections, whereas others might be fixed, or nearly fixed, for the infection (Turelli and Hoffmann 1991; Barton and TurelLi 2011; Kriesner *et al.* 2013). Alternatively, strains with positive, frequency-independent fitness effects on their hosts are predicted to increase in frequency within populations and spread spatially (Barton and TurelLi 2011; Kriesner *et al.* 2013) much like alleles under positive selection. Range-wide surveys of infection prevalence across species ranges provide critical information for understanding the spread and maintenance of infections (Hague *et al.* 2022). Such data can also be used to estimate the patterns of strain distribution among host species to inform models of evolution and transmission of *Wolbachia* (Kriesner *et al.* 2013; Turelli *et al.* 2018; Cooper *et al.* 2019). Further, temporal sampling could also be incorporated to investigate the factors that govern the spatial spread of infections (Riegler *et al.* 2005; Kriesner *et al.* 2013).

Despite a robust theoretical foundation for *Wolbachia* infection acquisition by novel hosts and evolutionary dynamics, detailed range-wide surveys of *Wolbachia* prevalence in natural populations and species have been conducted in a limited number of hosts (e.g. Shoemaker *et al.* 2000; Shoemaker, Ahrens, *et al.* 2003; Shoemaker, Keller, *et al.* 2003; Narita *et al.* 2006; Baldo *et al.* 2008; Nunes *et al.* 2008; Schuler *et al.* 2016, 2018; Turelli *et al.* 2018; Hague *et al.* 2021; Walker *et al.* 2021) or ecological communities (Gupta *et al.* 2021). Historically, *Wolbachia* infections have been assayed using PCR-based methods targeting *Wolbachia* 16S rDNA genes or other *Wolbachia*-specific markers such as the Multilocus Strain Typing (MLST) loci (Turelli and Hoffmann 1995; Werren and Windsor 2000; Baldo *et al.* 2006, 2008). Presence of *Wolbachia* in a host individual is indicated by an amplified band on agarose gels and strain identification can be performed by sequencing the MLST loci (Baldo *et al.* 2006, 2008). However, PCR-based assays can be time-consuming, especially for surveys of large numbers of individual hosts, and are subject to false-positive errors from contamination of samples and false-negative errors from failed PCR reactions, among other problems. While various methods have been developed to minimize them (e.g. Turelli and Hoffmann 1995; Nice *et al.* 2009), these errors can present challenges for range-wide surveys of infections in hosts.

A promising and inexpensive approach for such surveys uses *Wolbachia* resequencing data from phylogeographic and population genomics studies of host species (Richardson *et al.* 2012; Signor 2017; Pascar and Chandler 2018; Hinojosa *et al.* 2020; Arif *et al.* 2021). Here, we pursue a bioinformatics approach similar to these previous studies and use a large population genomics data set from multiple species of *Lycaeides* butterflies to highlight the potential of this approach for estimating infection prevalence and to support inferences of modes of acquisition and histories of infections.

**Fig. 1.**
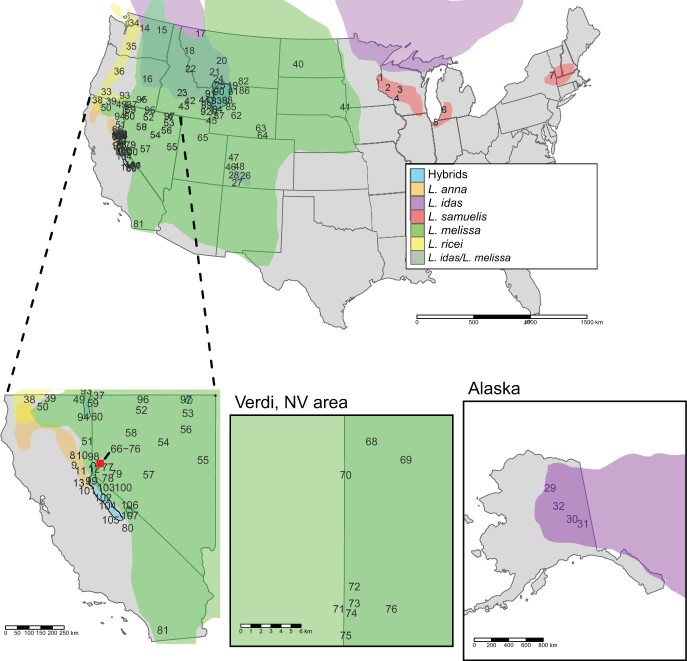
Range maps of the 5 nominal species of *Lycaeides* in the United States with the 107 sampled locations plotted as site numbers corresponding to Table 1. The dense sampling in the southwestern United States is expanded in the lower left. The inset square indicates the Verdi, Nevada sampling area, including sites 66–76, and is also expanded (bottom, middle). Sample locations in Alaska are illustrated in the map on the lower right.

*Lycaeides* butterflies colonized North America through Beringia approximately 2–4 MYA (Gompert, Fordyce, *et al.* 2008; Vila *et al.* 2011). This colonization was followed by a period of diversification that included extensive admixture among lineages (Nice *et al.* 2013; Gompert *et al.* 2014). We recognize 5 lineages that correspond to nominal species (Fig. 1 and Table 1; Gompert *et al.* 2014), including: *Lycaeides idas*, which also occurs in the Palearctic, and 4 North American endemics: *L. melissa*, *L. anna*, *L. ricei*, and *L. samuelis*. *Lycaeides samuelis* (formerly *L. melissa samuelis*) is known as the Karner blue butterfly and is a federally listed endangered species (Black and Vaughan 2005; Forister *et al.* 2011). We also recognize 3 distinct lineages within *L. melissa*: *L. melissa*—East, *L. melissa*—Rockies, and *L. melissa*—West (Chaturvedi *et al.* 2018). In addition to the nominal species, there are several admixed lineages that we refer to as hybrid lineages. These occur in several mountain ranges of the western United States. Putative ancient hybrid lineages formed from admixture between *L. melissa* and *L. anna* occur in the Sierra Nevada and in the White Mountains of California and Nevada (Gompert, Fordyce, *et al.* 2006; Nice *et al.* 2013; Gompert *et al.* 2014). In the vicinity of Jackson, Wyoming in the Grand Tetons, and Yellowstone area of the Rocky Mountains, we find populations that exhibit admixture between *L. melissa* and *L. idas* that we refer to as the Jackson hybrid lineage (Gompert *et al.* 2012, 2013, 2014; Chaturvedi *et al.* 2020). Hybrid lineages in the Warner Mountains of northeastern California, the Jarbidge Range in northern Nevada, and Steens Mountain in southeastern Oregon have complex ancestry potentially from *L. melissa*, *L. anna*, and *L. idas* (Gompert *et al.* 2014).

**Table 1.** Sample information for 107 *Lycaeides* butterfly collection localities.

No.	Locality	Nominal species	*n*	No. infected	Data	Wolbchia haplotype
1	Fish Lake	*L. samuelis*	20	20	Present	A1(14)
2	Eau Claire	*L. samuelis*	22	21	Present	A1(2)A8(1)
3	Black River	*L. samuelis*	17	17	Present	A1(14)
4	Fort McCoy	*L. samuelis*	23	23	Present	A1(20)A10(1)
5	Indiana Dunes	*L. samuelis*	21	1	Present	
6	Allegan	*L. samuelis*	30	0	Present	
7	Saratoga Spr.s	*L. samuelis*	27	0	Present	
8	Fall Cr	*L. anna*	20	20	G	C1(11)
9	Yuba Gap	*L. anna*	20	20	G	C1(14)J1(1)
10	Castle Pk	*L. anna*	18	16	G	C1(9)
11	Donner Pass	*L. anna*	18	17	G	C1(4)
12	Marlette Lk	*L. anna*	19	19	Present	C1(9)
13	Leek Spr.s	*L. anna*	20	20	G	C1(18)
14	Cottonwood	*L. idas*	25	25	Present	A2(24)
15	White Mt.	*L. idas*	24	24	Present	A2(15)
16	StrawB Mt.s	*L. idas*	20	20	G	A2(17)
17	Siyeh Cr	*L. idas*	20	20	G	A2(14)
18	Soldier Cr	*L. idas*	20	19	G	A2(12)
19	Tibbs Butte	*L. idas*	20	20	G	A2(17)
20	King’s Hill	*L. idas*	18	18	G	A2(12)
21	Garnet Pk	*L. idas*	20	19	G	A2(5)A12(1)B1(2)
22	Shook Mtn	*L. idas*	28	28	Present	A2(13)A13(4)A15(1)
23	Wolftone Rd	*L. idas*	4	4	Present	A2(3)A13(1)
24	Bunsen Pk	*L. idas*	20	19	G	A2(11)
25	Hayden V	*L. idas*	22	22	G	A2(11)B1(1)
26	Animas RH	*L. idas*	13	13	G	A1(6)A2(2)
27	Red Mt. P	*L. idas*	4	4	G	A1(1)A2(1)
28	Tomboy Rd	*L. idas*	24	24	G	A1(12)
29	Nolan Rd	*L. idas*	8	8	Present	
30	Spruce Barley	*L. idas*	20	20	Present	A2(1)B1(1)
31	Tok	*L. idas*	14	14	Present	A2(2)
32	Tolovana Cr	*L. idas*	9	9	Present	A2(1)A13(1)A15(1)
33	Soda Mt.	*L. ricei*	20	19	G	A2(12)
34	Rainy Pass	*L. ricei*	20	20	Present	A2(12)A17(3)
35	Chinook Pass	*L. ricei*	25	25	Present	A2(17)
36	Big Lk	*L. ricei*	20	20	G	A2(10)A3(1)A4(1)B1(5)
37	Cave Lk	*L. ricei*	24	24	G	A1(1)A2(20)
38	Marble Mts.	*L. ricei*	12	7	G	C1(5)G1(1)G2(1)
39	Shovel Cr	*L. ricei*	21	20	G	C1(15)C3(1)
40	Beulah	*L. melissa—*East	10	10	Present	A1(1)
41	Brandon	*L. melissa—*East	20	18	C	A1(3)
42	Silver Cr	*L. melissa—*East	6	6	Present	
43	Richfield	*L. melissa—*East	6	5	Present	A1(2)
44	Victor	*L. melissa—*East	20	20	G	A1(11)
45	Cokeville	*L. melissa—*East	10	10	G	A1(4)
46	Montrose	*L. melissa—*East	20	20	G	A1(9)A16(1)
47	De Beque	*L. melissa—*East	20	19	G	A1(5)
48	Cimarron	*L. melissa—*East	6	6	Present	A1(1)A7(1)
49	Goose Lk	*L. melissa—*East	20	20	G	A1(7)
50	Montague	*L. melissa—*East	19	19	G	A1(17)
51	Susanville	*L. melissa—*East	10	10	Present	A1(6)
52	Abel Cr	*L. melissa—*East	19	19	C	A1(1)
53	Deeth	*L. melissa—*East	20	20	G	A1(8)
54	Mill Cr	*L. melissa—*East	24	24	Present	A1(14)
55	East Cr CG	*L. melissa—*East	25	25	Present	A1(8)
56	Lamoille	*L. melissa—*East	20	19	G	A1(10)B1(2)
57	Ophir City	*L. melissa—*East	19	19	G	A1(8)
58	Star Cr	*L. melissa—*East	16	16	G	A1(6)
59	Upper Alkali	*L. melissa—*East	20	19	C	A1(6)A18(2)
60	Surprise V	*L. melissa—*East	20	20	G	A1(13)
61	Cody	*L. melissa—*Rockies	23	22	G	A1(11)A2(1)
62	Lander	*L. melissa—*Rockies	24	23	G	A1(4)
63	Wheatland	*L. melissa—*Rockies	16	16	Present	A1(9)A6(1)A19(2)I1(1)
64	Yellow Pine CG	*L. melissa—*Rockies	20	20	G	A1(9)A2(1)
65	Albion Meadow	*L. melissa—*Rockies	46	46	G	A2(40)
66	Lake Davis	*L. melissa—*West	4	4	Present	A1(2)
67	Sierravalley	*L. melissa—*West	20	20	Present	A1(2)
68	White Lk	*L. melissa—*West	27	27	Present	A1(15)A6(4)A11(1)A19(1)
						B8(1)B44(1)
69	Silver Lk	*L. melissa—*West	18	17	G	A1(5)B10(7)
70	Girl Farm	*L. melissa—*West	24	24	Present	A1(3)A6(1)A11(2)B5(1)
						B7(1)B10(1)B18(1)B23(2)
						B24(1)B25(1)B26(1)B27(1)
						B28(1)D2(1)E1(1)
71	Verdi Crystal	*L. melissa—*West	73	68	C	A1(14)A6(2)A11(1)A19(1)
						B5(2)B10(1)B23(1)B29(1)
						B30(2)B31(1)B32(1)B33(1)
						B34(1)H1(1)
72	Verdi classic	*L. melissa—*West	26	25	Present	A1(2)A19(1)B5(1)B28(1)
						B35(1)B36(1)B37(1)B38(1)
						B39(1)B40(1)
73	Verdi tracks	*L. melissa—*West	20	16	Present	B10(1)B11(1)B18(1)B22(1)
						B24(2)B33(1)B41(1)B42(1)
						B43(1)H2(1)
74	Verdi hwy	*L. melissa—*West	11	11	Present	A1(1)A19(2)B23(1)B37(1)
75	Qui	*L. melissa—*West	18	16	Present	A6(1)B2(1)B3(1)B4(2)
						B5(1)B6(1)B7(2)B8(1)
						B9(1)B10(1)B11(1)
76	Deer Mt Road	*L. melissa—*West	27	23	Present	B4(2)B7(1)B12(1)B13(1)
						B14(1)B15(1)B16(1)B17(1)
						B18(1)B19(1)B20(1)B21(1)
						B22(1)
77	Washoe Lk	*L. melissa—*West	20	18	G	A1(2)B10(1)
78	Gardnerville	*L. melissa—*West	18	17	G	B10(6)F1(1)
79	Red Earth	*L. melissa—*West	20	20	G	A1(8)
80	Bishop	*L. melissa—*West	20	20	G	A1(11)
81	Trout Pond	*L. melissa—*West	13	13	C	A1(4)
82	Big Ice	Hybrid	18	18	G	A2(11)
83	Blacktail Butte	Hybrid	46	45	G	A2(32)
84	Bull Cr	Hybrid	46	45	G	A2(27)
85	Dubois	Hybrid	41	41	G	A1(1)A2(29)
86	Hunt Mt.	Hybrid	30	30	G	A2(24)
87	Periodic Spr	Hybrid	20	20	G	A2(28)
88	Pinnacles Butte	Hybrid	20	19	G	A2(17)
89	Rendezvous Mt	Hybrid	32	32	G	A2(28)
90	Riddle Lk	Hybrid	30	28	G	A2(22)
91	Sheffield Cr	Hybrid	26	26	G	A2(22)
92	Swift Cr	Hybrid	4	3	G	A2(2)
93	Buck Mt	Hybrid	44	44	G	A2(28)A5(1)
94	Eagle Pk	Hybrid	40	40	G	A2(32)A9(1)
95	Steens Mt	Hybrid	13	11	G	A2(5)
96	Hinkley	Hybrid	26	26	Present	A2(21)A13(1)A14(2)
97	Jarbidge	Hybrid	42	40	Present	A2(30)A11(1)A13(5)A14(2)
						A15(1)
98	Mt Rose	Hybrid	52	8	G	
99	Carson Pass	Hybrid	50	32	G	C1(20)
100	Corey Pk	Hybrid	8	8	G	
101	Sonora Pass	Hybrid	44	33	G	C1(15)
102	Lake Emma	Hybrid	33	17	G	C1(8)
103	Sweetwater	Hybrid	23	13	G	C1(10)
104	Tioga Crest	Hybrid	38	21	G	C1(5)
105	South Fork	Hybrid	14	5	G	C1(5)
106	County Line	Hybrid	40	35	G	B1(1)B10(6)C1(18)D1(1)
107	Reed Flat	Hybrid	9	8	G	C1(4)C2(1)

Locality numbers, locality names, nominal species designations (see text for details), number of individuals sampled, number of infected individuals detected, data source and *Wolbachia* haplotypes (Fig. 4) are provided. Infected individuals were identified using a threshold of a minimum of 5 sequence reads of at least 80 bp in length. The data source column indicates previously published sequence data (G = Gompert *et al.* 2014, C = Chaturvedi *et al.* 2018 or sequence data presented here for the first time (present).

PCR-based surveys have demonstrated that North American *Lycaeides* harbor *Wolbachia* infections (Gompert, Forister, *et al.* 2008; Nice *et al.* 2009). For example, populations of *L. samuelis*, Karner blue butterflies, in the western portion of their range in Wisconsin were found to be nearly entirely infected (near 100% prevalence). These populations also possessed a mitochondrial haplotype identical to a haplotype found in *L. melissa* (the proposed source of the infection), but distinct from haplotypes found in the eastern portion of their range (east of Lake Michigan) (Nice *et al.* 2009). However, surveys of *Wolbachia* in *Lycaeides* have been limited in terms of geography and butterfly taxonomy. Here, we expand our survey to provide a continent-wide view of *Wolbachia* diversity using sequence reads from population genomics studies of *Lycaeides*.

The data considered here are Genotyping-by-Sequencing (GBS) sequence reads from 2,377 butterflies of the genus *Lycaeides* from 107 localities in North America sampled from 1996 to 2018 (Table 1 and Supplementary Table 1). These data were generated for several projects investigating patterns of differentiation and admixture across North America (Gompert *et al.* 2014), genomic changes during shifts to novel host plants (Gompert *et al.* 2015; Chaturvedi *et al.* 2018), and comparisons of genomic architecture between ancient hybrid lineages and a contemporary hybrid zone (Chaturvedi *et al.* 2020). A chromosome-level reference genome for *L. melissa* has been assembled to facilitate comparative genomic studies (Chaturvedi *et al.* 2018, 2020).

We used *Wolbachia* sequence reads found among GBS reads from North American *Lycaeides* butterflies to address the following questions: (1) how does variation in detection thresholds of sequence depth and sequence length influence *Wolbachia* infection frequency estimation? (2) how does the frequency of infection (prevalence) vary across host populations, lineages, and geography? and (3) how are *Wolbachia* genotypes and groups of genotypes (which we equate to strain types) distributed across geography and host taxonomy? We use the answers to these questions to construct hypotheses about the history and biogeography of infections in *Lycaeides*. We also discuss the opportunities and limitations to using *Wolbachia* reads present in resequencing data as an inexpensive tool for understanding *Wolbachia* dynamics in natural populations. We also argue that similar data from other host taxa might contribute to the growing understanding of the evolution and history of *Wolbachia*-host interactions.

## Materials and methods

### Sequencing of *Lycaeides* individuals

We extracted genomic DNA, generated GBS libraries, and sequenced these libraries following the methods described in Parchman *et al.* (2012), Gompert *et al.* (2014), and Chaturvedi *et al.* (2018). Briefly, genomic DNA was extracted from thoracic tissue for all specimens and purified using Qiagen’s DNeasy Blood and Tissue kit (Qiagen Inc.). Genomic DNA was digested with restriction enzymes EcoRI and MseI. Adapters, including a unique 8- to 10-bp sequence barcode and the Illumina primer sequences, were ligated to the fragmented DNA with T4 DNA ligase. Adaptor and primer sequences are provided in Supplementary Table 2. We then PCR-amplified the fragment libraries with standard Illumina PCR primers. Amplified libraries were then pooled and size selected (300–450 bp) with a BluePippin. The GBS libraries were sequenced across several lanes of Illumina HiSeq 2500 or HiSeq 4000 (100 bp, single-end reads) by the Genome Sequencing and Analysis Facility at the University of Texas (Austin, TX).

### Obtaining *Wolbachia* sequence reads from host GBS reads

Though we knew from PCR-based surveys that *Lycaeides* butterflies harbor *Wolbachia* (Gompert, Fordyce, *et al.* 2008; Nice *et al.* 2009), we had very little information about strain types or even the diversity of strains that might be encountered in a broader survey. Preliminary assemblies of sequence reads from *Lycaeides* to different publicly available *Wolbachia* genomes revealed variation in number of assembled reads across localities and reference genomes (data not presented). We interpreted this as a possible indication that butterflies from different localities, or taxa, harbored a diversity of *Wolbachia* strains and that different reference genomes might yield better assemblies for some strains and therefore also some host localities or taxa.

Given this possibility, we explored several assembly strategies for creating reference genomes in silico. Our first assembly was performed by concatenating 3 *Wolbachia* reference genomes representing each of supergroups A, B, and F (Ramírez-Puebla *et al.* 2015). Reference genomes are available for supergroups A–F. *Wolbachia* supergroups A and B are commonly found in insects. Supergroup F is found in both insects and nematodes, while, among supergroups with representative *Wolbachia* reference genomes, supergroups C, D, and E are found exclusively in nematodes (Ramírez-Puebla *et al.* 2015). Thus, this concatenated genome we constructed represented the most likely supergroups that might be observed in *Lycaeides* butterflies. The 3 representative reference genomes came from *Wolbachia* in *Drosophila melanogaster* (*wMel*, supergroup A; Wu *et al.* 2004), *Aedes albopictus* (*wAlbB*, supergroup B; Mavingui *et al.* 2012), and *Cimex lectularius* (*wCle*, supergroup F; Nikoh *et al.* 2014).

Because nuclear integration of *Wolbachia* genes into the host genome (e.g. Nikoh *et al.* 2008; Choi *et al.* 2015) is possible, we mapped putative *Wolbachia* reads identified by assembly to the concatenated reference genomes described above to the *L. melissa* reference genome (Chaturvedi *et al.* 2018) (details in Supplementary Table 3). Upon querying the location of the mapped *Wolbachia* reads in the host genome, we found that *all* the reads mapped to one of the smaller scaffolds (Scaffold 1260, 1.62 Mb) out of the 1,651 scaffolds in the *L. melissa* genome, and not from any of the larger chromosomal level (23 autosomes and Z sex chromosome) scaffolds. Based on the length (similar to size of other *Wolbachia* genomes) and mapping metrics of this region, we believe this scaffold to be the genome of *Wolbachia* infecting the host butterfly individual used in the genome assembly. We then pursued a second assembly in which we used this Scaffold 1260 as a species-specific reference *Wolbachia* genome for further analysis.

Lastly, we used a pan-genome approach (Tettelin *et al.* 2005; Vernikos *et al.* 2015) to build a reference representing the super set of genes from the above *Wolbachia* reference genomes. Here we used the supergroup A, B, and F genomes described above plus the Scaffold 1260 from the *Lycaeides* reference genome. The pan-genome was constructed by first annotating the representative *Wolbachia* genomes using prokka (version 1.14.6, Seemann 2014) to convert the fasta files to gff format (using the ‘Moderate’ parameters in https://github.com/tseemann/prokka, last accessed 2022 Jul 13), and then combining the files to produce a reference pan-genome using the Roary (version 3.13.0, Page *et al.* 2015) and GNU Parallel softwares (Tange 2011). Genes with paralogs and genes with less than 98% BLASTp percentage identity with each other were removed from the pan-genome. Finally, the pan-genome contained 11, 114, and 4,547 genes (total: 4,672 genes) present in 3, 2 and 1 of the 4 constituent genomes, respectively. The pan-genome was also considerably larger at approximately 3.25 Mb (as expected), compared to Scaffold 1260 (1.62 Mb) and the concatenated reference (2.8 Mb).

In all 3 iterations (i.e. using the concatenated reference, Scaffold 1260 from the *Lycaeides* reference genome, or the pan-genome), reads were aligned using bowtie2 software (all aligned reads reported using -a –al –no-unal, version 2.3.4.2, Langmead and Salzberg 2012). The mapped reads from each of the 2,377 individuals were output as sam files to allow for easy parsing and analysis downstream. Multiple previous studies (e.g. Richardson *et al.* 2012; Signor 2017; Scholz *et al.* 2020) show that an approach similar to the above is effective in not only retrieving large amounts of endosymbiont genomic data from host reads but also conducting population-level analyses on the extracted endosymbiont lineages. There were minor differences in the metrics of the intermediate bioinformatics analyses (e.g. number of reads, etc., listed in Supplementary Table 3) depending on the reference genome used, but we found very similar results in detecting infected individuals (see below) and in the final construction of the haplotype networks, identification of strains and in the geographic patterns of genetic variation (see below) across these assemblies. As a result, our subsequent analyses were based on the pan-genome reference assembly as this contains a super set of our genes from all assemblies. Details about the analyses with the other reference genomes are presented in the Supplementary Material (for instance, gene annotations for the pseudo-haplotype in the Scaffold 1260 reference presented in Supplementary Table 4).

### Detecting infection from the mapped *Wolbachia* reads

We first quantified the number of mapped reads and the length of mapped reads in an individual’s sam file from the pan-genome assembly as 2 metrics for detecting infected individuals. We then examined how various minimum thresholds of these 2 metrics affected the classification of host individuals as infected and compared the results to a previous PCR-based study of a subset of the current individuals (128 out of 2,377) from Nice *et al.* (2009). To collect these metrics, we used samtools view -F 2432 (skipping secondary alignments, version 1.12, Li *et al.* 2009) to determine the length (in base pairs, bp) and number of reads of each unique individual alignment for reads filtered to have a mapping quality of greater than 20 (less than a 1% chance of error, as is standard in typical pipelines). A similar type of bioinformatics approach has been previously used successfully by Pascar and Chandler (2018) to detect *Wolbachia* infection in various arthropod species. We deviate from previous purely bioinformatics studies by choosing a more appropriate threshold (for *Lycaeides*) for infection that maximizes concordance of infection status with results from the previous PCR-based amplification study in these same butterfly species.

### Quantifying genetic diversity in *Wolbachia* strains

The individual sam files were each compressed into bam files (using samtools) for more efficient downstream analyses. We then performed variant calling and genotyping on the sorted and indexed bam files from the previous step using the bcftools mpileup command (skipping indels) followed by the bcftools call and view commands (version 1.9, Li 2011) to produce a raw vcf file across all 2,377 individuals using the pangenome reference genome described above. We ignored indels, assumed a ploidal level of 1 (haploid) and retained only bi-alleleic sites (–ploidy 1–variants-only -m2 -M2 -v snps). The choice to employ a haploid model was based on preliminary analyses from a diploid model. Given the existence of more than 1 major *Wolbachia* strain and sympatry among strains in some instances (see Results), it is possible that individual host butterflies could contain multiple infections (i.e. a single individual hosting 2 or more *Wolbachia* strains). However, models for variant calling with higher ploidy (for instance, a diploid model that might be more appropriate for multiple infections) compromised our ability to call variants as haplotypes because phasing of alleles at multiple sites was not possible. Therefore, we employed the haploid model to produce useful haplotypic data. This undoubtedly prevented discovery of additional haplotypes in individuals with multiple infections, but did produce population genetic data for those individuals with single infections. The raw vcf file was then filtered to only keep sites with a maximum missingness of 25% using vcftools (version 0.1.14, Danecek *et al.* 2011). The final vcf file contained 115 SNPs and 2,377 individuals in total, as a result of our conservative filtering.

The alleles in each individual from this vcf file could now be regarded as representing *Wolbachia* haplotypes. However, to minimize uncertainty in the haplotypic data, we again filtered the data by retaining individuals with no missing data across variant sites (i.e. individuals with no missing data had at least 1 read of mapping quality greater than 20 of either the reference or the alternative allele at every site). We retained 1,277 individuals (out of 2,113 infected individuals, see Results) with haplotypes of length 115 bp.

We clustered the individual haplotypes using a statistical parsimony network approach (Templeton *et al.* 1992; Crandall *et al.* 2000) using the haplotypes (version 1.1.2, Aktas 2020) package in R with a parsimony threshold of 95%. All analyses in R were performed on version 4.0.3 (R Core Team 2021). As a complimentary approach, we performed a principal coordinates analysis (PCoA) on the matrix of pairwise sequence distances calculated with the haplotypes package and using the prcomp function in R. Based on these analyses, we identified 3 major groups of haplotypes that we consider as distinct *Wolbachia* strains (see Results). Strain types, and haplotypes within strains, were then mapped onto the geographical and taxonomic distributions of the host butterflies. Thus, the distribution of *Wolbachia* strains and haplotypes (choropleth maps produced using tmap v3.3-1, Tennekes 2018) were examined in the context of the biogeography of their hosts and used to construct hypotheses about the origin and dynamics of infection within *Lycaeides* butterflies.

### Reconstructing demographic history of *Wolbachia* strains

Lastly, we investigated the demographic history, specifically, changes in effective population size through time, for each of the 3 major strains (see Results) to understand *Wolbachia* population dynamics. We created a NEXUS file of all haplotypes from each of the 3 major strains (see *Results*) and used BEAST v2.6.3 to estimate Bayesian Skyline Plots (Drummond *et al.* 2005). This method fits a piece-wise linear function to the estimated population size as calculated from coalescent rates across the sequence. A single long chain, total of 75 million steps with a burn-in of 50 million steps, thinned every 50,000 steps for *wLyc*A and a total of 50 million steps with a burn-in of 10 million steps, thinned every 50,000 steps for *wLyc*B and *wLyc*C, due to the large number of individuals and parameters in *wLyc*A, was run. We ran a coalescent Bayesian skyline analysis with a HKY site model (Hasegawa *et al.* 1985) with a strict clock and a uniform prior on the clock rate. The full settings in the BEAUti files are presented in the Supplementary Material. Convergence to a posterior distribution was assessed based on visualizations of the trace plots and calculation of effective sample sizes (ESS) of the posterior distribution for each network using Tracer v1.7.1 (Rambaut *et al.* 2018), which was also used to obtain uncorrelated parameter estimates from the sampling distribution.

## Results

### Genotype-by-sequencing data for *Lycaeides* individuals

For the 2,377 *Lycaeides* individuals sequenced, a total of 3,727,714,988 sequence reads were generated (mean = 1,568,244 per individual, median = 1,363,955 per individual). From the *Wolbachia* mapping protocol described in the Materials and Methods section and on filtering for reads with mapping quality (MAPQ) greater than 20, we obtained approximately 8.75 million reads spread across all individuals, with a median of approximately 3,500 mapped reads per individual and more than 90% of the reads having lengths greater than 80 bp. The total *Wolbachia* reads comprise approximately 0.2% of all sequence reads. The distribution of mapped read lengths is shown in Supplementary Fig. 1.

### Detecting infection from the mapped *Wolbachia* reads

**Fig. 2.**
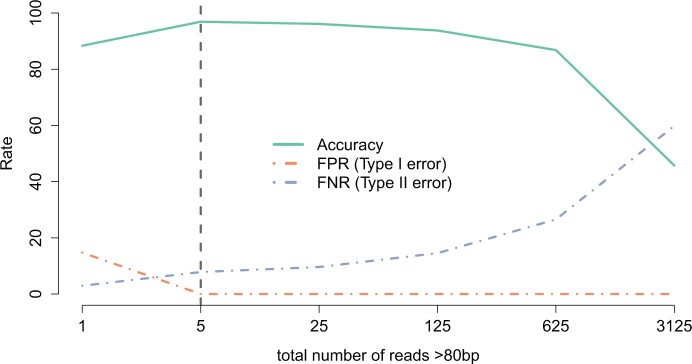
Accuracy and error rates of comparing bioinformatics results to previous PCR-based studies for detecting putative *Wolbachia* infections in the genome for 129 individuals (shown here for a threshold of varying number of reads of length greater than 80 bp). We used a threshold read depth of 5 for classifying an individual as infected, as it had the highest accuracy of 96.9% correspondence with the PCR-based results, while still maintaining a low FNR (classifying an individual as not being infected when the individual is inferred to be infected from PCR-based analysis). FPRs (compared to PCR-based results) were generally low. Note that the X-axis is on a log-scale.

We set our detection threshold for infection in individual butterflies at a minimum of 5 reads of >80 bp (with the maximal length being 87 bp). We found that results from this threshold matched very well with results from a previous PCR amplification study (Nice *et al.* 2009), with a 96.9% accuracy rate (i.e. concordance with PCR-based results, Fig. 2). A threshold read length of 80 bp was chosen since this was very close to the largest possible read from an individual, and would act as a stringent threshold for infection detection. We also found that >90% of mapped reads had lengths greater than 80 bp (see Supplementary Fig. 1). Similarly, we chose 5 reads as our threshold sequence depth because this threshold minimized error with comparison to PCR tests. We found that by increasing the threshold number of mapped reads, we were increasing our false negative rate (FNR) for classification by declaring putatively infected individuals (based on PCR tests) as being uninfected. This type of approach results in a sharp drop in the accuracy as we increase the threshold beyond a read depth of 600 since fewer individuals are classified as being infected (due to the stringent threshold) and therefore, increase the false negative error in our comparison. The 5 reads threshold provided a good balance between the false positive and false negative error rates (Fig. 2). However, we note that the PCR-based amplification studies are also prone to inaccuracies that could affect our accuracy estimates.

The numbers of infected individuals were not substantially changed by varying the minimum number of reads required to diagnose infected individuals for most localities. The exceptions where prevalence did vary with different thresholds were localities for Karner Blue butterflies in the eastern portion of their range (Indiana Dunes (5), Allegan (6), Saratoga (7)) and several of the Sierra Nevada hybrid lineage localities (98–99, 101–105). (Note: when referring to specific localities, we include the site number(s) from Table 1 and Fig. 1 in parentheses following the locality names.) In these localities, raising the minimum number of reads substantially reduced the number of infected individuals detected. Supplementary Table 5 presents the numbers of infected individuals using thresholds of a minimum of 1×, 5×, and 20× reads, and Supplementary Fig. 2 provides a detailed examination of the relationship between minimum number of reads and read lengths on the percentage of infected individuals detected across all individuals.

**Fig. 3.**
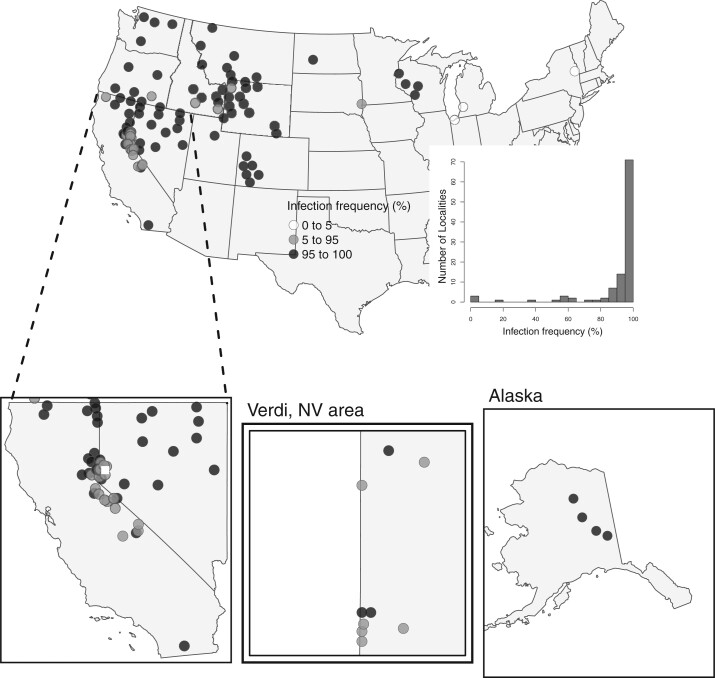
Bubble plots indicating the proportion of infected individuals in a population across the 107 sampled locations. Most populations in the western United States are mostly or wholly infected (>95%), while the *L. samuelis* populations in the east show low to no infection (<5%). Inset plots zoomed into regions of interest for visibility. The white square indicates the Verdi, NV sampling area, and is expanded (bottom, middle). Inset plot is a histogram of infection frequencies across 107 sampling localities using a threshold of a minimum of 5 sequence reads of at least 80 bp.

Based on the threshold of a minimum of 5 reads of at least 80 bp (Supplementary Fig. 2), we found that a majority of *Lycaeides* localities had infection frequencies that exceeded 90% of individuals, with 85 of the 107 sampled localities showing greater than 90% (with 64 localities having infection frequencies of 100%) (Fig. 3 and Table 1). In populations where we observed variation for infection (i.e. infection frequencies not 0 or 1), 90.6% of females and 86.4% of males were infected (population treated as a random effect, $\chi^{2}$ = 4.62, df = 1, *P*-value = 0.032). At the species or lineage level, most infections rates are greater than 94% (Table 2). The exceptions included *L. samuelis* localities in the eastern portion of their range (5–7) (infection rates: 0–0.5%), 1 population of *L. ricei* from the Marble Mts. in California (38) (infection rate: 58%), a small number of *L. melissa* populations, mostly in the western Great Basin (43, 73, 75, 76) (infection rates: 80–88%), 1 population of the hybrid lineages in the Jackson area (at Swift Creek (92) [infection rate: 75%]), in the Sierra Nevada (98, 99, 101–105) (infection rates: 15–75%) and in the White Mountains (106, 107) (infection rates: 87–89%) (Table 1).

**Table 2.** Infection frequencies and strain distributions for *Lycaeides* butterfly species or lineages.

Species/lineage	Locality	n	Infection rate	Dominant strain	Minor strains
*L. samuelis*	1–6	160	0.51	A1	
*L. anna*	7–13	115	0.97	C	
*L. idas*	14–32	333	0.99	A2	A1, B
*L. ricei*	33–39	142	0.95	A2	A1, C
*L. melissa*—East	40–60	350	0.98	A1	
*L. melissa*—Rockies	61–65	129	0.98	A1	A2
*L. melissa*—West	66–81	359	0.94	A1	B
Jackson Hybrid	82–92	313	0.98	A2	A1
Warner Hybrid	93–95	97	0.97	A2	
Sierra/Whites Hybrid	98–107	311	0.58	C	B

Locality numbers correspond with Fig. 1 and Table 1. Dominant strains are the most frequently observed major strains in each lineage (i.e. *wLyc*A, *wLyc*B or *wLyc*C). Minor strains are less frequently observed strains that are often dominant in other lineages and most likely occur in the focal lineage via interspecific transfer. The major subdivisions of strain *wLyc*A, A1 and A2 are treated as strains in this accounting.

### Quantifying genetic diversity in *Wolbachia* strains

**Fig. 4.**
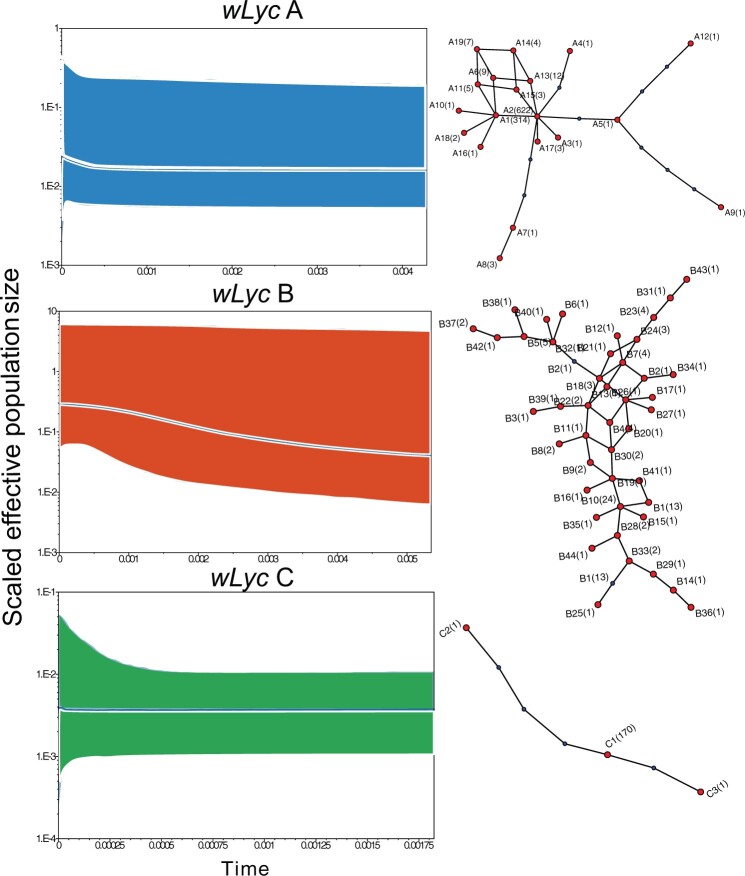
Demographic histories (left) and haplotype networks (right) for each major strain (*wLyc*A, *wLyc*B, *wLyc*C). Population sizes were estimated using BEAST 2 (Bouckaert *et al.* 2014). The median mutation-scaled effective population size (dashed line) and 95% credible interval (central posterior density, shaded region) for each strain is presented over time (measured in substitution rate). For simplicity, we assume equal substitution rates across strains to aid interpretation. Ninety-five percent parsimony networks show observed haplotypes in red and inferred haplotypes in blue with numbers of individuals observed possessing each haplotype in parentheses. Haplotypes are 115 bp in length.

**Fig. 5.**
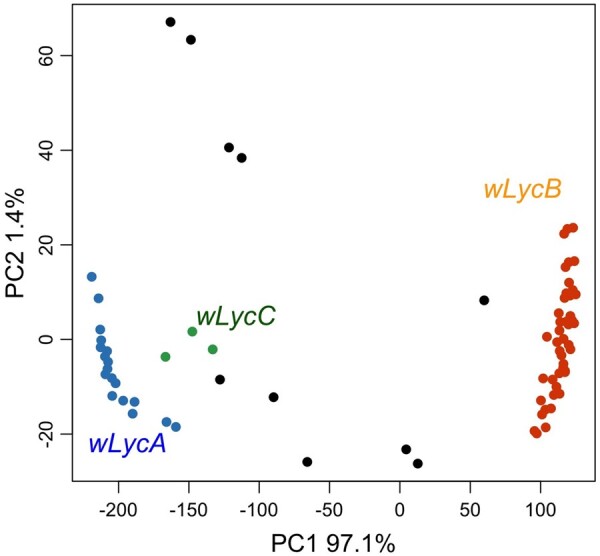
Plot of PCoA of *Wolbachia* haplotypes (76 in total) based on uncorrected pairwise distances among haplotypes. Colored dots represent 115 bp haplotypes in the 3 major strains (blue: *wLyc*A, orange: *wLyc*B, green: *wLyc*C). Strain *wLyc*A was found mostly in the *L. melissa*, *L. idas*, and *L. samuelis* populations continent-wide. Strain *wLyc*B was mostly found in the *L. melissa* populations in the western Great Basin. Strain wLycC was found exclusively in the *L. anna* populations and in the hybrids between *L. melissa* and *L. anna*. Black dots represent haplotypes found as singletons and not considered part of the 3 main strains (see Table 1 and Supplementary Fig. 3).

The filtered vcf file with 115 variable sites and 1,277 individuals was used for population genetic analyses. Based on a haplotype network analysis with 95% statistical parsimony and PCoA of pairwise distances among haplotypes, we found that 1,267 out of 1,277 genotyped individuals carried *Wolbachia* haplotypes from 1 of 3 major haplotype networks (Fig. 4 and Supplementary Fig. 3) that correspond to 3 clusters of haplotypes in our ordination of haplotypes (Fig. 5). We consider these networks as distinct *Wolbachia* strain types with individual haplotypes within networks representing mutational variation within strains (referred to hereafter as *wLyc*A, *wLyc*B, and *wLyc*C) (Table 1 and Supplementary Table 1). Each of these strains included between 3 and 44 distinct but closely related haplotypes (Fig. 4). The strains were substantially divergent from one another with mean pairwise divergences between strains ranging from 11.4% to 37.4% (Table 3). In addition, the diversity of *Wolbachia* strains and haplotypes within butterfly populations varied widely (Supplementary Table 1). Butterfly sampling localities ranged from localities that contained a single *Wolbachia* haplotype to localities with a maximum of 15 haplotypes (at Girl Farm (70)). The highest strain diversities were observed in the western Great Basin *L. melissa* populations and in some of the localities of hybrid lineages of *Lycaeides* (Table 1 and Supplementary Table 1 and Fig. 4).

**Table 3.** Sequence divergence (across 115 variable sites) within and among the 3 major strains presented as uncorrected percent sequence divergence (*p* × 100) and (standard deviations).

	*wLyc*A	*wLyc*B	*wLyc*C
*wLyc*A	3.3% (1.8%)		
*wLyc*B	37.4% (2.8%)	3.9% (1.7%)	
*wLyc*C	11.4% (2.2%)	32.1% (2.4%)	3.5% (1.7%)

*Wolbachia* strain *wLyc*A was observed in 992 individuals and was the most frequent strain. Among the 19 haplotypes within strain *wLyc*A, haplotypes A1 and A2 were observed in 936 individuals (94% of individuals with *wLyc*A haplotypes). Though these 2 haplotypes were differentiated by a single mutational step (Fig. 4), they were mostly observed in different butterfly taxa. The A1 haplotype was found almost exclusively in *L. melissa*, while A2 was limited to *L. idas* (Tables 1 and 2). The exceptions include all 3 disjunct *L. idas* localities sampled in Colorado (26–28) where A1 was observed; A1 was also observed in the 4 *L. samuelis* localities sampled in Wisconsin (1–4) (results that match earlier PCR-based surveys; Nice *et al.* 2009); A2 was observed in the *L. melissa* population at Albion Meadows (65), in the Jackson hybrids (82–92), and hybrid lineages in the Warner Mountains (93–94), Jarbidge Mountains (97), and at Steens Mountain (95) (notably not in the hybrid lineages in the Sierra Nevada and White Mountains in California and Nevada (98–107) for which *L. anna* is the maternal parent) (Table 2). Both A1 and A2 were also observed in the contemporary hybrid zone between *L. melissa* and *L. idas* at Dubois (85) (Chaturvedi *et al.* 2020), in the *L. ricei* population at Cave Lake (37), and in two *L. melissa* localities in the Rockies (61, 64) (Supplementary Fig. 5).

Strain *wLyc*B was observed in 103 individuals and included 44 haplotypes. Strain *wLyc*B haplotypes occurred most frequently in the populations of *L. melissa* in the western Great Basin (68–78) (“L. melissa—West” in Table 1 and Supplementary Table 1). Haplotypes B1 and B10 were the most common *wLyc*B haplotypes in these western populations. The other haplotypes occur in low frequency in these *L. melissa* West populations and at County Line (106), part of the hybrid lineage in the White Mountains, and in 2 *L. idas* populations (21, 25), 1 population of *L. melissa* East (56), the Big Lake (33) population of *L. ricei*, and 3 populations of *L. idas* (Spruce Barley (30), Garnet Peak (21), and Hayden Valley (25); Supplementary Fig. 6).

Strain *wLyc*C was observed in 172 individuals and included 3 haplotypes. Strain *wLyc*C haplotypes were confined to *L. anna* populations (8–13) and hybrids in the Sierra Nevada and White Mountains (99, 101–107). These hybrids have mixed ancestry from both *L. melissa* and *L. anna* and the latter is presumed to be the maternal lineage based on patterns of mtDNA variation (Gompert, Fordyce, *et al.* 2006). The 2 exceptions for the distribution of *wLyc*C haplotypes was their presence in the Shovel Creek, CA (39) and Marble Mt.s (38) *L. ricei* populations, which are the southern-most sampled *L. ricei* localities and adjacent to the range of *L. anna* (Supplementary Fig. 7).

For all 3 major strains, we found distinct right-skewed frequency distributions with 1–6 haplotypes observed in the majority of individuals and the remaining haplotypes were found in relatively few individuals, often spread over extensive areas (Fig. 4 and Table 1). The remaining 10 *Lycaeides* individuals that did not possess *Wolbachia* haplotypes from strains *wLyc*A, *wLyc*B, or *wLyc*C contained very rare haplotypes assigned to 7 rare strains (*wLyc*D-*wLyc*J) that were observed as singletons, 5 (D2, E1, F1, H1, and H2) in 4 localities in the western Great Basin (70, 71, 73, 78) (*L. melissa* West), 1 (D1) in the County Line (106) hybrid population, 2 (G1 and G2) in the Marble Mountains (38), 1 (I1) at Wheatland (*L. melissa* Rockies, 63), and 1 (J1) at Yuba Gap (*L. anna*, 9) (Table 1 and Supplementary Fig. 2).

### Reconstructing demographic history of *Wolbachia* strains

Based on our analysis of demographic history across the haplotypes within each of the 3 major strains, we find different patterns for each strain in the past (Fig. 4). We found well-mixed trace plots for all 3 strains and ESS values of about 200 for strain *wLyc*A and *wLyc*C, and about 400 for strain *wLyc*B (all 3 above the recommended threshold for independent samples from the BEAST2 manual). Strain *wLyc*A (which contains mostly the *L. melissa* and *L. idas* individuals) shows a constant scaled population size of 0.02 stretching into the very distant past. Strain *wLyc*B (which includes individuals from the western Great Basin (68–78), the hybrid lineages in the White Mountains in California (106), and Jackson, Wyoming area (83–84)) seems to have existed at much higher population sizes (∼2.5× population size of strain *wLyc*A) in the distant past, but has experienced a growth phase starting 0.003 time units in the past and has grown up to ∼4× its previous size since then. Strain *wLyc*C (which is observed in the *L. anna* individuals and adjacent localities) has a very small and constant population size (roughly 0.1× of the other 2 strains) stretching into the distant past. Based on the tree event times presented in Supplementary Fig. 8, we observe that both *wLyc*A and *wLyc*B strains have undergone population size changes in the recent past whereas strain *wLyc*C shows the highest spike at time 0, indicating that population size has been relatively constant over previous time periods. The time units are measured in substitutions and we assume equal rates across the strains to aid in interpretation.

## Discussion

We used a bioinformatics approach for detecting *Wolbachia* infection from GBS reads of 2,377 *Lycaeides* butterflies and validated the results from this approach by comparison with PCR-based analyses of a small subset of the host individuals (Nice *et al.* 2009). Using a threshold of a minimum of 5 reads of at least 80 bp, we found that most individuals were infected (2,117 out of 2,377 surveyed) and 105 out of 107 localities contained infected individuals. Infection prevalences within locality samples ranged from 0% to 100% of individuals infected with a mean infection prevalence per locality of 91% infected individuals. Population genetic analyses of *Wolbachia* haplotype data provided relatively detailed phylogeographic information on 3 major *Wolbachia* strains that infect *Lycaeides* butterflies in North America. Examination of the geographic and host-taxonomic distributions of *Wolbachia* strains revealed extensive sharing of strains between populations and lineages of *Lycaeides* which represents evidence for introgressive acquisition (Tables 1 and 2). Coalescent-based demographic inferences also provided evidence that 1 of the major strains has had a recent and dramatic increase in effective population size and might currently be invading and possibly displacing another strain.

Varying the threshold minimum sequence length had little effect on detecting infected individuals because the vast majority of sequence reads were greater than 80 bp in length (Supplementary Fig. 1). While the threshold of a minimum of 5 (5) reads provided the greatest accuracy (based on comparisons to PCR surveys), varying the minimum number of reads threshold had a limited impact on estimated infection frequencies except in 10 localities (5–7, 98–99, 101–105, see Supplementary Tables 1 and 5). In these localities, increasing the minimum reads threshold substantially reduced our estimate of prevalence of infected individuals. Three of these localities occur in the eastern portion of the range of the Karner Blue butterfly (*L. samuelis*) (5–7), but Karner blue populations in the western portion of the range (1–4) do not exhibit the same reduction in estimated prevalence with increasing minimum reads threshold. Similarly, the other localities that show the decline in numbers of infected individuals with increasing minimum reads threshold occur in the hybrid lineage of *Lycaeides* in the Sierra Nevada (98–99, 101–105), yet other hybrid lineages do not show a similar pattern. It is not immediately obvious why these localities differed in their apparent sensitivity to the minimum reads threshold. The overall number of sequence reads per individual could affect the probability of detection, but while the eastern Karner localities have lower median number of reads compared to the total set of 2,377 individuals, the Sierran hybrid populations have more reads per individual than the overall median (median number of sequence reads: eastern Karners: 1,078,622, Sierran hybrids: 1,810,680, overall: 1,359,589). Alternatively, it is possible that there is variation in *Wolbachia* densities within individuals among localities that influences detection probability (Unckless *et al.* 2009; Hague *et al.* 2022; Shropshire *et al.* 2021). While we cannot explain this observation at present, it suggests that variation in *Wolbachia* infection densities in host tissues might be an important consideration when mining resequencing data for evidence of endosymbiont infection. Variation among host taxa might require careful inspection of these thresholds. In the absence of corroborating PCR-based data on infection status, we recommend examining a range of thresholds to understand how these affect the probability of detection. It is also possible that more sophisticated statistical modeling that accounts for uncertainty created by variation in numbers of sequence reads, and possibly variation in *Wolbachia* densities, could improve the probability of detecting infections.

Population genetic analyses of *Wolbachia* infections in the *Lycaeides* system facilitated inference of infection history. We do not know where or how the 3 major *Wolbachia* strains (*wLyc*A, *wLyc*B, and *wLyc*C) were ultimately acquired by *Lycaeides* in North America. Analysis of *Wolbachia* infections from *Lycaeides* from Europe and Asia, or from associated parasites or parasitoids, might shed light on the origins of North American infections. However, our survey of geographic patterns of population genetic variation in combination with inference of demographic histories of the 3 major strains suggest that transmission of infection within North American *Lycaeides* butterflies occurred primarily through introgressive acquisition. We provide an overview of these patterns.

**Fig. 6.**
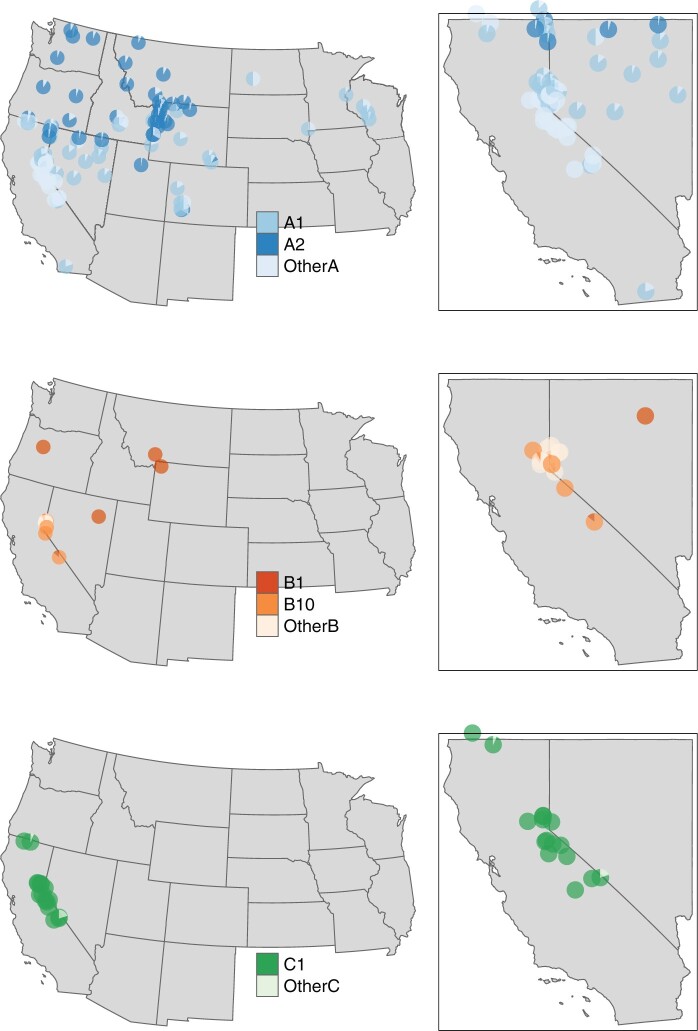
Pie charts showing the distribution of haplotypes from all 3 strains (row-wise: *wLyc*A, *wLyc*B, *wLyc*C). Haplotypes A1 and A2 are present in 90% of individuals infected with strain *wLyc*A. The label “OtherA” corresponds to rare haplotypes in *wLyc*A (A3–A19). Haplotypes B5, B9 and B10 make up 78% of all infections in the *wLyc*B strain. Haplotype C1 makes up for 98% of all infections in the *wLyc*C strain, and all other *wLyc*C haplotypes are found in localities that also include haplotype C1. Pies are only shown if a given haplotype is present in the population.

The comparison of demographic histories of each strain, as coalescent effective population sizes ($N_{e}\mu$), is facilitated by previous evidence for constant *Wolbachia* substitution rates over long timescales (Cooper *et al.* 2019). The demographic history of strain *wLyc*A reveals a relatively constant population size over time, and the geographic and taxonomic distribution of strain *wLyc*A haplotypes is possibly consistent with either a cladogenic mode or an introgressive mode of acquisition. The 2 most frequent haplotypes in *wLyc*A (A1 and A2) exhibit just 1 mutational difference (Fig. 4), yet A1 is largely confined to *L. melissa* individuals and A2 is found almost exclusively in *L. idas* individuals (Table 1 and Fig. 6). Exceptions to this pattern include hybrid lineages with either *L. melissa* or *L. idas* ancestry, or ancestry from both species (i.e. in the Jackson, Wyoming area (82–92), the contemporary hybrid zone between *L. melissa* and *L. idas* at Dubois (85), or localities at or near range boundaries, such as Cave Lake (37)). The confinement of these haplotypes largely within 2 *Lycaeides* species seems compatible with the hypothesis of cladogenic acquisition in the ancient past through a common ancestral lineage of *L. idas* and *L. melissa*, followed by independent divergence of the 2 lineages. Alternatively, the distribution of haplotypes A1 and A2 might be consistent with introgression from 1 of the species into the other accompanied by mutation. Further, the exceptions to the distributional pattern (e.g. hybrid lineages and a hybrid zone) appear to be examples of introgressive acquisition of strain *wLyc*A haplotypes outside of *L. melissa* and *L. idas*. Thus, there is perhaps more support for introgressive acquisition of *wLyc*A haplotypes, though cladogenetic acquisition cannot be ruled out. Evidence for multiple modes of *Wolbachia* transmission in natural populations is also found in the *Drosophila* (Cooper *et al.* 2019) and *Nasonia* (Raychoudhury *et al.* 2010) species complexes.

A similar demographic history of constant population size over time is seen in strain *wLyc*C, though the estimated population size of *wLyc*C is very much smaller than the other strains (Fig. 4). Strain *wLyc*C haplotypes are confined to *L. anna* populations and the hybrid lineages in the Sierra Nevada and White Mountains (98–107) for which *L. anna* is the presumed maternal lineage (Table 1 and Fig. 6) (Gompert, Fordyce, *et al.* 2006). The exceptions include 2 localities where strain *wLyc*C haplotypes were observed, both of which lie on the boundary between the ranges of *L. anna* and *L. ricei* at the Marble Mountains (38) and Shovel Creek (39). Thus, as with strain *wLyc*A haplotypes, *wLyc*C haplotypes appear to have spread to a limited extent outside of a *Lycaeides* species range via introgression among lineages, specifically in this case from *L. anna* to nearby populations of *L. ricei*.

The phylogeography of strain *wLyc*B is different compared to the other 2 strains. This is the least frequently observed strain over all and the majority of *wLyc*B haplotypes were observed in the western Great Basin in populations of *L. melissa* (68–78) (Table 1 and Fig. 6). In these locations, multiple *wLyc*B haplotypes are commonly observed along with *wLyc*A haplotypes. In fact, *wLyc*B haplotypes were observed without accompanying A haplotypes in only 3 locations (Verdi Tracks (73), Deer Mt. Road (76), and Gardnerville (78)). However, *wLyc*B haplotypes were observed in other widely distributed places and other *Lycaeides* taxa including: *L. idas* in Alaska (30) and Montana (21), *L. ricei* at Cave Lake (37), *L. melissa* in central Nevada (56), in the Jackson hybrid lineage (84), the hybrid lineage in the White Mountains of California (106), and the putative hybrid lineage at Hinkley in northern Nevada (96). The relative rarity of this strain, its recent population expansion (Fig. 4), coupled with its presence almost exclusively with *wLyc*A across different host species points to an introgressive mode of acquisition. Strain *wLyc*B haplotypes appear to be invading localities that already contain infections of strain *wLyc*A. Such a mode of acquisition will lead to the presence of multiple *Wolbachia* infections or haplotypes from different strains segregating in the same population and hence, an enriched genetic diversity of *Wolbachia* in these populations (Supplementary Fig. 4 and Table 1). The concentration of strain *wLyc*B haplotypes in *Lycaeides* localities in the western Great Basin, and the resulting high haplotype diversity there, suggests that this area is where the invasion of strain *wLyc*B began. There is weak evidence for the hypothesis that strain *wLyc*B is invading from 1 locality, Verdi Crystal (71), that was sampled over multiple years and strain *wLyc*B appears to have increased in frequency from 2011 to 2018 (Supplementary Table 6). The studies from which these GBS data were obtained were not designed to assay *Wolbachia* or for temporal comparisons, and we lack statistical power to fully test this hypothesis without further sampling. The host butterflies at these localities have colonized alfalfa (*Medicago sativa*) relatively recently (Forister, Philbin, *et al.* 2020; Forister, Yoon, *et al.* 2020), probably as 1 of 3 or more independent colonizations of alfalfa (Chaturvedi *et al.* 2018), and probably within the last 200 years (400–600 butterfly generations; Chaturvedi *et al.* 2018; Forister, Philbin, *et al.* 2020; Forister, Yoon, *et al.* 2020). So, it is possible that we are tracking the effect of host population expansion in the demographic history of strain *wLyc*B as it is impossible to disentangle the 2 histories without more information on host demography and quantification of *Wolbachia* titer levels. Thus, it seems that the invasion of a novel *Wolbachia* strain is occurring while the butterfly host is switching to a novel host plant. Whether there is any connection between these parallel host switches is an open question.

At a continental scale, the nominal species or lineages of *Lycaeides* butterflies each contained a dominant (most frequently observed) major strain (Table 2). Some lineages shared major strains. For example, *L. melissa* and *L. samuelis* shared strain *wLyc*A (specifically haplotype A1). This pattern is consistent with interspecific transfer from *L. melissa* to *L. samuelis* (Gompert, Nice, *et al.* 2006; Nice *et al.* 2009). Beyond their specific dominant strains, most lineages also contained other “minor” strains that were dominant in other lineages but observed at lower frequency in the focal lineage (Table 2). These minor strains were commonly observed at range margins and are consistent with limited interspecific transfer. Hybrid lineages were observed to be infected by major strains associated with their putative maternal parental lineage. The Sierra/Whites hybrid lineage is infected with *wLyc*C as is the inferred maternal parent *L. anna*. Similarly, the Jackson hybrid lineage is infected with *wLyc*A, specifically haplotype A2, as is its maternal parental lineage *L. idas* (Table 2). Taken together, these observations illustrate considerable interspecific transfer of *Wolbachia* strains among host lineages.

The distribution of *Wolbachia* strains in *Lycaeides* butterflies is paralleled by geographical patterns of mtDNA variation observed in previous studies of these butterflies (Nice *et al.* 2005; Gompert, Fordyce, *et al.* 2008; Gompert, Forister, *et al.* 2008). Because *Wolbachia* infections and mtDNA are maternally inherited, they are commonly observed to be in linkage disequilibrium (Turelli and Hoffmann 1991; Turelli *et al.* 1992; Jiggins 2003; Hurst and Jiggins 2005). Direct comparisons are not possible because even where sampling localities overlap with the current study, those older studies of mtDNA variation used different individuals that were not sequenced for this study. Nevertheless, the presence of 3 major *Wolbachia* strains discovered here parallels the 3 major mtDNA lineages discovered in *Lycaeides*. For example, using mitochondrial sequences of the cytochrome oxidase I (COI) and cytochrome oxidase II (COII) genes, Gompert, Forister, *et al.* (2008) found 3 mitochondrial lineages. One lineage (lineage III from Gompert, Forister, *et al.* [2008]) was widely distributed across space and butterfly taxonomy that corresponds to the distribution of *wLyc*A here. Another mtDNA lineage (lineage II) co-occurred with the first lineage and was detected in populations of *L. melissa* from the western Great Basin and from the hybrid population in the White Mt.s (County Line, 106), corresponding to the distribution of *wLyc*B. Lastly, the third mtDNA lineage (lineage I) was observed in *L. anna*, hybrid lineages derived from *L. anna* in the Sierra Nevada and adjacent *L. ricei* localities, corresponding to *wLyc*C. The close geographical correspondence of major *Wolbachia* lineages observed here and previous mtDNA haplotype distributions suggest that the expected disequilibrium between *Wolbachia* strains and mtDNA can be detected using GBS data.

Our survey of *Wolbachia* infection frequencies and genetic variation using GBS data from host *Lycaeides* butterflies suggests that this approach could be applied in other systems. Given the quantity of resequencing data generated recently, it might be possible to rapidly survey *Wolbachia* and other endosymbiont infections in a wide variety of host organisms and answer broad questions about the history, geography and mode of acquisition of infections. However, resequencing methods do not specifically target *Wolbachia* genomes and there exist several limitations. The sequence reads from *Lycaeides* GBS data did not map to any of the multilocus sequence typing (MLST) loci (Baldo *et al.* 2006, 2008) and it seems unlikely that GBS data in general will overlap the MLST loci. Thus, it will be impossible to identify conventionally designated strains (or possibly even *Wolbachia* supergroups) and connect studies phylogenetically from surveys of GBS data without further sequencing. Additionally, the stochasticity inherent in the methods for resequencing data, combined with the sparseness of endosymbiont sequence reads from host organisms, presents some challenges. Stochasticity arising from library preparation and from the sequencing of these multiplexed genomic libraries, among other possible sources of stochasticity, creates variation in sequence depth across fragments and individuals. This variation can contribute to false negatives for infection detection. Given variation in sequencing effort across studies, we note that the threshold for infection detection (here we used a minimum of 5 sequence reads) will need to be carefully examined for each investigation. False positives from GBS data seem less likely than false negatives compared to PCR-based methods for infection detection, though contamination of samples is an important consideration for both PCR-based and GBS survey methods. The usefulness of resequencing data for population genetics investigations of endosymbionts will be facilitated by the development of new methods for detecting infection and for genotyping that can, for example, more fully account for uncertainty and accommodate the possibility of multiple infections within individuals. Despite these limitations, the use of resequencing data can cheaply and relatively easily facilitate surveys of endosymbiont infection and population genetics.

## Supplementary Material

jkac213_Supplemental_Material

## Data Availability

DNA-sequence data have been deposited in the NCBI SRA with accession codes PRJNA246037, PRJNA577236, PRJNA432816, and PRJNA862870. Scripts for analysis are uploaded to https://github.com/VivaswatS/wolbachia_lycaeides.git. Supplemental material is available at *G3* online.
