# Supplementary material for: A continental-scale survey of Wolbachia infections in blue butterflies reveals evidence of interspecific transfer and invasion dynamics
Source: G3 (Bethesda). 2022 Aug 17;12(10):jkac213. doi: 10.1093/g3journal/jkac213 (PMC9526071; doi:10.1093/g3journal/jkac213)
Supplement: jkac213_Supplemental_Material [file jkac213_supplemental_material.pdf]

Supplemental Material

A continental-scale survey of *Wolbachia* infections in blue butterflies reveals evidence of interspecific transfer and invasion dynamics

Vivaswat Shastry[1], Katherine L. Bell[2], C. Alex Buerkle[3], James A. Fordyce[4], Matthew L. Forister[2], Zach Gompert[5], Sarah L. Lebeis[6], Lauren K. Lucas[5], Zachary H. Marion[7] and Chris C. Nice[8]

[1] Committee on Genetics, Genomics and Systems Biology, University of Chicago, Chicago, IL 60637, USA

[2] Department of Biology, University of Nevada, Reno, NV 89557, USA

[3] Department of Botany, University of Wyoming, Laramie, WY 82071, USA

[4] Department of Ecology & Evolutionary Biology, University of Tennessee, Knoxville, TN 37996, USA

[5] Department of Biology, Utah State University, Logan, UT 84322, USA

[6] Department of Microbiology & Molecular Genetics, Michigan State University, East Lansing, MI 48824, USA

[7] Bio-Protection Research Centre, School of Biological Sciences, University of Canterbury, Christchurch, New Zealand

[8] Department of Biology, Population and Conservation Biology, Texas State University, San Marcos, TX 78666, USA

Corresponding author: Vivaswat Shastry
Committee on Genetics, Genomics and Systems Biology
University of Chicago
Chicago, IL 60637, USA
vivaswat@uchicago.edu

Keywords: Wolbachia, Lycaeides, *infection acquisition, geography of infection, Genotyping-By-Sequencing*

Running title: *Wolbachia* in *Lycaeides*

Results from analyses using a concatenated genome as reference

Mapping *Lycaeides* GBS reads to the concatenated reference genome of supergroups A, B and F resulted in assembly of approximately 7.5 million reads. Variant calling (using the same criteria as listed in the main text) from this concatenated reference produced 116 variable sites. The functional annotation, obtained from the NCBI data base, for these can be found in Supplementary Table 4.

Results from analyses using Scaffold 1260 as reference

Mapping *Lycaeides* GBS reads to Scaffold 1260 from the *L. melissa* reference genome resulted in assembly of approximately 10 million reads. While this is considerably more reads assembled compared to using the concatenation of three *Wolbachia* genome sequences, variant calling from the reads assembled to Scaffold 1260 produced fewer variable sites (fewer SNPs). Variant calling from Scaffold 1260 produced 96 variable sites while variant calling from the concatenated *Wolbachia* genome sequences produced 116 variable sites. This disparity could arise because the assembly to Scaffold 1260 results in more rare sequences mapping to the reference, and thus more mapped reads overall, but few of the variable sites associated with these rare sequences pass the variant filtering criteria. Conversely, it is possible that the concatenated reference spans more of the strain variation across *Lycaeides* (i.e. beyond *L.*

melissa) resulting in more variable sites meeting filtering criteria.

Comparisons across assemblies

The distribution of read lengths were almost identical across the three assemblies with more than 80% of reads with lengths > 80bp for all three reference genomes. Correspondingly, we found that the number of infected individuals detected changed by < 0.07% across assemblies (16 individuals out of 2,377, see details below), when using the 5× threshold.

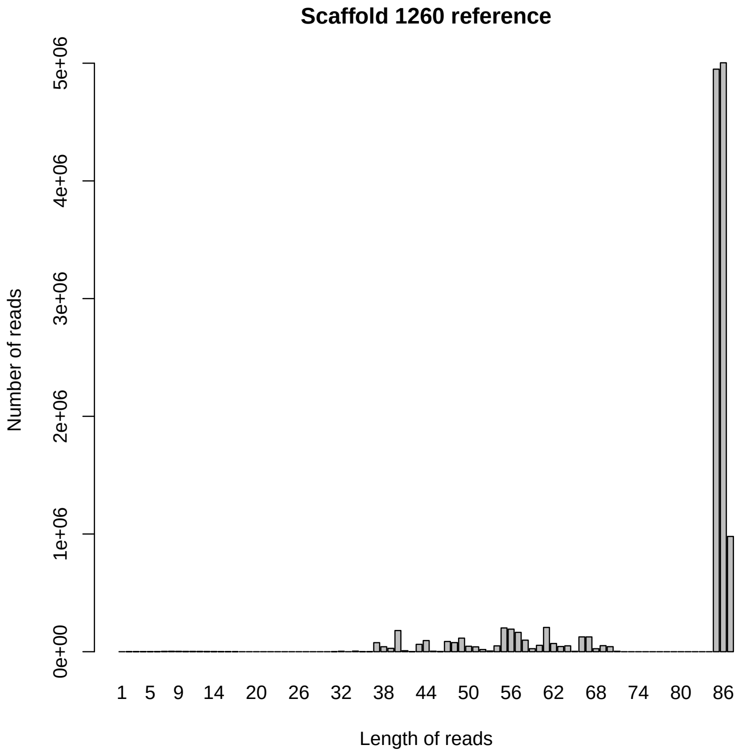

Supplementary Figure 1: Histogram of reads mapping to Scaffold 1260 reference of *Lycaeides melissa* for a total of 10 million reads. The histogram for the concatenated reference and for the pan-genome were very similar with peaks in the same locations.

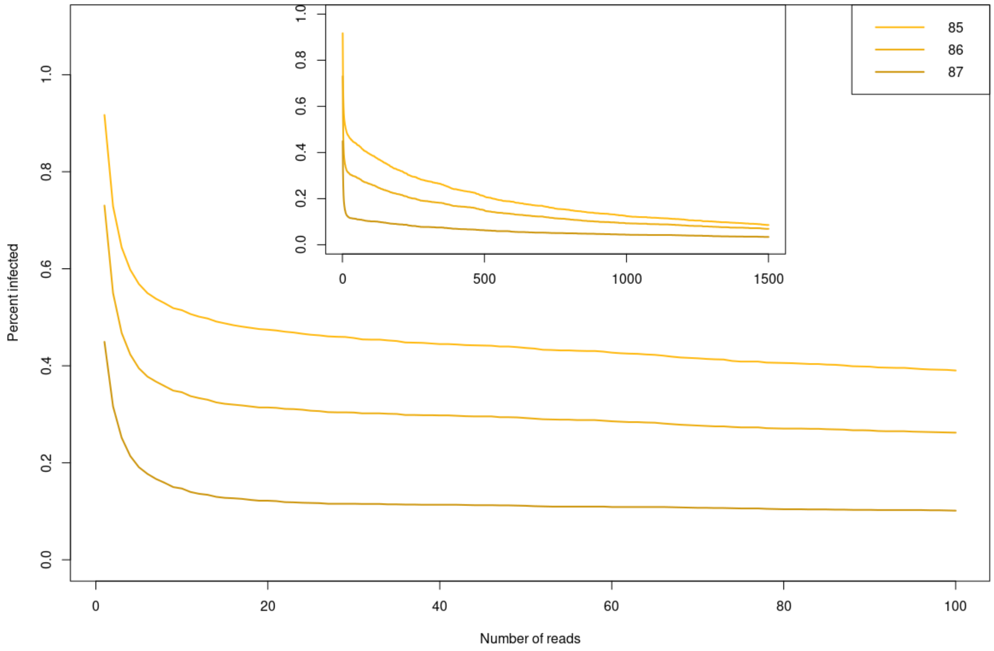

Supplementary Figure 2: Plots indicating infection percentage in a population as a function of the length (color) and number of reads (X-axis)

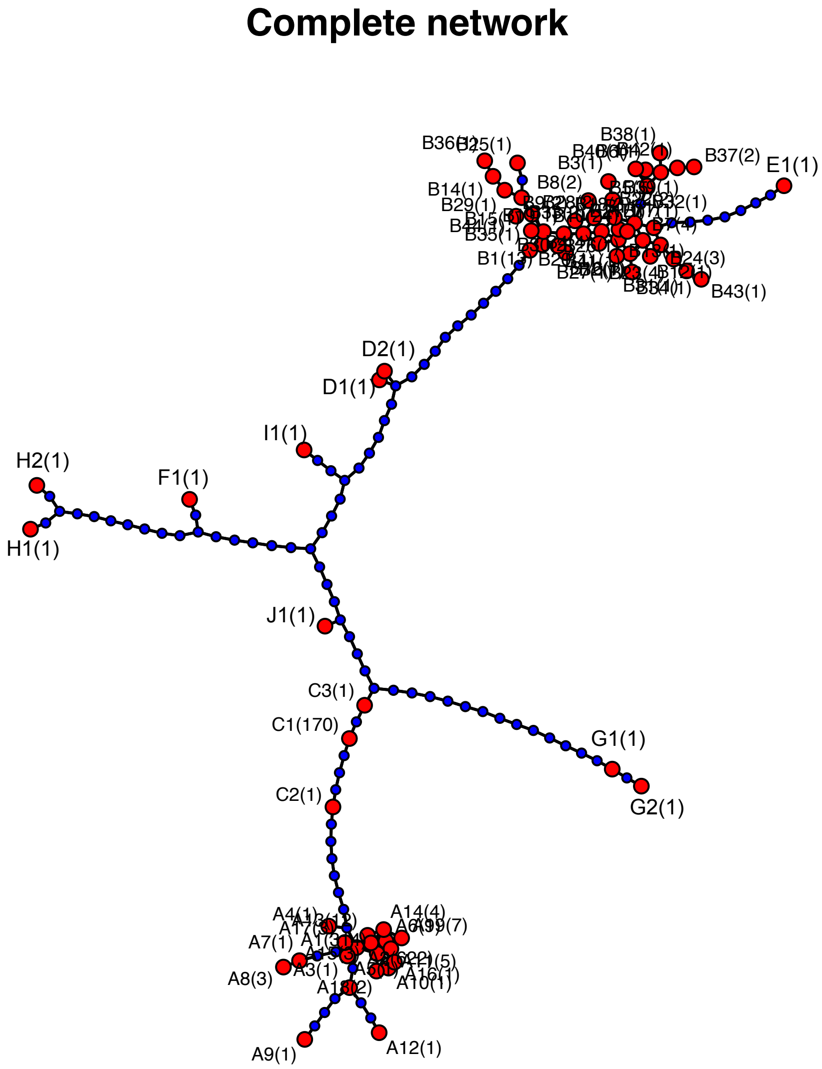

Supplementary Figure 3: All haplotypes shown in one parsimony network with number of individuals in parenthesis (statistical parsimony limit of 55%).

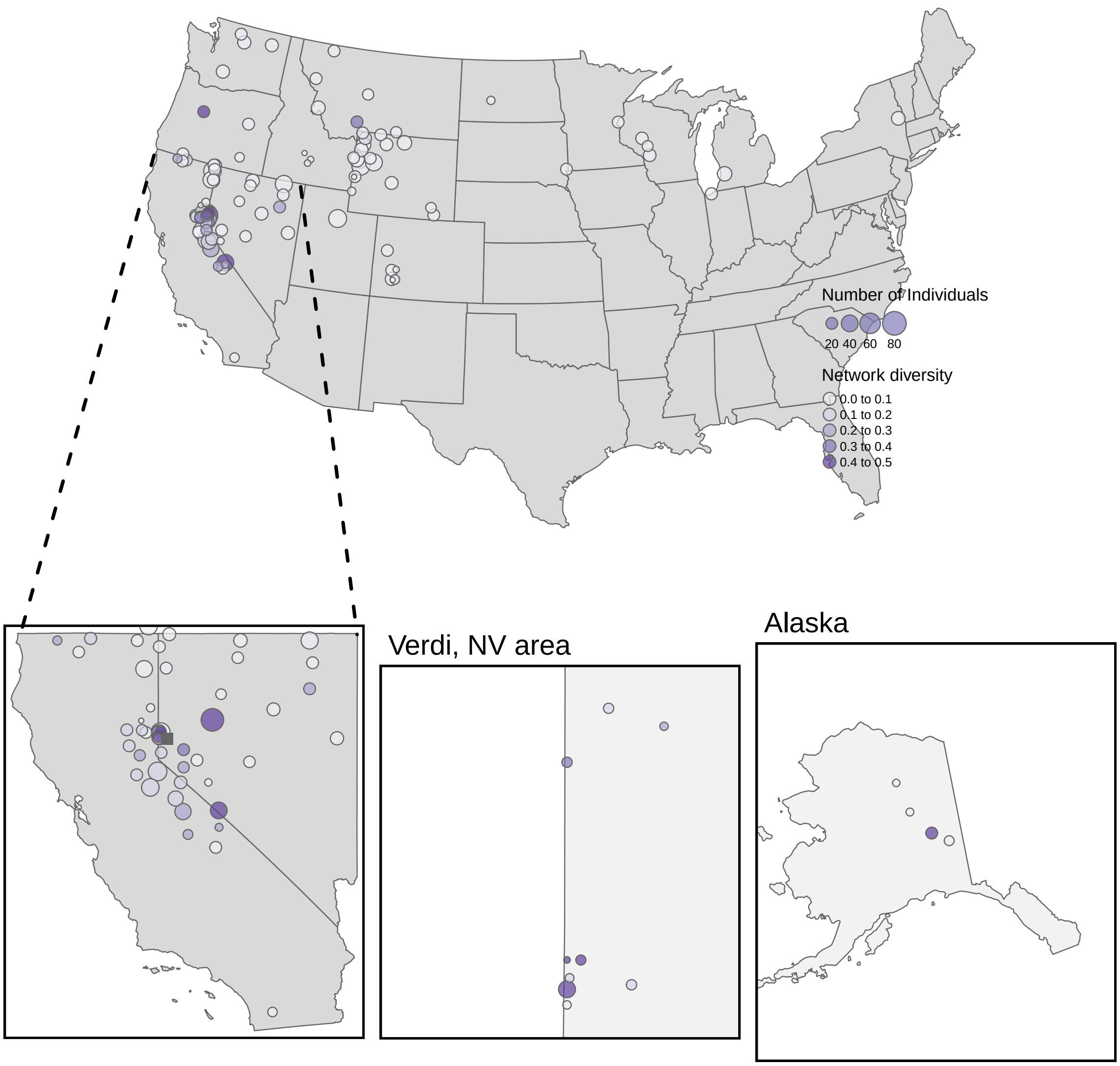

Supplementary Figure 4: Bubble plots showing the *Wolbachia* strain diversity (i.e., expected population heterozygosity or Gini-Simpson index) for each population, with warmer colors representing higher diversity and bubbles scaled by sample size.

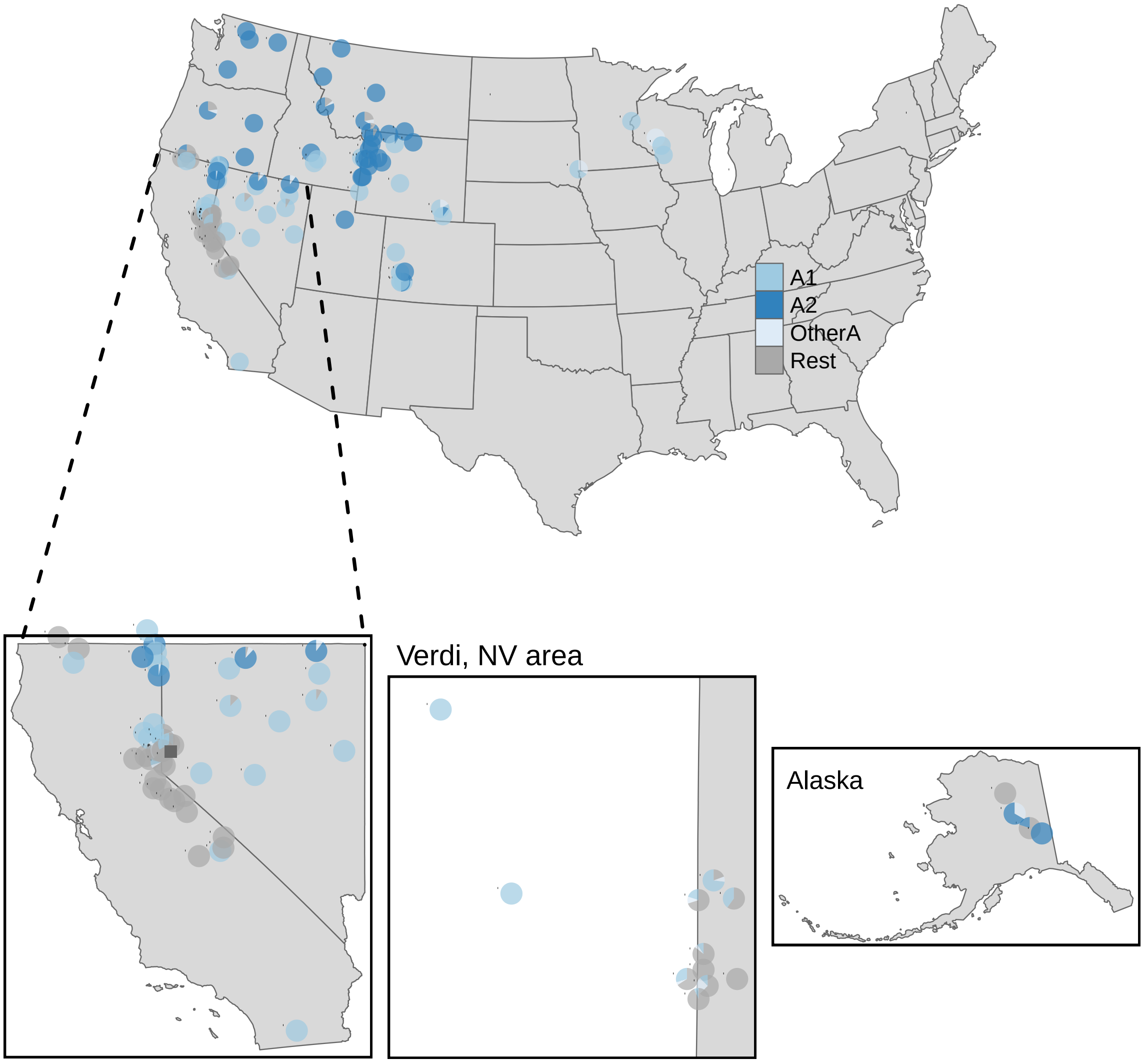

Supplementary Figure 5: Pie charts showing the distribution of haplotypes from strain *wLyc*A across the 107 sampled locations for the concatenated reference genome. Haplotypes A1 and A2 are present in 97.2% of individuals infected with strain *wLyc*A. The label 'OtherA' corresponds to rare haplotypes in *wLyc*A (A3-A9), and the label 'Rest' corresponds to individuals not infected by strain *wLyc*A (i.e., individuals infected with strains *wLyc*B and *wLyc*C, but not uninfected individuals). Pie charts for each locality are scaled by sample size. Inset plots zoomed in to regions of interest for visibility.

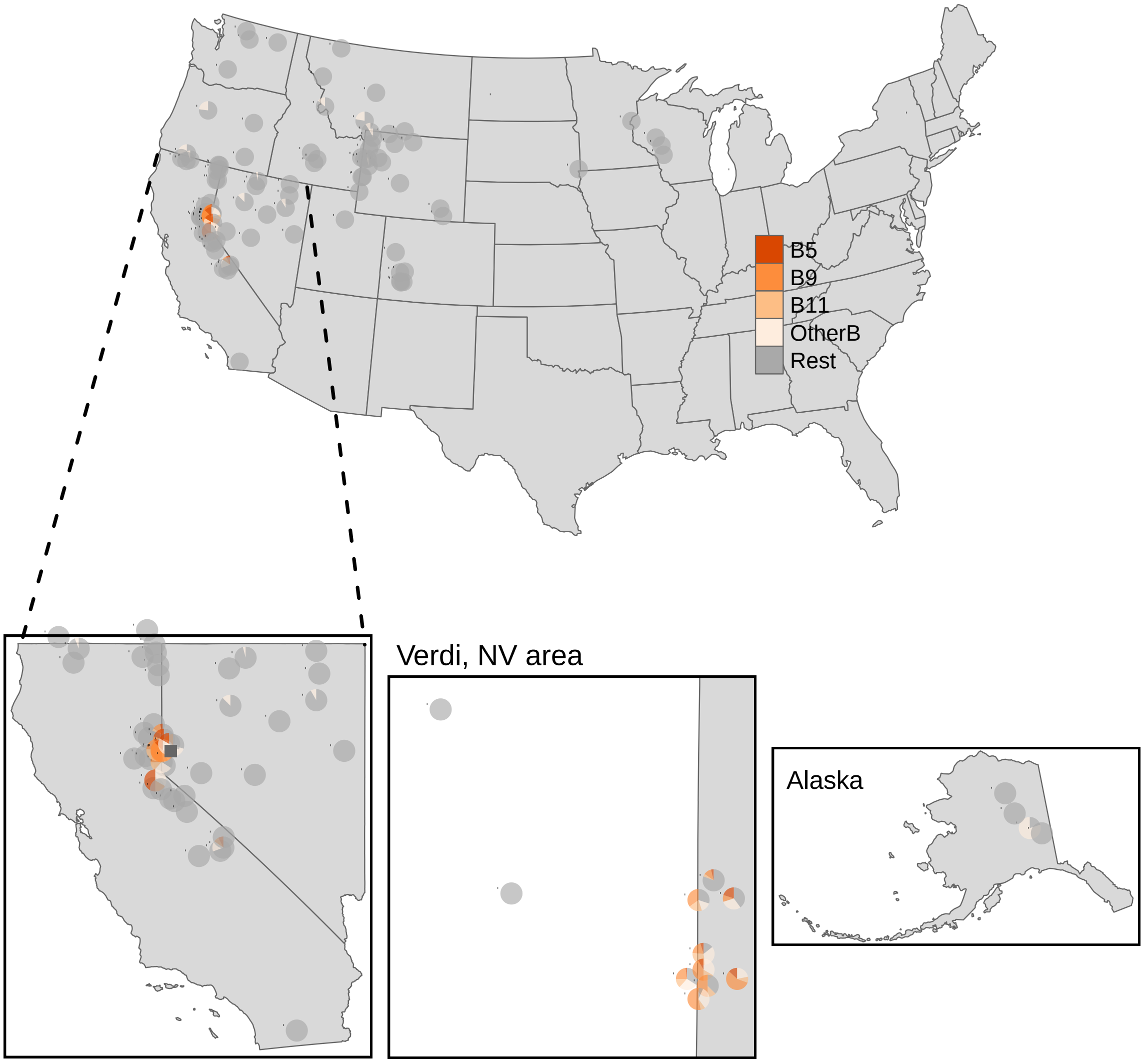

Supplementary Figure 6: Pie charts showing the distribution of haplotypes from strain *wLyc*B, scaled by sample size across the 107 sampled locations when the concatenated reference genome is used. Haplotypes B5, B9 and B11 make up 53% of all infections in the B strain. Inset plots zoomed in to regions of interest for visibility.

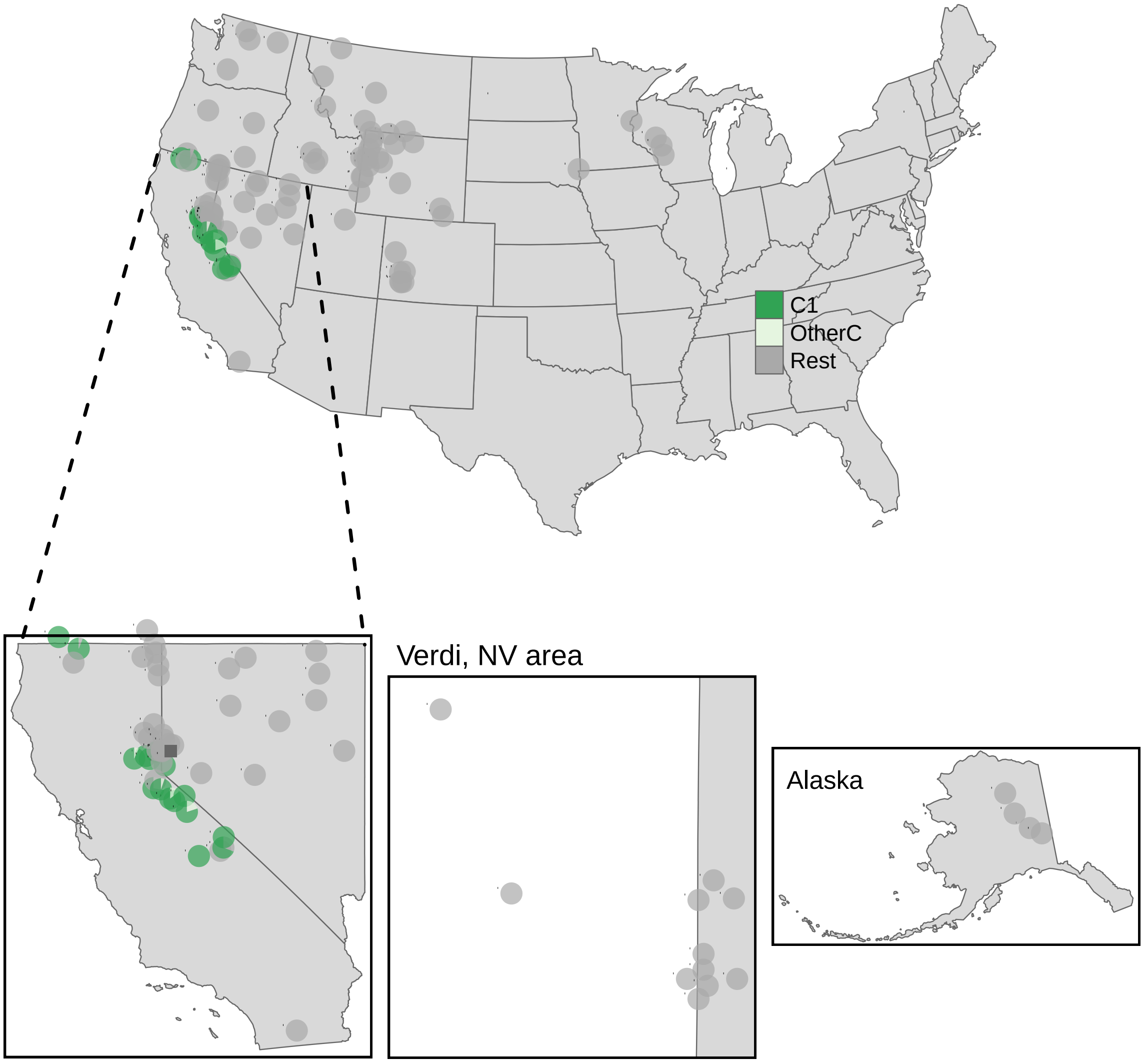

Supplementary Figure 7: Pie charts showing the distribution of haplotypes from strain *wLyc*C, scaled by sample size across the 107 sampled locations for the concatenated reference genome. Haplotype C1 makes up for 97% of all infections in the C strain, and all other C haplotypes are found in localities that also include haplotype C1.

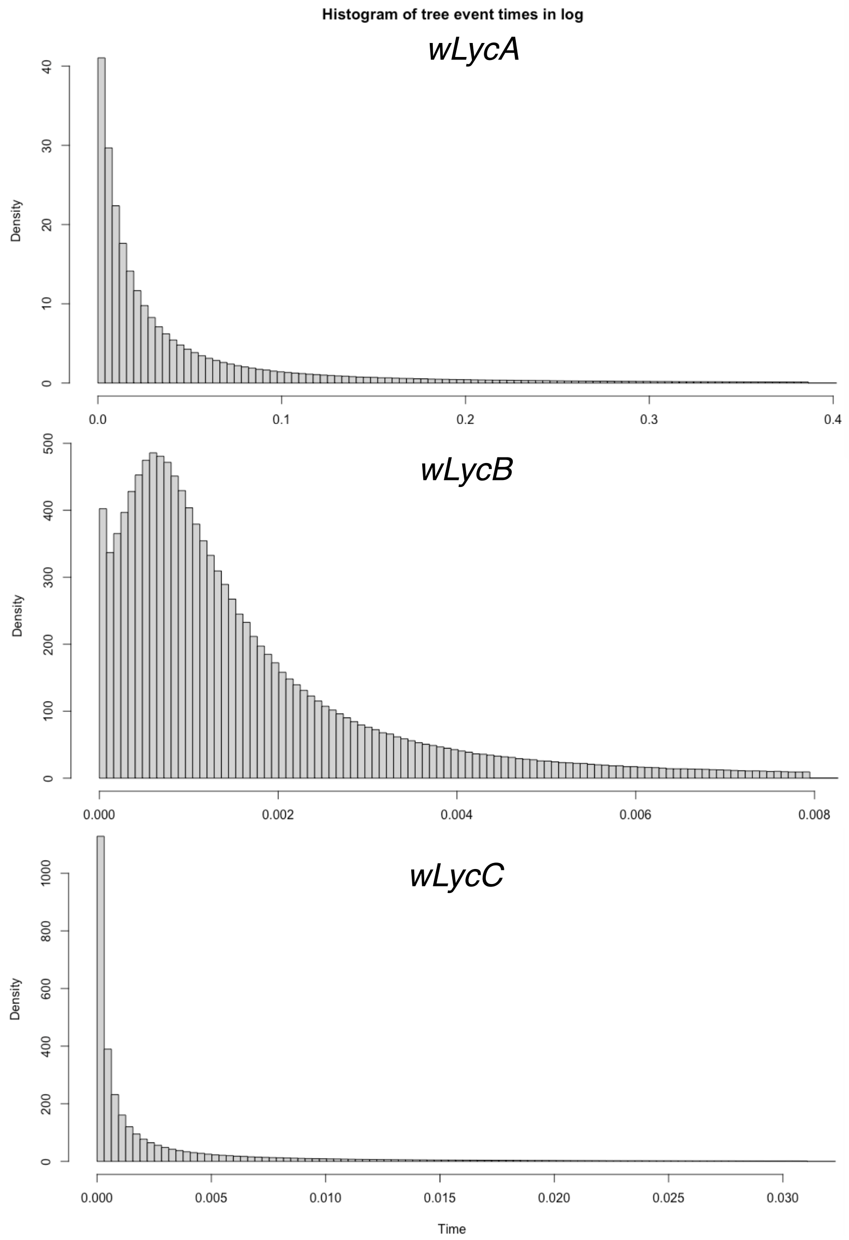

Supplementary Figure 8: Histogram of 95% of tree event times as recorded in the log during the BEAST run. There are barely any events beyond the right limit on the X-axis to indicate population size changes for each network. For example, most population size changes in *wLyc*C happen between 0 and 0.0025 time units as evidenced by the right skew. We also see that only *wLyc*B shows a population size change at an appreciable time in the past (non-zero) as compared to the other two networks.

Supplementary Table 1: Supplemental sample information for 107 *Lycaeides* collection localities. Table includes information on: locality number (corresponding to Fig . 1), locality names, nominal species designations (see text for details), year of collection, locality coordinates, number of individuals sampled, nucleotide diversity (as π), and *Wolbachia* strain diversity. The nominal species column includes some geographic divisions (e.g. "L. melissa - East") that represent substantial subdivisions within nominal taxa (see Gompert *et al.* (2014), Chaturvedi *et al.* (2018)). "NA" for some localities indicates that no genotype information was available for these localities. Diversity measures the variation in strain types within localities (see main text for details).

#	Locality	Nominal Species	Year	Lat. N	Long. W	n	Strain	Diversity
1	Fish Lk	L. samuelis	1996	45.74	-92.78	20	A(14)	0
2	Eau Claire	L. samuelis	1996	44.83	-91.23	22	A(3)	0
3	Black River	L. samuelis	1996	44.42	-90.90	17	A(14)	0
4	Fort McCoy	L. samuelis	1998	43.96	-90.83	23	A(21)	0
5	Indiana Dunes	L. samuelis	1999	41.67	-87.05	21	NA	NA
6	Allegan	L. samuelis	1998, 2009	42.53	-85.97	30	NA	NA
7	Saratoga Spr.s	L. samuelis	1999	43.06	-73.65	27	NA	NA
8	Fall Cr	L. anna	2011, 2012	39.38	-120.67	20	C(11)	0
9	Yuba Gap	L. anna	2012	39.32	-120.60	20	C(14),J(1)	0.1244
10	Castle Pk	L. anna	2008	39.37	-120.35	18	C(9)	0
11	Donner Pass	L. anna	2002, 2005	39.31	-120.35	18	C(4)	0
12	Marlette Lk	L. anna	2014, 2015	39.16	-119.91	19	C(9)	0
13	Leek Spr.s	L. anna	2012	38.63	-120.24	20	C(18)	0
14	Cottonwood	L. idas	2015	48.17	-120.36	25	A(24)	0
15	White Mt.	L. idas	2015	48.36	-118.31	24	A(15)	0
16	StrawB Mt.s	L. idas	2012	44.34	-118.64	20	A(17)	0
17	Siyeh Cr	L. idas	2010	48.7	-113.67	20	A(14)	0
18	Soldier Cr	L. idas	2008, 2009	47.21	-114.61	20	A(12)	0
19	Tibbs Butte	L. idas	2012	44.95	-109.45	20	A(17)	0
20	King's Hill	L. idas	2008, 2009	46.84	-110.70	18	A(12)	0
21	Garnet Pk	L. idas	2010	45.43	-111.22	20	A(6),B(2)	0.3750
22	Shook Mtn	L. idas	2018	45.81	-114.08	28	A(18)	0
23	Wolftone Rd	L. idas	2018	43.51	-114.47	4	A(4)	0
24	Bunsen Pk	L. idas	2009, 2010	44.93	-110.72	20	A(11)	0
25	Hayden V	L. idas	2007, 2010	44.68	-110.49	22	A(11),B(1)	0.1528
26	Animas RH	L. idas	2001	37.93	-107.57	13	A(8)	0
27	Red Mt. P	L. idas	2002	37.9	-107.71	4	A(2)	0
28	Tomboy Rd	L. idas	2011	37.94	-107.77	24	A(12)	0
29	Nolan Rd	L. idas	2018	67.42	-150.12	8	NA	NA
30	Spruce Barley	L. idas	2018	63.98	-145.34	20	A(1),B(1)	0.5000
31	Tok	L. idas	2018	63.33	-142.99	14	A(2)	0
32	Tolovana Cr	L. idas	2018	65.48	-148.27	9	A(3)	0
33	Soda Mt.	L. ricei	2009	42.12	-122.48	20	A(12)	0
34	Rainy Pass	L. ricei	2015	48.52	-120.74	20	A(15)	0
35	Chinook Pass	L. ricei	2015	46.52	-121.31	25	A(17)	0
36	Big Lk	L. ricei	2012	44.38	-121.87	20	A(12),B(5)	0.4152
37	Cave Lk	L. ricei	2002, 2004, 2007, 2009	41.98	-120.21	24	A(21)	0
38	Marble Mts.	L. ricei	2007	41.83	-122.75	12	C(5),G(2)	0.4082
39	Shovel Cr	L. ricei	2011	41.88	-122.16	21	B(1),C(16)	0
40	Beulah	L. melissa - East	2015	47.02	-101.82	10	A(1)	0
41	Brandon	L. melissa - East	2003, 2008, 2009	43.59	-96.57	20	A(3)	0
42	Silver Cr	L. melissa - East	2014	43.25	-113.99	6	NA	NA
43	Richfield	L. melissa - East	2014	43.05	-114.15	6	A(2)	0

Supplementary Table 1 - *Continued from previous page*

#	Locality	Nominal Species	Year	Lat. N	Long. W	n	Strain	Diversity
44	Victor	L. melissa - East	2009, 2010	43.66	-111.11	20	A(11)	0
45	Cokeville	L. melissa - East	2012	42.01	-110.94	10	A(4)	0
46	Montrose	L. melissa - East	2011	38.37	-107.82	20	A(10)	0
47	De Beque	L. melissa - East	2012	39.32	-108.21	20	A(5)	0
48	Cimarron	L. melissa - East	2014	38.43	-107.54	6	A(2)	0
49	Goose Lake	L. melissa - East	2011	41.99	-120.29	20	A(7)	0
50	Montague	L. melissa - East	2007	41.77	-122.38	19	A(17)	0
51	Susanville	L. melissa - East	2014	40.12	-120.23	10	A(6)	0
52	Abel Cr	L. melissa - East	2012	41.42	-117.62	19	A(1)	0
53	Deeth	L. melissa - East	2011	41.3	-115.38	20	A(8)	0
54	Mill Cr	L. melissa - East	2015	40.19	-116.55	24	A(14)	0
55	East Cr CG	L. melissa - East	2014	39.5	-114.65	25	A(8)	0
56	Lamoille	L. melissa - East	2010, 2011	40.68	-115.47	20	A(10),B(2)	0.2778
57	Ophir City	L. melissa - East	2012	38.94	-117.27	19	A(8)	0
58	Star Cr	L. melissa - East	2012	40.55	-118.12	16	A(6)	0
59	Upper Alkali	L. melissa - East	2012	41.79	-120.17	20	A(8)	0
60	Surprise V	L. melissa - East	2011	41.28	-120.1	20	A(13)	0
61	Cody	L. melissa - Rockies	2012	44.51	-108.98	23	A(12)	0
62	Lander	L. melissa - Rockies	2010	42.65	-108.36	24	A(4)	0
63	Wheatland	L. melissa - Rockies	2018	41.6	-105.61	16	A(12),I(1)	0.1420
64	Yellow Pine CG	L. melissa - Rockies	2012	41.25	-105.4	20	A(10)	0
65	Albion Meadow	L. melissa - Rockies	2012	40.59	-111.62	46	A(40)	0
66	Lake Davis	L. melissa - West	2013	39.92	-120.51	4	A(2)	0
67	Sierravalley	L. melissa - West	2011	39.64	-120.37	20	A(2)	0
68	White Lk	L. melissa - West	2015	39.66	-119.97	27	A(21),B(2)	0.1588
69	Silver Lk	L. melissa - West	2012	39.65	-119.93	18	A(5),B(7)	0.4861
70	Girl Farm	L. melissa - West	2017	39.63	-120	27	A(6),B(11), D(1),E(1)	0.3789
71	Verdi Crystal	L. melissa - West	2010, 2011, 2012, 2017, 2018	39.51	-120	73	A(18),B(11), H(1)	0.5044
72	Verdi Classic	L. melissa - West	2017, 2018	39.52	-119.99	26	A(3),B(8)	0.3967
73	Verdi Tracks	L. melissa - West	2017	39.51	-119.99	20	B(10),H(1)	0.1653
74	Verdi Hwy	L. melissa - West	2017	39.51	-120	11	A(3),B(2)	0.4800
75	Qui	L. melissa - West	2017	39.49	-120	18	A(1),B(12)	0.1420
76	Deer Mt Rd	L. melissa - West	2017	39.51	-119.96	27	B(14)	0
77	Washoe Lk	L. melissa - West	2011, 2012	39.23	-119.78	20	A(2),B(1)	0.4444
78	Gardnerville	L. melissa - West	2001, 2004, 2007	38.81	-119.78	18	B(6),F(1)	0.2449
79	Red Earth	L. melissa - West	2011	38.98	-118.84	20	A(8)	0
80	Bishop	L. melissa - West	2011	37.17	-118.28	20	A(11)	0
81	Trout Pond	L. melissa - West	2010	32.98	-116.58	13	A(4)	0
82	Big Ice	hybrid - Jackson	2012	45.16	-108.4	18	A(11)	0
83	Blacktail Butte	hybrid - Jackson	2012, 2012	43.64	-110.68	46	A(32)	0
84	Bull Cr	hybrid - Jackson	2009, 2010, 2012	43.3	-110.55	46	A(27)	0
85	Dubois	hybrid - Jackson	2010, 2011, 2012	43.56	-109.7	41	A(30)	0
86	Hunt Mt.	hybrid - Jackson	2010, 2012	44.68	-107.75	30	A(24)	0
87	Periodic Spri	hybrid - Jackson	2012	42.75	-110.85	20	A(28)	0
88	Pinnacles Butte	hybrid - Jackson	2012	43.74	-109.98	20	A(17)	0
89	Rendezvous Mt.	hybrid - Jackson	2012	43.6	-110.88	32	A(28)	0
90	Riddle Lk	hybrid - Jackson	2012	44.36	-110.55	30	A(22)	0
91	Sheffield Cr	hybrid - Jackson	2012	44.1	-110.66	26	A(22)	0
92	Swift Cr	hybrid - Jackson	2012	42.73	-110.91	4	A(2)	0
93	Buck Mt	hybrid - Warner	2012	41.69	-120.29	44	A(29)	0
94	Eagle Pk	hybrid - Warner	2011	41.26	-120.22	40	A(33)	0
95	Steens Mt	hybrid - Warner	2009, 2012	42.66	-118.73	13	A(5)	0
96	Hinkley	hybrid?	2017	41.67	-117.54	26	A(24)	0
97	Jarbidge	hybrid?	2018	41.83	-115.47	42	A(39)	0
98	Mt Rose	hybrid - Sierra/Whites	2012	39.32	-119.93	52	NA	NA
99	Carson Pass	hybrid - Sierra/Whites	2012	38.71	-120.02	50	C(20)	0
100	Corey Pk	hybrid - Sierra/Whites	2012	38.45	-118.77	8	NA	NA
101	Sonora Pass	hybrid - Sierra/Whites	2006, 2011	38.33	-119.63	44	C(15)	0

Supplementary Table 1 - *Continued from previous page*

#	Locality	Nominal Species	Year	Lat. N	Long. W	n	Strain	Diversity
102	Lake Emma	hybrid - Sierra/Whites	2006	38.28	-119.48	33	C(8)	0
103	Sweetwater	hybrid - Sierra/Whites	2006	38.45	-119.33	23	C(10)	0
104	Tioga Crest	hybrid - Sierra/Whites	2006, 2011	37.97	-119.26	38	C(5)	0
105	South Fork	hybrid - Sierra/Whites	2006	37.21	-118.57	14	C(5)	0
106	County Line	hybrid - Sierra/Whites	2011	37.46	-118.19	40	B(7),C(18),D(1)	0.4467
107	Reed Flat	hybrid - Sierra/Whites	2011	37.38	-118.18	9	C(5)	0

Supplementary Table 2: Barcoded adaptors used during GBS library construction. Oligonucleotides are organized in pairs that are annealed to create the double stranded adaptors. For example, oligonucleotides 8bp-2 and 8bp-2B are annealed to make one adaptor. Each contains an 8-10bp unique identifier sequence and the EcoR1 restriction site. These are ligated onto genomic fragments along with an adaptor that corresponds to the Mse1 restriction site (that does not include unique identifier sequences): MseI1 5' GCA-GAAGACGGCATACGAGCTCTTCCGATCTG 3' and MseI2: 5' TACA-GATCGGAAGAGCTCGTATGCCGTCTTCTGCTTG 3'. Fragments with these adaptors can be amplified with Illumina primers: Illpcr1 (Forward): 5' AATGATACGGCGACCACCGAGATCTACACTCTTTCCCTA-CACGACGCTCTTCCGATCT 3', Illpcr2 (Reverse): 5' CAAGCAGAA-GACGGCATACGAGCTCTTCCGATCTGTAA 3'.

Oligo Name	Sequence
8bp-2	5′ AATTGTTGGCGTTAGATCGGAAGAGCGTCGTGTAGGGAAAGAGTGT 3′
8bp-2B	5′ CTCTTTCCCTACACGACGCTCTTCCGATCTAACGCCAAC 3′
10bp-2	5′ AATTGTTGGTTCTGGAGATCGGAAGAGCGTCGTGTAGGGAAAGAGTGT 3′
10bp-2B	5′ CTCTTTCCCTACACGACGCTCTTCCGATCTCCAGAACCAAC 3′
8bp-3	5′ AATTGTTGGACCGAGATCGGAAGAGCGTCGTGTAGGGAAAGAGTGT 3′
8bp-3B	5′ CTCTTTCCCTACACGACGCTCTTCCGATCTCGGTCCAAC 3′
9bp-3	5′ AATTGTTGGTACCTAGATCGGAAGAGCGTCGTGTAGGGAAAGAGTGT 3′
9bp-3B	5′ CTCTTTCCCTACACGACGCTCTTCCGATCTAGGTACCAAC 3′
10bp-3	5′ AATTGTTGGTTACCTAGATCGGAAGAGCGTCGTGTAGGGAAAGAGTGT 3′
10bp-3B	5′ CTCTTTCCCTACACGACGCTCTTCCGATCTAGGTAACCAAC 3′
8bp-4	5′ AATTGTTGCTGAGAGATCGGAAGAGCGTCGTGTAGGGAAAGAGTGT 3′
8bp-4B	5′ CTCTTTCCCTACACGACGCTCTTCCGATCTCTCAGCAAC 3′
9bp-4	5′ AATTGTTGGCTTCAAGATCGGAAGAGCGTCGTGTAGGGAAAGAGTGT 3′
9bp-4B	5′ CTCTTTCCCTACACGACGCTCTTCCGATCTTGAAGCCAAC 3′
8bp-5	5′ AATTGTTGCGTCTAGATCGGAAGAGCGTCGTGTAGGGAAAGAGTGT 3′
8bp-5B	5′ CTCTTTCCCTACACGACGCTCTTCCGATCTAGACGCAAC 3′
9bp-5	5′ AATTGTTGGCGGTTAGATCGGAAGAGCGTCGTGTAGGGAAAGAGTGT 3′
9bp-5B	5′ CTCTTTCCCTACACGACGCTCTTCCGATCTAACCGCCAAC 3′
10bp-5	5′ AATTGTTGGTCGCGAAGATCGGAAGAGCGTCGTGTAGGGAAAGAGTGT 3′
10bp-5B	5′ CTCTTTCCCTACACGACGCTCTTCCGATCTTCGCGACCAAC 3′
8bp-6	5′ AATTGTTGCCAGAAGATCGGAAGAGCGTCGTGTAGGGAAAGAGTGT 3′
8bp-6B	5′ CTCTTTCCCTACACGACGCTCTTCCGATCTTCTGGCAAC 3′
9bp-6	5′ AATTGTTGGCCAACAGATCGGAAGAGCGTCGTGTAGGGAAAGAGTGT 3′
9bp-6B	5′ CTCTTTCCCTACACGACGCTCTTCCGATCTGTTGGCCAAC 3′
10bp-6	5′ AATTGTTGGTCCGATAGATCGGAAGAGCGTCGTGTAGGGAAAGAGTGT 3′
10bp-6B	5′ CTCTTTCCCTACACGACGCTCTTCCGATCTATCGGACCAAC 3′
8bp-7	5′ AATTGTTGATCTAAGATCGGAAGAGCGTCGTGTAGGGAAAGAGTGT 3′
8bp-7B	5′ CTCTTTCCCTACACGACGCTCTTCCGATCTTAGATCAAC 3′
9bp-7	5′ AATTGTTGGATCAGAGATCGGAAGAGCGTCGTGTAGGGAAAGAGTGT 3′
9bp-7B	5′ CTCTTTCCCTACACGACGCTCTTCCGATCTCTGATCCAAC 3′
10bp-7	5′ AATTGTTGGTCAATGAGATCGGAAGAGCGTCGTGTAGGGAAAGAGTGT 3′
10bp-7B	5′ CTCTTTCCCTACACGACGCTCTTCCGATCTCATTGACCAAC 3′
8bp-8	5′ AATTGTTGAGAACAGATCGGAAGAGCGTCGTGTAGGGAAAGAGTGT 3′
8bp-8B	5′ CTCTTTCCCTACACGACGCTCTTCCGATCTGTTCTCAAC 3′
9bp-8	5′ AATTGTTGGAGAGAAGATCGGAAGAGCGTCGTGTAGGGAAAGAGTGT 3′
9bp-8B	5′ CTCTTTCCCTACACGACGCTCTTCCGATCTTCTCTCCAAC 3′
10bp-8	5′ AATTGTTGGTATCAGAGATCGGAAGAGCGTCGTGTAGGGAAAGAGTGT 3′
10bp-8B	5′ CTCTTTCCCTACACGACGCTCTTCCGATCTCTGATACCAAC 3′
9bp-9	5′ AATTGTTGCTTATTAGATCGGAAGAGCGTCGTGTAGGGAAAGAGTGT 3′
9bp-9B	5′ CTCTTTCCCTACACGACGCTCTTCCGATCTAATAAGCAAC 3′

Supplementary Table 2 - *Continued from previous page*

**Oligo Name**	**Sequence**
10bp-9	5′ AATTGTTGGCTTGGTAGATCGGAAGAGCGTCGTGTAGGGAAAGAGTGT 3′
10bp-9B	5′ CTCTTTCCCTACACGACGCTCTTCCGATCTACCAAGCCAAC 3′
8bp-10	5′ AATTGTTCTGCTGAGATCGGAAGAGCGTCGTGTAGGGAAAGAGTGT 3′
8bp-10B	5′ CTCTTTCCCTACACGACGCTCTTCCGATCTCAGCAGAAC 3′
9bp-10	5′ AATTGTTGCTGGCGAGATCGGAAGAGCGTCGTGTAGGGAAAGAGTGT 3′
9bp-10B	5′ CTCTTTCCCTACACGACGCTCTTCCGATCTCGCCAGCAAC 3′
10bp-10	5′ AATTGTTGGCTGACGAGATCGGAAGAGCGTCGTGTAGGGAAAGAGTGT 3′
10bp-10B	5′ CTCTTTCCCTACACGACGCTCTTCCGATCTCGTCAGCCAAC 3′
8bp-11	5′ AATTGTTCTCTCAAGATCGGAAGAGCGTCGTGTAGGGAAAGAGTGT 3′
8bp-11B	5′ CTCTTTCCCTACACGACGCTCTTCCGATCTTGAGAGAAC 3′
9bp-11	5′ AATTGTTGCTCCGAAGATCGGAAGAGCGTCGTGTAGGGAAAGAGTGT 3′
9bp-11B	5′ CTCTTTCCCTACACGACGCTCTTCCGATCTTCGGAGCAAC 3′
10bp-11	5′ AATTGTTGGCTCCTAAGATCGGAAGAGCGTCGTGTAGGGAAAGAGTGT 3′
10bp-11B	5′ CTCTTTCCCTACACGACGCTCTTCCGATCTTAGGAGCCAAC 3′
8bp-12	5′ AATTGTTCGAGACAGATCGGAAGAGCGTCGTGTAGGGAAAGAGTGT 3′
8bp-12B	5′ CTCTTTCCCTACACGACGCTCTTCCGATCTGTCTCGAAC 3′
9bp-12	5′ AATTGTTGCTATACAGATCGGAAGAGCGTCGTGTAGGGAAAGAGTGT 3′
9bp-12B	5′ CTCTTTCCCTACACGACGCTCTTCCGATCTGTATAGCAAC 3′
10bp-12	5′ AATTGTTGGCTATACAGATCGGAAGAGCGTCGTGTAGGGAAAGAGTGT 3′
10bp-12B	5′ CTCTTTCCCTACACGACGCTCTTCCGATCTGTATAGCCAAC 3′
8bp-13	5′ AATTGTTCCTACCAGATCGGAAGAGCGTCGTGTAGGGAAAGAGTGT 3′
8bp-13B	5′ CTCTTTCCCTACACGACGCTCTTCCGATCTGGTAGGAAC 3′
9bp-13	5′ AATTGTTGCGTTGGAGATCGGAAGAGCGTCGTGTAGGGAAAGAGTGT 3′
9bp-13B	5′ CTCTTTCCCTACACGACGCTCTTCCGATCTCCAACGCAAC 3′
10bp-13	5′ AATTGTTGGCGTATCAGATCGGAAGAGCGTCGTGTAGGGAAAGAGTGT 3′
10bp-13B	5′ CTCTTTCCCTACACGACGCTCTTCCGATCTGATACGCCAAC 3′
8bp-14	5′ AATTGTTCCATGGAGATCGGAAGAGCGTCGTGTAGGGAAAGAGTGT 3′
8bp-14B	5′ CTCTTTCCCTACACGACGCTCTTCCGATCTCCATGGAAC 3′
9bp-14	5′ AATTGTTGCGGCTCAGATCGGAAGAGCGTCGTGTAGGGAAAGAGTGT 3′
9bp-14B	5′ CTCTTTCCCTACACGACGCTCTTCCGATCTGAGCCGCAAC 3′
10bp-16	5′ AATTGTTGGATAAGAAGATCGGAAGAGCGTCGTGTAGGGAAAGAGTGT 3′
10bp-16B	5′ CTCTTTCCCTACACGACGCTCTTCCGATCTTCTTATCCAAC 3′
8bp-15	5′ AATTGTTATGGCCAGATCGGAAGAGCGTCGTGTAGGGAAAGAGTGT 3′
8bp-15B	5′ CTCTTTCCCTACACGACGCTCTTCCGATCTGGCCATAAC 3′
9bp-15	5′ AATTGTTGCGCGATAGATCGGAAGAGCGTCGTGTAGGGAAAGAGTGT 3′
9bp-15B	5′ CTCTTTCCCTACACGACGCTCTTCCGATCTATCGCGCAAC 3′
10bp-17	5′ AATTGTTGGAGTTAAAGATCGGAAGAGCGTCGTGTAGGGAAAGAGTGT 3′
10bp-17B	5′ CTCTTTCCCTACACGACGCTCTTCCGATCTTTAACTCCAAC 3′
8bp-16	5′ AATTGTTATCCATAGATCGGAAGAGCGTCGTGTAGGGAAAGAGTGT 3′
8bp-16B	5′ CTCTTTCCCTACACGACGCTCTTCCGATCTATGGATAAC 3′
9bp-16	5′ AATTGTTGCAAGTAAGATCGGAAGAGCGTCGTGTAGGGAAAGAGTGT 3′
9bp-16B	5′ CTCTTTCCCTACACGACGCTCTTCCGATCTTACTTGCAAC 3′
10bp-18	5′ AATTGTTGGAGCGGCAGATCGGAAGAGCGTCGTGTAGGGAAAGAGTGT 3′
10bp-18B	5′ CTCTTTCCCTACACGACGCTCTTCCGATCTGCCGCTCCAAC 3′
8bp-17	5′ AATTGTTAGTATGAGATCGGAAGAGCGTCGTGTAGGGAAAGAGTGT 3′
8bp-17B	5′ CTCTTTCCCTACACGACGCTCTTCCGATCTCATACTAAC 3′
9bp-17	5′ AATTGTTGATAAGGAGATCGGAAGAGCGTCGTGTAGGGAAAGAGTGT 3′
9bp-17B	5′ CTCTTTCCCTACACGACGCTCTTCCGATCTCCTTATCAAC 3′
10bp-23	5′ AATTGTTGCTTCGCAAGATCGGAAGAGCGTCGTGTAGGGAAAGAGTGT 3′
10bp-23B	5′ CTCTTTCCCTACACGACGCTCTTCCGATCTTGCGAAGCAAC 3′
8bp-18	5′ AATTGTTAGGTAAAGATCGGAAGAGCGTCGTGTAGGGAAAGAGTGT 3′
8bp-18B	5′ CTCTTTCCCTACACGACGCTCTTCCGATCTTTACCTAAC 3′
9bp-18	5′ AATTGTTGAGTACCAGATCGGAAGAGCGTCGTGTAGGGAAAGAGTGT 3′
9bp-18B	5′ CTCTTTCCCTACACGACGCTCTTCCGATCTGGTACTCAAC 3′
10bp-24	5′ AATTGTTGCTGGTTAAGATCGGAAGAGCGTCGTGTAGGGAAAGAGTGT 3′
10bp-24B	5′ CTCTTTCCCTACACGACGCTCTTCCGATCTTAACCAGCAAC 3′
8bp-19	5′ AATTGTTACCTTCAGATCGGAAGAGCGTCGTGTAGGGAAAGAGTGT 3′
8bp-19B	5′ CTCTTTCCCTACACGACGCTCTTCCGATCTGAAGGTAAC 3′
9bp-19	5′ AATTGTTGAGGTAAAGATCGGAAGAGCGTCGTGTAGGGAAAGAGTGT 3′
9bp-19B	5′ CTCTTTCCCTACACGACGCTCTTCCGATCTTTACCTCAAC 3′
10bp-25	5′ AATTGTTGCTGCAAGAGATCGGAAGAGCGTCGTGTAGGGAAAGAGTGT 3′
10bp-25B	5′ CTCTTTCCCTACACGACGCTCTTCCGATCTCTTGCAGCAAC 3′

Supplementary Table 2 - *Continued from previous page*

**Oligo Name**	**Sequence**
9bp-20	5′ AATTGTTGACTCGTAGATCGGAAGAGCGTCGTGTAGGGAAAGAGTGT 3′
9bp-20B	5′ CTCTTTCCCTACACGACGCTCTTCCGATCTACGAGTCAAC 3′
10bp-31	5′ AATTGTTGCGGTTCTAGATCGGAAGAGCGTCGTGTAGGGAAAGAGTGT 3′
10bp-31B	5′ CTCTTTCCCTACACGACGCTCTTCCGATCTAGAACCGCAAC 3′
8bp-21	5′ AATTGTGGTCGACAGATCGGAAGAGCGTCGTGTAGGGAAAGAGTGT 3′
8bp-21B	5′ CTCTTTCCCTACACGACGCTCTTCCGATCTGTCGACCAC 3′
9bp-21	5′ AATTGTTGAACTGCAGATCGGAAGAGCGTCGTGTAGGGAAAGAGTGT 3′
9bp-21B	5′ CTCTTTCCCTACACGACGCTCTTCCGATCTGCAGTTCAAC 3′
10bp-37	5′ AATTGTTGCCAGGCTAGATCGGAAGAGCGTCGTGTAGGGAAAGAGTGT 3′
10bp-37B	5′ CTCTTTCCCTACACGACGCTCTTCCGATCTAGCCTGGCAAC 3′
8bp-22	5′ AATTGTGGTAATTAGATCGGAAGAGCGTCGTGTAGGGAAAGAGTGT 3′
8bp-22B	5′ CTCTTTCCCTACACGACGCTCTTCCGATCTAATTACCAC 3′
10bp-38	5′ AATTGTTGCATTACCAGATCGGAAGAGCGTCGTGTAGGGAAAGAGTGT 3′
10bp-38B	5′ CTCTTTCCCTACACGACGCTCTTCCGATCTGGTAATGCAAC 3′
8bp-23	5′ AATTGTGGATTATAGATCGGAAGAGCGTCGTGTAGGGAAAGAGTGT 3′
8bp-23B	5′ CTCTTTCCCTACACGACGCTCTTCCGATCTATAATCCAC 3′
9bp-23	5′ AATTGTTCTTCTCAAGATCGGAAGAGCGTCGTGTAGGGAAAGAGTGT 3′
9bp-23B	5′ CTCTTTCCCTACACGACGCTCTTCCGATCTTGAGAAGAAC 3′
10bp-41	5′ AATTGTTGATGGACCAGATCGGAAGAGCGTCGTGTAGGGAAAGAGTGT 3′
10bp-41B	5′ CTCTTTCCCTACACGACGCTCTTCCGATCTGGTCCATCAAC 3′
8bp-24	5′ AATTGTGGAGGCGAGATCGGAAGAGCGTCGTGTAGGGAAAGAGTGT 3′
8bp-24B	5′ CTCTTTCCCTACACGACGCTCTTCCGATCTCGCCTCCAC 3′
9bp-24	5′ AATTGTTCTGGCCTAGATCGGAAGAGCGTCGTGTAGGGAAAGAGTGT 3′
9bp-24B	5′ CTCTTTCCCTACACGACGCTCTTCCGATCTAGGCCAGAAC 3′
10bp-43	5′ AATTGTTGATATGGAAGATCGGAAGAGCGTCGTGTAGGGAAAGAGTGT 3′
10bp-43B	5′ CTCTTTCCCTACACGACGCTCTTCCGATCTTCCATATCAAC 3′
8bp-25	5′ AATTGTGCGTTCGAGATCGGAAGAGCGTCGTGTAGGGAAAGAGTGT 3′
8bp-25B	5′ CTCTTTCCCTACACGACGCTCTTCCGATCTCGAACGCAC 3′
9bp-25	5′ AATTGTTCTGCATGAGATCGGAAGAGCGTCGTGTAGGGAAAGAGTGT 3′
9bp-25B	5′ CTCTTTCCCTACACGACGCTCTTCCGATCTCATGCAGAAC 3′
10bp-47	5′ AATTGTTGAGCGTAAAGATCGGAAGAGCGTCGTGTAGGGAAAGAGTGT 3′
10bp-47B	5′ CTCTTTCCCTACACGACGCTCTTCCGATCTTTACGCTCAAC 3′
8bp-26	5′ AATTGTGCGGCGTAGATCGGAAGAGCGTCGTGTAGGGAAAGAGTGT 3′
8bp-26B	5′ CTCTTTCCCTACACGACGCTCTTCCGATCTACGCCGCAC 3′
9bp-26	5′ AATTGTTCTGATGCAGATCGGAAGAGCGTCGTGTAGGGAAAGAGTGT 3′
9bp-26B	5′ CTCTTTCCCTACACGACGCTCTTCCGATCTGCATCAGAAC 3′
8bp-27	5′ AATTGTGCGCATCAGATCGGAAGAGCGTCGTGTAGGGAAAGAGTGT 3′
8bp-27B	5′ CTCTTTCCCTACACGACGCTCTTCCGATCTGATGCGCAC 3′
9bp-27	5′ AATTGTTCTCTTATAGATCGGAAGAGCGTCGTGTAGGGAAAGAGTGT 3′
9bp-27B	5′ CTCTTTCCCTACACGACGCTCTTCCGATCTATAAGAGAAC 3′
10bp-56	5′ AATTGTTCTTGCGTCAGATCGGAAGAGCGTCGTGTAGGGAAAGAGTGT 3′
10bp-56B	5′ CTCTTTCCCTACACGACGCTCTTCCGATCTGACGCAAGAAC 3′
8bp-28	5′ AATTGTGCCGTACAGATCGGAAGAGCGTCGTGTAGGGAAAGAGTGT 3′
8bp-28B	5′ CTCTTTCCCTACACGACGCTCTTCCGATCTGTACGGCAC 3′
9bp-28	5′ AATTGTTCTCAGCGAGATCGGAAGAGCGTCGTGTAGGGAAAGAGTGT 3′
9bp-28B	5′ CTCTTTCCCTACACGACGCTCTTCCGATCTCGCTGAGAAC 3′
10bp-57	5′ AATTGTTCTTGACGGAGATCGGAAGAGCGTCGTGTAGGGAAAGAGTGT 3′
10bp-57B	5′ CTCTTTCCCTACACGACGCTCTTCCGATCTCCGTCAAGAAC 3′
8bp-29	5′ AATTGTGATGAAGAGATCGGAAGAGCGTCGTGTAGGGAAAGAGTGT 3′
8bp-29B	5′ CTCTTTCCCTACACGACGCTCTTCCGATCTCTTCATCAC 3′
9bp-29	5′ AATTGTTCTATGGAAGATCGGAAGAGCGTCGTGTAGGGAAAGAGTGT 3′
9bp-29B	5′ CTCTTTCCCTACACGACGCTCTTCCGATCTTCCATAGAAC 3′
10bp-58	5′ AATTGTTCTTCTTGAAGATCGGAAGAGCGTCGTGTAGGGAAAGAGTGT 3′
10bp-58B	5′ CTCTTTCCCTACACGACGCTCTTCCGATCTTCAAGAAGAAC 3′
8bp-30	5′ AATTGTGATATGCAGATCGGAAGAGCGTCGTGTAGGGAAAGAGTGT 3′
8bp-30B	5′ CTCTTTCCCTACACGACGCTCTTCCGATCTGCATATCAC 3′
9bp-30	5′ AATTGTTCGTTCTAAGATCGGAAGAGCGTCGTGTAGGGAAAGAGTGT 3′
9bp-30B	5′ CTCTTTCCCTACACGACGCTCTTCCGATCTTAGAACGAAC 3′
10bp-59	5′ AATTGTTCTTCGGAGAGATCGGAAGAGCGTCGTGTAGGGAAAGAGTGT 3′
10bp-59B	5′ CTCTTTCCCTACACGACGCTCTTCCGATCTCTCCGAAGAAC 3′
8bp-31	5′ AATTGTGAGCGGAAGATCGGAAGAGCGTCGTGTAGGGAAAGAGTGT 3′
8bp-31B	5′ CTCTTTCCCTACACGACGCTCTTCCGATCTTCCGCTCAC 3′

Supplementary Table 2 - *Continued from previous page*

**Oligo Name**	**Sequence**
9bp-31	5′ AATTGTTCGTCAGTAGATCGGAAGAGCGTCGTGTAGGGAAAGAGTGT 3′
9bp-31B	5′ CTCTTTCCCTACACGACGCTCTTCCGATCTACTGACGAAC 3′
10bp-60	5′ AATTGTTCTTCAACCAGATCGGAAGAGCGTCGTGTAGGGAAAGAGTGT 3′
10bp-60B	5′ CTCTTTCCCTACACGACGCTCTTCCGATCTGGTTGAAGAAC 3′
8bp-32	5′ AATTGTGACTGCTAGATCGGAAGAGCGTCGTGTAGGGAAAGAGTGT 3′
8bp-32B	5′ CTCTTTCCCTACACGACGCTCTTCCGATCTAGCAGTCAC 3′
9bp-32	5′ AATTGTTCGGCGCCAGATCGGAAGAGCGTCGTGTAGGGAAAGAGTGT 3′
9bp-32B	5′ CTCTTTCCCTACACGACGCTCTTCCGATCTGGCGCCGAAC 3′
10bp-62	5′ AATTGTTCTTACCAAAGATCGGAAGAGCGTCGTGTAGGGAAAGAGTGT 3′
10bp-62B	5′ CTCTTTCCCTACACGACGCTCTTCCGATCTTTGGTAAGAAC 3′
8bp-33	5′ AATTGTGAAGCTCAGATCGGAAGAGCGTCGTGTAGGGAAAGAGTGT 3′
8bp-33B	5′ CTCTTTCCCTACACGACGCTCTTCCGATCTGAGCTTCAC 3′
9bp-33	5′ AATTGTTCGCGCGGAGATCGGAAGAGCGTCGTGTAGGGAAAGAGTGT 3′
9bp-33B	5′ CTCTTTCCCTACACGACGCTCTTCCGATCTCCGCGCGAAC 3′
10bp-63	5′ AATTGTTCTGGTCCAAGATCGGAAGAGCGTCGTGTAGGGAAAGAGTGT 3′
10bp-63B	5′ CTCTTTCCCTACACGACGCTCTTCCGATCTTGGACCAGAAC 3′
8bp-34	5′ AATTGTCTTGCGCAGATCGGAAGAGCGTCGTGTAGGGAAAGAGTGT 3′
8bp-34B	5′ CTCTTTCCCTACACGACGCTCTTCCGATCTGCGCAAGAC 3′
9bp-34	5′ AATTGTTCGAATAAAGATCGGAAGAGCGTCGTGTAGGGAAAGAGTGT 3′
9bp-34B	5′ CTCTTTCCCTACACGACGCTCTTCCGATCTTTATTCGAAC 3′
10bp-104	5′ AATTGTTCAAGAGGAAGATCGGAAGAGCGTCGTGTAGGGAAAGAGTGT 3′
10bp-104B	5′ CTCTTTCCCTACACGACGCTCTTCCGATCTTCCTCTTGAAC 3′
9bp-35	5′ AATTGTTCCATTCCAGATCGGAAGAGCGTCGTGTAGGGAAAGAGTGT 3′
9bp-35B	5′ CTCTTTCCCTACACGACGCTCTTCCGATCTGGAATGGAAC 3′
10bp-114	5′ AATTGTTATGCGAGAAGATCGGAAGAGCGTCGTGTAGGGAAAGAGTGT 3′
10bp-114B	5′ CTCTTTCCCTACACGACGCTCTTCCGATCTTCTCGCATAAC 3′
8bp-36	5′ AATTGTCTTAGCTAGATCGGAAGAGCGTCGTGTAGGGAAAGAGTGT 3′
8bp-36B	5′ CTCTTTCCCTACACGACGCTCTTCCGATCTAGCTAAGAC 3′
9bp-36	5′ AATTGTTCCAGAATAGATCGGAAGAGCGTCGTGTAGGGAAAGAGTGT 3′
9bp-36B	5′ CTCTTTCCCTACACGACGCTCTTCCGATCTATTCTGGAAC 3′
10bp-130	5′ AATTGTTAGCAACTTAGATCGGAAGAGCGTCGTGTAGGGAAAGAGTGT 3′
10bp-130B	5′ CTCTTTCCCTACACGACGCTCTTCCGATCTAAGTTGCTAAC 3′
8bp-37	5′ AATTGTCTGGATTAGATCGGAAGAGCGTCGTGTAGGGAAAGAGTGT 3′
8bp-37B	5′ CTCTTTCCCTACACGACGCTCTTCCGATCTAATCCAGAC 3′
9bp-37	5′ AATTGTTCATTGCTAGATCGGAAGAGCGTCGTGTAGGGAAAGAGTGT 3′
9bp-37B	5′ CTCTTTCCCTACACGACGCTCTTCCGATCTAGCAATGAAC 3′
10bp-138	5′ AATTGTTACGACTATAGATCGGAAGAGCGTCGTGTAGGGAAAGAGTGT 3′
10bp-138B	5′ CTCTTTCCCTACACGACGCTCTTCCGATCTATAGTCGTAAC 3′
8bp-38	5′ AATTGTCTCTAGGAGATCGGAAGAGCGTCGTGTAGGGAAAGAGTGT 3′
8bp-38B	5′ CTCTTTCCCTACACGACGCTCTTCCGATCTCCTAGAGAC 3′
9bp-38	5′ AATTGTTCATCCAGAGATCGGAAGAGCGTCGTGTAGGGAAAGAGTGT 3′
9bp-38B	5′ CTCTTTCCCTACACGACGCTCTTCCGATCTCTGGATGAAC 3′
10bp-143	5′ AATTGTTAAGTTGCAAGATCGGAAGAGCGTCGTGTAGGGAAAGAGTGT 3′
10bp-143B	5′ CTCTTTCCCTACACGACGCTCTTCCGATCTTGCAACTTAAC 3′
8bp-39	5′ AATTGTCTATTCCAGATCGGAAGAGCGTCGTGTAGGGAAAGAGTGT 3′
8bp-39B	5′ CTCTTTCCCTACACGACGCTCTTCCGATCTGGAATAGAC 3′
9bp-39	5′ AATTGTTCAAGTTGAGATCGGAAGAGCGTCGTGTAGGGAAAGAGTGT 3′
9bp-39B	5′ CTCTTTCCCTACACGACGCTCTTCCGATCTCAACTTGAAC 3′
10bp-148	5′ AATTGTGGTTGGCTTAGATCGGAAGAGCGTCGTGTAGGGAAAGAGTGT 3′
10bp-148B	5′ CTCTTTCCCTACACGACGCTCTTCCGATCTAAGCCAACCAC 3′
8bp-40	5′ AATTGTCTACGAAAGATCGGAAGAGCGTCGTGTAGGGAAAGAGTGT 3′
8bp-40B	5′ CTCTTTCCCTACACGACGCTCTTCCGATCTTTCGTAGAC 3′
9bp-40	5′ AATTGTTATTGAAGAGATCGGAAGAGCGTCGTGTAGGGAAAGAGTGT 3′
9bp-40B	5′ CTCTTTCCCTACACGACGCTCTTCCGATCTCTTCAATAAC 3′
8bp-41	5′ AATTGTCGGCAAGAGATCGGAAGAGCGTCGTGTAGGGAAAGAGTGT 3′
8bp-41B	5′ CTCTTTCCCTACACGACGCTCTTCCGATCTCTTGCCGAC 3′
9bp-41	5′ AATTGTTATTCCTTAGATCGGAAGAGCGTCGTGTAGGGAAAGAGTGT 3′
9bp-41B	5′ CTCTTTCCCTACACGACGCTCTTCCGATCTAAGGAATAAC 3′
10bp-150	5′ AATTGTGGTTGAGCGAGATCGGAAGAGCGTCGTGTAGGGAAAGAGTGT 3′
10bp-150B	5′ CTCTTTCCCTACACGACGCTCTTCCGATCTCGCTCAACCAC 3′
8bp-42	5′ AATTGTCGCGGTCAGATCGGAAGAGCGTCGTGTAGGGAAAGAGTGT 3′
8bp-42B	5′ CTCTTTCCCTACACGACGCTCTTCCGATCTGACCGCGAC 3′

Supplementary Table 2 - *Continued from previous page*

**Oligo Name**	**Sequence**
9bp-42	5′ AATTGTTATGAGAAAGATCGGAAGAGCGTCGTGTAGGGAAAGAGTGT 3′
9bp-42B	5′ CTCTTTCCCTACACGACGCTCTTCCGATCTTTCTCATAAC 3′
10bp-151	5′ AATTGTGGTTCTTATAGATCGGAAGAGCGTCGTGTAGGGAAAGAGTGT 3′
10bp-151B	5′ CTCTTTCCCTACACGACGCTCTTCCGATCTATAAGAACCAC 3′
8bp-43	5′ AATTGTCGAACGTAGATCGGAAGAGCGTCGTGTAGGGAAAGAGTGT 3′
8bp-43B	5′ CTCTTTCCCTACACGACGCTCTTCCGATCTACGTTCGAC 3′
9bp-43	5′ AATTGTTATCTATCAGATCGGAAGAGCGTCGTGTAGGGAAAGAGTGT 3′
9bp-43B	5′ CTCTTTCCCTACACGACGCTCTTCCGATCTGATAGATAAC 3′
10bp-152	5′ AATTGTGGTTCGAGGAGATCGGAAGAGCGTCGTGTAGGGAAAGAGTGT 3′
10bp-152B	5′ CTCTTTCCCTACACGACGCTCTTCCGATCTCCTCGAACCAC 3′
8bp-44	5′ AATTGTCCTTATAAGATCGGAAGAGCGTCGTGTAGGGAAAGAGTGT 3′
8bp-44B	5′ CTCTTTCCCTACACGACGCTCTTCCGATCTTATAAGGAC 3′
9bp-44	5′ AATTGTTATCCTGGAGATCGGAAGAGCGTCGTGTAGGGAAAGAGTGT 3′
9bp-44B	5′ CTCTTTCCCTACACGACGCTCTTCCGATCTCCAGGATAAC 3′
10bp-153	5′ AATTGTGGTTAGGACAGATCGGAAGAGCGTCGTGTAGGGAAAGAGTGT 3′
10bp-153B	5′ CTCTTTCCCTACACGACGCTCTTCCGATCTGTCCTAACCAC 3′
8bp-45	5′ AATTGTCCATCAGAGATCGGAAGAGCGTCGTGTAGGGAAAGAGTGT 3′
8bp-45B	5′ CTCTTTCCCTACACGACGCTCTTCCGATCTCTGATGGAC 3′
9bp-45	5′ AATTGTTATAGCGCAGATCGGAAGAGCGTCGTGTAGGGAAAGAGTGT 3′
9bp-45B	5′ CTCTTTCCCTACACGACGCTCTTCCGATCTGCGCTATAAC 3′
10bp-155	5′ AATTGTGGTCTGCAGAGATCGGAAGAGCGTCGTGTAGGGAAAGAGTGT 3′
10bp-155B	5′ CTCTTTCCCTACACGACGCTCTTCCGATCTCTGCAGACCAC 3′
8bp-46	5′ AATTGTCAGTCCAAGATCGGAAGAGCGTCGTGTAGGGAAAGAGTGT 3′
8bp-46B	5′ CTCTTTCCCTACACGACGCTCTTCCGATCTTGGACTGAC 3′
9bp-46	5′ AATTGTTATAATCTAGATCGGAAGAGCGTCGTGTAGGGAAAGAGTGT 3′
9bp-46B	5′ CTCTTTCCCTACACGACGCTCTTCCGATCTAGATTATAAC 3′
10bp-162	5′ AATTGTGGTAATAAGAGATCGGAAGAGCGTCGTGTAGGGAAAGAGTGT 3′
10bp-162B	5′ CTCTTTCCCTACACGACGCTCTTCCGATCTCTTATTACCAC 3′
8bp-47	5′ AATTGTATTCAGTAGATCGGAAGAGCGTCGTGTAGGGAAAGAGTGT 3′
8bp-47B	5′ CTCTTTCCCTACACGACGCTCTTCCGATCTACTGAATAC 3′
9bp-47	5′ AATTGTTAGGTTACAGATCGGAAGAGCGTCGTGTAGGGAAAGAGTGT 3′
9bp-47B	5′ CTCTTTCCCTACACGACGCTCTTCCGATCTGTAACCTAAC 3′
10bp-164	5′ AATTGTGGTAACGGTAGATCGGAAGAGCGTCGTGTAGGGAAAGAGTGT 3′
10bp-164B	5′ CTCTTTCCCTACACGACGCTCTTCCGATCTACCGTTACCAC 3′
8bp-48	5′ AATTGTATTACTAAGATCGGAAGAGCGTCGTGTAGGGAAAGAGTGT 3′
8bp-48B	5′ CTCTTTCCCTACACGACGCTCTTCCGATCTTAGTAATAC 3′
9bp-48	5′ AATTGTTAGGACTGAGATCGGAAGAGCGTCGTGTAGGGAAAGAGTGT 3′
9bp-48B	5′ CTCTTTCCCTACACGACGCTCTTCCGATCTCAGTCCTAAC 3′
10bp-165	5′ AATTGTGGCTCTCTGAGATCGGAAGAGCGTCGTGTAGGGAAAGAGTGT 3′
10bp-165B	5′ CTCTTTCCCTACACGACGCTCTTCCGATCTCAGAGAGCCAC 3′
8bp-49	5′ AATTGTATGCTACAGATCGGAAGAGCGTCGTGTAGGGAAAGAGTGT 3′
8bp-49B	5′ CTCTTTCCCTACACGACGCTCTTCCGATCTGTAGCATAC 3′
9bp-49	5′ AATTGTTACTAGGTAGATCGGAAGAGCGTCGTGTAGGGAAAGAGTGT 3′
9bp-49B	5′ CTCTTTCCCTACACGACGCTCTTCCGATCTACCTAGTAAC 3′
10bp-167	5′ AATTGTGGCGTAATGAGATCGGAAGAGCGTCGTGTAGGGAAAGAGTGT 3′
10bp-167B	5′ CTCTTTCCCTACACGACGCTCTTCCGATCTCATTACGCCAC 3′
8bp-50	5′ AATTGTATCGGATAGATCGGAAGAGCGTCGTGTAGGGAAAGAGTGT 3′
8bp-50B	5′ CTCTTTCCCTACACGACGCTCTTCCGATCTATCCGATAC 3′
9bp-50	5′ AATTGTTACGTCCAAGATCGGAAGAGCGTCGTGTAGGGAAAGAGTGT 3′
9bp-50B	5′ CTCTTTCCCTACACGACGCTCTTCCGATCTTGGACGTAAC 3′
10bp-173	5′ AATTGTGGCCGATAGAGATCGGAAGAGCGTCGTGTAGGGAAAGAGTGT 3′
10bp-173B	5′ CTCTTTCCCTACACGACGCTCTTCCGATCTCTATCGGCCAC 3′
8bp-51	5′ AATTGTATAAGGCAGATCGGAAGAGCGTCGTGTAGGGAAAGAGTGT 3′
8bp-51B	5′ CTCTTTCCCTACACGACGCTCTTCCGATCTGCCTTATAC 3′
9bp-51	5′ AATTGTTACGCAGCAGATCGGAAGAGCGTCGTGTAGGGAAAGAGTGT 3′
9bp-51B	5′ CTCTTTCCCTACACGACGCTCTTCCGATCTGCTGCGTAAC 3′
10bp-176	5′ AATTGTGGCAGCTCAAGATCGGAAGAGCGTCGTGTAGGGAAAGAGTGT 3′
10bp-176B	5′ CTCTTTCCCTACACGACGCTCTTCCGATCTTGAGCTGCCAC 3′
8bp-52	5′ AATTGTAGCAGCAAGATCGGAAGAGCGTCGTGTAGGGAAAGAGTGT 3′
8bp-52B	5′ CTCTTTCCCTACACGACGCTCTTCCGATCTTGCTGCTAC 3′
9bp-52	5′ AATTGTTACCTGAGAGATCGGAAGAGCGTCGTGTAGGGAAAGAGTGT 3′
9bp-52B	5′ CTCTTTCCCTACACGACGCTCTTCCGATCTCTCAGGTAAC 3′

Supplementary Table 2 - *Continued from previous page*

Oligo Name	Sequence
10bp-185	5′ AATTGTGGACGAGGTAGATCGGAAGAGCGTCGTGTAGGGAAAGAGTGT 3′
10bp-185B	5′ CTCTTTCCCTACACGACGCTCTTCCGATCTACCTCGTCCAC 3′
8bp-53	5′ AATTGTAGACTGGAGATCGGAAGAGCGTCGTGTAGGGAAAGAGTGT 3′
8bp-53B	5′ CTCTTTCCCTACACGACGCTCTTCCGATCTCCAGTCTAC 3′
9bp-53	5′ AATTGTTACCGTTAAGATCGGAAGAGCGTCGTGTAGGGAAAGAGTGT 3′
9bp-53B	5′ CTCTTTCCCTACACGACGCTCTTCCGATCTTAACGGTAAC 3′
10bp-188	5′ AATTGTGCTTCCTCGAGATCGGAAGAGCGTCGTGTAGGGAAAGAGTGT 3′
10bp-188B	5′ CTCTTTCCCTACACGACGCTCTTCCGATCTCGAGGAAGCAC 3′
8bp-54	5′ AATTGTACTATATAGATCGGAAGAGCGTCGTGTAGGGAAAGAGTGT 3′
8bp-54B	5′ CTCTTTCCCTACACGACGCTCTTCCGATCTATATAGTAC 3′
9bp-54	5′ AATTGTTAAGGATTAGATCGGAAGAGCGTCGTGTAGGGAAAGAGTGT 3′
9bp-54B	5′ CTCTTTCCCTACACGACGCTCTTCCGATCTAATCCTTAAC 3′
8bp-55	5′ AATTGTACCTCTTAGATCGGAAGAGCGTCGTGTAGGGAAAGAGTGT 3′
8bp-55B	5′ CTCTTTCCCTACACGACGCTCTTCCGATCTAAGAGGTAC 3′
10bp-191	5′ AATTGTGCTGCGCCTAGATCGGAAGAGCGTCGTGTAGGGAAAGAGTGT 3′
10bp-191B	5′ CTCTTTCCCTACACGACGCTCTTCCGATCTAGGCGCAGCAC 3′
8bp-56	5′ AATTGTAATTGAAAGATCGGAAGAGCGTCGTGTAGGGAAAGAGTGT 3′
8bp-56B	5′ CTCTTTCCCTACACGACGCTCTTCCGATCTTTCAATTAC 3′
10bp-192	5′ AATTGTGCTGAGATGAGATCGGAAGAGCGTCGTGTAGGGAAAGAGTGT 3′
10bp-192B	5′ CTCTTTCCCTACACGACGCTCTTCCGATCTCATCTCAGCAC 3′
8bp-57	5′ AATTGTAACGCGGAGATCGGAAGAGCGTCGTGTAGGGAAAGAGTGT 3′
8bp-57B	5′ CTCTTTCCCTACACGACGCTCTTCCGATCTCCGCGTTAC 3′
9bp-57	5′ AATTGTGGTTAACCAGATCGGAAGAGCGTCGTGTAGGGAAAGAGTGT 3′
9bp-57B	5′ CTCTTTCCCTACACGACGCTCTTCCGATCTGGTTAACCAC 3′
10bp-195	5′ AATTGTGCTCCAATTAGATCGGAAGAGCGTCGTGTAGGGAAAGAGTGT 3′
10bp-195B	5′ CTCTTTCCCTACACGACGCTCTTCCGATCTAATTGGAGCAC 3′
8bp-58	5′ AATTGGTTGGAGGAGATCGGAAGAGCGTCGTGTAGGGAAAGAGTGT 3′
8bp-58B	5′ CTCTTTCCCTACACGACGCTCTTCCGATCTCCTCCAACC 3′
9bp-58	5′ AATTGTGGTCTGACAGATCGGAAGAGCGTCGTGTAGGGAAAGAGTGT 3′
9bp-58B	5′ CTCTTTCCCTACACGACGCTCTTCCGATCTGTCAGACCAC 3′
10bp-200	5′ AATTGTGCGTTCCTGAGATCGGAAGAGCGTCGTGTAGGGAAAGAGTGT 3′
10bp-200B	5′ CTCTTTCCCTACACGACGCTCTTCCGATCTCAGGAACGCAC 3′
8bp-59	5′ AATTGGTTCGCAAAGATCGGAAGAGCGTCGTGTAGGGAAAGAGTGT 3′
8bp-59B	5′ CTCTTTCCCTACACGACGCTCTTCCGATCTTTGCGAACC 3′
9bp-59	5′ AATTGTGGTCGTCTAGATCGGAAGAGCGTCGTGTAGGGAAAGAGTGT 3′
9bp-59B	5′ CTCTTTCCCTACACGACGCTCTTCCGATCTAGACGACCAC 3′
10bp-211	5′ AATTGTGCGAGAGTTAGATCGGAAGAGCGTCGTGTAGGGAAAGAGTGT 3′
10bp-211B	5′ CTCTTTCCCTACACGACGCTCTTCCGATCTAACTCTCGCAC 3′
9bp-60	5′ AATTGTGGTATAGGAGATCGGAAGAGCGTCGTGTAGGGAAAGAGTGT 3′
9bp-60B	5′ CTCTTTCCCTACACGACGCTCTTCCGATCTCCTATACCAC 3′
10bp-213	5′ AATTGTGCCTGCATTAGATCGGAAGAGCGTCGTGTAGGGAAAGAGTGT 3′
10bp-213B	5′ CTCTTTCCCTACACGACGCTCTTCCGATCTAATGCAGGCAC 3′
8bp-61	5′ AATTGGTTCAGGTAGATCGGAAGAGCGTCGTGTAGGGAAAGAGTGT 3′
8bp-61B	5′ CTCTTTCCCTACACGACGCTCTTCCGATCTACCTGAACC 3′
9bp-61	5′ AATTGTGGCTCAAGAGATCGGAAGAGCGTCGTGTAGGGAAAGAGTGT 3′
9bp-61B	5′ CTCTTTCCCTACACGACGCTCTTCCGATCTCTTGAGCCAC 3′
10bp-215	5′ AATTGTGCCGTCCAAAGATCGGAAGAGCGTCGTGTAGGGAAAGAGTGT 3′
10bp-215B	5′ CTCTTTCCCTACACGACGCTCTTCCGATCTTTGGACGGCAC 3′
8bp-62	5′ AATTGGTTATATTAGATCGGAAGAGCGTCGTGTAGGGAAAGAGTGT 3′
8bp-62B	5′ CTCTTTCCCTACACGACGCTCTTCCGATCTAATATAACC 3′
9bp-62	5′ AATTGTGGCGGAGTAGATCGGAAGAGCGTCGTGTAGGGAAAGAGTGT 3′
9bp-62B	5′ CTCTTTCCCTACACGACGCTCTTCCGATCTACTCCGCCAC 3′
10bp-218	5′ AATTGTGCCAATTAAAGATCGGAAGAGCGTCGTGTAGGGAAAGAGTGT 3′
10bp-218B	5′ CTCTTTCCCTACACGACGCTCTTCCGATCTTTAATTGGCAC 3′
8bp-63	5′ AATTGGTTACCGCAGATCGGAAGAGCGTCGTGTAGGGAAAGAGTGT 3′
8bp-63B	5′ CTCTTTCCCTACACGACGCTCTTCCGATCTGCGGTAACC 3′
9bp-63	5′ AATTGTGGCGCTCCAGATCGGAAGAGCGTCGTGTAGGGAAAGAGTGT 3′
9bp-63B	5′ CTCTTTCCCTACACGACGCTCTTCCGATCTGGAGCGCCAC 3′
10bp-221	5′ AATTGTGCAATTATGAGATCGGAAGAGCGTCGTGTAGGGAAAGAGTGT 3′
10bp-221B	5′ CTCTTTCCCTACACGACGCTCTTCCGATCTCATAATTGCAC 3′
8bp-64	5′ AATTGGTCTTAAGAGATCGGAAGAGCGTCGTGTAGGGAAAGAGTGT 3′
8bp-64B	5′ CTCTTTCCCTACACGACGCTCTTCCGATCTCTTAAGACC 3′

Supplementary Table 2 - *Continued from previous page*

**Oligo Name**	**Sequence**
9bp-64	5′ AATTGTGGCCTCCGAGATCGGAAGAGCGTCGTGTAGGGAAAGAGTGT 3′
9bp-64B	5′ CTCTTTCCCTACACGACGCTCTTCCGATCTCGGAGGCCAC 3′
10bp-222	5′ AATTGTGCAAGTTCTAGATCGGAAGAGCGTCGTGTAGGGAAAGAGTGT 3′
10bp-222B	5′ CTCTTTCCCTACACGACGCTCTTCCGATCTAGAACTTGCAC 3′
8bp-65	5′ AATTGGTCTCGTCAGATCGGAAGAGCGTCGTGTAGGGAAAGAGTGT 3′
8bp-65B	5′ CTCTTTCCCTACACGACGCTCTTCCGATCTGACGAGACC 3′
9bp-65	5′ AATTGTGGCATTATAGATCGGAAGAGCGTCGTGTAGGGAAAGAGTGT 3′
9bp-65B	5′ CTCTTTCCCTACACGACGCTCTTCCGATCTATAATGCCAC 3′
10bp-225	5′ AATTGTGATTAATTGAGATCGGAAGAGCGTCGTGTAGGGAAAGAGTGT 3′
10bp-225B	5′ CTCTTTCCCTACACGACGCTCTTCCGATCTCAATTAATCAC 3′
8bp-66	5′ AATTGGTCGTCCTAGATCGGAAGAGCGTCGTGTAGGGAAAGAGTGT 3′
8bp-66B	5′ CTCTTTCCCTACACGACGCTCTTCCGATCTAGGACGACC 3′
9bp-66	5′ AATTGTGGCAACGCAGATCGGAAGAGCGTCGTGTAGGGAAAGAGTGT 3′
9bp-66B	5′ CTCTTTCCCTACACGACGCTCTTCCGATCTGCGTTGCCAC 3′
10bp-227	5′ AATTGTGATGCATAAAGATCGGAAGAGCGTCGTGTAGGGAAAGAGTGT 3′
10bp-227B	5′ CTCTTTCCCTACACGACGCTCTTCCGATCTTTATGCATCAC 3′
8bp-67	5′ AATTGGTCCAATAAGATCGGAAGAGCGTCGTGTAGGGAAAGAGTGT 3′
8bp-67B	5′ CTCTTTCCCTACACGACGCTCTTCCGATCTTATTGGACC 3′
9bp-67	5′ AATTGTGGAGTCTAAGATCGGAAGAGCGTCGTGTAGGGAAAGAGTGT 3′
9bp-67B	5′ CTCTTTCCCTACACGACGCTCTTCCGATCTTAGACTCCAC 3′
10bp-231	5′ AATTGTGATAGAATAAGATCGGAAGAGCGTCGTGTAGGGAAAGAGTGT 3′
10bp-231B	5′ CTCTTTCCCTACACGACGCTCTTCCGATCTTATTCTATCAC 3′
8bp-68	5′ AATTGGTCAAGCGAGATCGGAAGAGCGTCGTGTAGGGAAAGAGTGT 3′
8bp-68B	5′ CTCTTTCCCTACACGACGCTCTTCCGATCTCGCTTGACC 3′
9bp-68	5′ AATTGTGGACGATCAGATCGGAAGAGCGTCGTGTAGGGAAAGAGTGT 3′
9bp-68B	5′ CTCTTTCCCTACACGACGCTCTTCCGATCTGATCGTCCAC 3′
10bp-239	5′ AATTGTGAGGCAAGCAGATCGGAAGAGCGTCGTGTAGGGAAAGAGTGT 3′
10bp-239B	5′ CTCTTTCCCTACACGACGCTCTTCCGATCTGCTTGCCTCAC 3′
8bp-69	5′ AATTGGTAGCCTAAGATCGGAAGAGCGTCGTGTAGGGAAAGAGTGT 3′
8bp-69B	5′ CTCTTTCCCTACACGACGCTCTTCCGATCTTAGGCTACC 3′
9bp-69	5′ AATTGTGGAAGGAGAGATCGGAAGAGCGTCGTGTAGGGAAAGAGTGT 3′
9bp-69B	5′ CTCTTTCCCTACACGACGCTCTTCCGATCTCTCCTTCCAC 3′
10bp-243	5′ AATTGTGAGCATGCTAGATCGGAAGAGCGTCGTGTAGGGAAAGAGTGT 3′
10bp-243B	5′ CTCTTTCCCTACACGACGCTCTTCCGATCTAGCATGCTCAC 3′
8bp-70	5′ AATTGGTAATGACAGATCGGAAGAGCGTCGTGTAGGGAAAGAGTGT 3′
8bp-70B	5′ CTCTTTCCCTACACGACGCTCTTCCGATCTGTCATTACC 3′
10bp-244	5′ AATTGTGAGATTAGTAGATCGGAAGAGCGTCGTGTAGGGAAAGAGTGT 3′
10bp-244B	5′ CTCTTTCCCTACACGACGCTCTTCCGATCTACTAATCTCAC 3′
8bp-71	5′ AATTGGTAAGTTGAGATCGGAAGAGCGTCGTGTAGGGAAAGAGTGT 3′
8bp-71B	5′ CTCTTTCCCTACACGACGCTCTTCCGATCTCAACTTACC 3′
9bp-71	5′ AATTGTGCTGAGTTAGATCGGAAGAGCGTCGTGTAGGGAAAGAGTGT 3′
9bp-71B	5′ CTCTTTCCCTACACGACGCTCTTCCGATCTAACTCAGCAC 3′
10bp-249	5′ AATTGTGACGCGAATAGATCGGAAGAGCGTCGTGTAGGGAAAGAGTGT 3′
10bp-249B	5′ CTCTTTCCCTACACGACGCTCTTCCGATCTATTCGCGTCAC 3′
8bp-72	5′ AATTGGGTTGATCAGATCGGAAGAGCGTCGTGTAGGGAAAGAGTGT 3′
8bp-72B	5′ CTCTTTCCCTACACGACGCTCTTCCGATCTGATCAACCC 3′
9bp-72	5′ AATTGTGCTCCTTCAGATCGGAAGAGCGTCGTGTAGGGAAAGAGTGT 3′
9bp-72B	5′ CTCTTTCCCTACACGACGCTCTTCCGATCTGAAGGAGCAC 3′
10bp-251	5′ AATTGTGACCTTGGAAGATCGGAAGAGCGTCGTGTAGGGAAAGAGTGT 3′
10bp-251B	5′ CTCTTTCCCTACACGACGCTCTTCCGATCTTCCAAGGTCAC 3′
8bp-73	5′ AATTGGGTCTTGCAGATCGGAAGAGCGTCGTGTAGGGAAAGAGTGT 3′
8bp-73B	5′ CTCTTTCCCTACACGACGCTCTTCCGATCTGCAAGACCC 3′
9bp-73	5′ AATTGTGCTACCATAGATCGGAAGAGCGTCGTGTAGGGAAAGAGTGT 3′
9bp-73B	5′ CTCTTTCCCTACACGACGCTCTTCCGATCTATGGTAGCAC 3′
10bp-253	5′ AATTGTGACCATATGAGATCGGAAGAGCGTCGTGTAGGGAAAGAGTGT 3′
10bp-253B	5′ CTCTTTCCCTACACGACGCTCTTCCGATCTCATATGGTCAC 3′
8bp-74	5′ AATTGGGCTGGCTAGATCGGAAGAGCGTCGTGTAGGGAAAGAGTGT 3′
8bp-74B	5′ CTCTTTCCCTACACGACGCTCTTCCGATCTAGCCAGCCC 3′
9bp-74	5′ AATTGTGCGTTACGAGATCGGAAGAGCGTCGTGTAGGGAAAGAGTGT 3′
9bp-74B	5′ CTCTTTCCCTACACGACGCTCTTCCGATCTCGTAACGCAC 3′
10bp-256	5′ AATTGTGAATACGCCAGATCGGAAGAGCGTCGTGTAGGGAAAGAGTGT 3′
10bp-256B	5′ CTCTTTCCCTACACGACGCTCTTCCGATCTGGCGTATTCAC 3′

Supplementary Table 2 - *Continued from previous page*

**Oligo Name**	**Sequence**
8bp-75	5′ AATTGGGCTCTGGAGATCGGAAGAGCGTCGTGTAGGGAAAGAGTGT 3′
8bp-75B	5′ CTCTTTCCCTACACGACGCTCTTCCGATCTCCAGAGCCC 3′
9bp-75	5′ AATTGTGCGTAGACAGATCGGAAGAGCGTCGTGTAGGGAAAGAGTGT 3′
9bp-75B	5′ CTCTTTCCCTACACGACGCTCTTCCGATCTGTCTACGCAC 3′
10bp-260	5′ AATTGTCTTGGTTCGAGATCGGAAGAGCGTCGTGTAGGGAAAGAGTGT 3′
10bp-260B	5′ CTCTTTCCCTACACGACGCTCTTCCGATCTCGAACCAAGAC 3′
8bp-76	5′ AATTGGGCGATAAAGATCGGAAGAGCGTCGTGTAGGGAAAGAGTGT 3′
8bp-76B	5′ CTCTTTCCCTACACGACGCTCTTCCGATCTTTATCGCCC 3′
9bp-76	5′ AATTGTGCGGTCGTAGATCGGAAGAGCGTCGTGTAGGGAAAGAGTGT 3′
9bp-76B	5′ CTCTTTCCCTACACGACGCTCTTCCGATCTACGACCGCAC 3′
8bp-77	5′ AATTGGGCATCGAAGATCGGAAGAGCGTCGTGTAGGGAAAGAGTGT 3′
8bp-77B	5′ CTCTTTCCCTACACGACGCTCTTCCGATCTTCGATGCCC 3′
9bp-77	5′ AATTGTGCGAGGCTAGATCGGAAGAGCGTCGTGTAGGGAAAGAGTGT 3′
9bp-77B	5′ CTCTTTCCCTACACGACGCTCTTCCGATCTAGCCTCGCAC 3′
10bp-262	5′ AATTGTCTTGGACGTAGATCGGAAGAGCGTCGTGTAGGGAAAGAGTGT 3′
10bp-262B	5′ CTCTTTCCCTACACGACGCTCTTCCGATCTACGTCCAAGAC 3′
8bp-78	5′ AATTGGGATACTGAGATCGGAAGAGCGTCGTGTAGGGAAAGAGTGT 3′
8bp-78B	5′ CTCTTTCCCTACACGACGCTCTTCCGATCTCAGTATCCC 3′
9bp-78	5′ AATTGTGCGACTGGAGATCGGAAGAGCGTCGTGTAGGGAAAGAGTGT 3′
9bp-78B	5′ CTCTTTCCCTACACGACGCTCTTCCGATCTCCAGTCGCAC 3′
8bp-79	5′ AATTGGGAGTAGTAGATCGGAAGAGCGTCGTGTAGGGAAAGAGTGT 3′
8bp-79B	5′ CTCTTTCCCTACACGACGCTCTTCCGATCTACTACTCCC 3′
9bp-79	5′ AATTGTGCCTGCCAAGATCGGAAGAGCGTCGTGTAGGGAAAGAGTGT 3′
9bp-79B	5′ CTCTTTCCCTACACGACGCTCTTCCGATCTTGGCAGGCAC 3′
10bp-264	5′ AATTGTCTTGAGTATAGATCGGAAGAGCGTCGTGTAGGGAAAGAGTGT 3′
10bp-264B	5′ CTCTTTCCCTACACGACGCTCTTCCGATCTATACTCAAGAC 3′
8bp-80	5′ AATTGGGAGGTCCAGATCGGAAGAGCGTCGTGTAGGGAAAGAGTGT 3′
8bp-80B	5′ CTCTTTCCCTACACGACGCTCTTCCGATCTGGACCTCCC 3′
9bp-80	5′ AATTGTGCCGTATCAGATCGGAAGAGCGTCGTGTAGGGAAAGAGTGT 3′
9bp-80B	5′ CTCTTTCCCTACACGACGCTCTTCCGATCTGATACGGCAC 3′
10bp-265	5′ AATTGTCTTCTTCTGAGATCGGAAGAGCGTCGTGTAGGGAAAGAGTGT 3′
10bp-265B	5′ CTCTTTCCCTACACGACGCTCTTCCGATCTCAGAAGAAGAC 3′
8bp-81	5′ AATTGGGACCGAGAGATCGGAAGAGCGTCGTGTAGGGAAAGAGTGT 3′
8bp-81B	5′ CTCTTTCCCTACACGACGCTCTTCCGATCTCTCGGTCCC 3′
9bp-81	5′ AATTGTGCAGCGGAAGATCGGAAGAGCGTCGTGTAGGGAAAGAGTGT 3′
9bp-81B	5′ CTCTTTCCCTACACGACGCTCTTCCGATCTTCCGCTGCAC 3′
10bp-274	5′ AATTGTCTTAATCCTAGATCGGAAGAGCGTCGTGTAGGGAAAGAGTGT 3′
10bp-274B	5′ CTCTTTCCCTACACGACGCTCTTCCGATCTAGGATTAAGAC 3′
8bp-82	5′ AATTGGCTGCGCCAGATCGGAAGAGCGTCGTGTAGGGAAAGAGTGT 3′
8bp-82B	5′ CTCTTTCCCTACACGACGCTCTTCCGATCTGGCGCAGCC 3′
9bp-82	5′ AATTGTGCAGACCGAGATCGGAAGAGCGTCGTGTAGGGAAAGAGTGT 3′
9bp-82B	5′ CTCTTTCCCTACACGACGCTCTTCCGATCTCGGTCTGCAC 3′
10bp-290	5′ AATTGTCTCTTATCTAGATCGGAAGAGCGTCGTGTAGGGAAAGAGTGT 3′
10bp-290B	5′ CTCTTTCCCTACACGACGCTCTTCCGATCTAGATAAGAGAC 3′
8bp-83	5′ AATTGGCTAATGGAGATCGGAAGAGCGTCGTGTAGGGAAAGAGTGT 3′
8bp-83B	5′ CTCTTTCCCTACACGACGCTCTTCCGATCTCCATTAGCC 3′
9bp-83	5′ AATTGTGATGCGCGAGATCGGAAGAGCGTCGTGTAGGGAAAGAGTGT 3′
9bp-83B	5′ CTCTTTCCCTACACGACGCTCTTCCGATCTCGCGCATCAC 3′
10bp-302	5′ AATTGTCTCCGTATAAGATCGGAAGAGCGTCGTGTAGGGAAAGAGTGT 3′
10bp-302B	5′ CTCTTTCCCTACACGACGCTCTTCCGATCTTATACGGAGAC 3′
8bp-84	5′ AATTGGCGTTGCGAGATCGGAAGAGCGTCGTGTAGGGAAAGAGTGT 3′
8bp-84B	5′ CTCTTTCCCTACACGACGCTCTTCCGATCTCGCAACGCC 3′
9bp-84	5′ AATTGTGATCGCAAAGATCGGAAGAGCGTCGTGTAGGGAAAGAGTGT 3′
9bp-84B	5′ CTCTTTCCCTACACGACGCTCTTCCGATCTTTGCGATCAC 3′
10bp-306	5′ AATTGTCTATTCGCGAGATCGGAAGAGCGTCGTGTAGGGAAAGAGTGT 3′
10bp-306B	5′ CTCTTTCCCTACACGACGCTCTTCCGATCTCGCGAATAGAC 3′
8bp-85	5′ AATTGGCGTCTATAGATCGGAAGAGCGTCGTGTAGGGAAAGAGTGT 3′
8bp-85B	5′ CTCTTTCCCTACACGACGCTCTTCCGATCTATAGACGCC 3′
9bp-85	5′ AATTGTGATCAAGTAGATCGGAAGAGCGTCGTGTAGGGAAAGAGTGT 3′
9bp-85B	5′ CTCTTTCCCTACACGACGCTCTTCCGATCTACTTGATCAC 3′
10bp-312	5′ AATTGTCTAGCTGAAAGATCGGAAGAGCGTCGTGTAGGGAAAGAGTGT 3′
10bp-312B	5′ CTCTTTCCCTACACGACGCTCTTCCGATCTTTCAGCTAGAC 3′

Supplementary Table 2 - *Continued from previous page*

**Oligo Name**	**Sequence**
8bp-86	5′ AATTGGCGTAAGCAGATCGGAAGAGCGTCGTGTAGGGAAAGAGTGT 3′
8bp-86B	5′ CTCTTTCCCTACACGACGCTCTTCCGATCTGCTTACGCC 3′
9bp-86	5′ AATTGTGAGTCTCTAGATCGGAAGAGCGTCGTGTAGGGAAAGAGTGT 3′
9bp-86B	5′ CTCTTTCCCTACACGACGCTCTTCCGATCTAGAGACTCAC 3′
10bp-320	5′ AATTGTCGTCGTTGAAGATCGGAAGAGCGTCGTGTAGGGAAAGAGTGT 3′
10bp-320B	5′ CTCTTTCCCTACACGACGCTCTTCCGATCTTCAACGACGAC 3′
8bp-87	5′ AATTGGCGGTCTCAGATCGGAAGAGCGTCGTGTAGGGAAAGAGTGT 3′
8bp-87B	5′ CTCTTTCCCTACACGACGCTCTTCCGATCTGAGACCGCC 3′
9bp-87	5′ AATTGTGAGCCGGCAGATCGGAAGAGCGTCGTGTAGGGAAAGAGTGT 3′
9bp-87B	5′ CTCTTTCCCTACACGACGCTCTTCCGATCTGCCGGCTCAC 3′
10bp-336	5′ AATTGTCGATCAGAGAGATCGGAAGAGCGTCGTGTAGGGAAAGAGTGT 3′
10bp-336B	5′ CTCTTTCCCTACACGACGCTCTTCCGATCTCTCTGATCGAC 3′
8bp-88	5′ AATTGGCCGGAACAGATCGGAAGAGCGTCGTGTAGGGAAAGAGTGT 3′
8bp-88B	5′ CTCTTTCCCTACACGACGCTCTTCCGATCTGTTCCGGCC 3′
9bp-88	5′ AATTGTGAGCATAGAGATCGGAAGAGCGTCGTGTAGGGAAAGAGTGT 3′
9bp-88B	5′ CTCTTTCCCTACACGACGCTCTTCCGATCTCTATGCTCAC 3′
10bp-340	5′ AATTGTCCTTCCAATAGATCGGAAGAGCGTCGTGTAGGGAAAGAGTGT 3′
10bp-340B	5′ CTCTTTCCCTACACGACGCTCTTCCGATCTATTGGAAGGAC 3′
8bp-89	5′ AATTGGCCGAGTTAGATCGGAAGAGCGTCGTGTAGGGAAAGAGTGT 3′
8bp-89B	5′ CTCTTTCCCTACACGACGCTCTTCCGATCTAACTCGGCC 3′
9bp-89	5′ AATTGTGAGATCTCAGATCGGAAGAGCGTCGTGTAGGGAAAGAGTGT 3′
9bp-89B	5′ CTCTTTCCCTACACGACGCTCTTCCGATCTGAGATCTCAC 3′
10bp-345	5′ AATTGTCCTAACCTGAGATCGGAAGAGCGTCGTGTAGGGAAAGAGTGT 3′
10bp-345B	5′ CTCTTTCCCTACACGACGCTCTTCCGATCTCAGGTTAGGAC 3′
8bp-90	5′ AATTGGCAGTTAGAGATCGGAAGAGCGTCGTGTAGGGAAAGAGTGT 3′
8bp-90B	5′ CTCTTTCCCTACACGACGCTCTTCCGATCTCTAACTGCC 3′
9bp-90	5′ AATTGTGACTTGCCAGATCGGAAGAGCGTCGTGTAGGGAAAGAGTGT 3′
9bp-90B	5′ CTCTTTCCCTACACGACGCTCTTCCGATCTGGCAAGTCAC 3′
8bp-91	5′ AATTGGCAAGCATAGATCGGAAGAGCGTCGTGTAGGGAAAGAGTGT 3′
8bp-91B	5′ CTCTTTCCCTACACGACGCTCTTCCGATCTATGCTTGCC 3′
9bp-91	5′ AATTGTGAATTCGGAGATCGGAAGAGCGTCGTGTAGGGAAAGAGTGT 3′
9bp-91B	5′ CTCTTTCCCTACACGACGCTCTTCCGATCTCCGAATTCAC 3′
10bp-355	5′ AATTGTCCAGCATATAGATCGGAAGAGCGTCGTGTAGGGAAAGAGTGT 3′
10bp-355B	5′ CTCTTTCCCTACACGACGCTCTTCCGATCTATATGCTGGAC 3′
8bp-92	5′ AATTGGATTGCCGAGATCGGAAGAGCGTCGTGTAGGGAAAGAGTGT 3′
8bp-92B	5′ CTCTTTCCCTACACGACGCTCTTCCGATCTCGGCAATCC 3′
9bp-92	5′ AATTGTGAATATTCAGATCGGAAGAGCGTCGTGTAGGGAAAGAGTGT 3′
9bp-92B	5′ CTCTTTCCCTACACGACGCTCTTCCGATCTGAATATTCAC 3′
10bp-356	5′ AATTGTCCAGATTCCAGATCGGAAGAGCGTCGTGTAGGGAAAGAGTGT 3′
10bp-356B	5′ CTCTTTCCCTACACGACGCTCTTCCGATCTGGAATCTGGAC 3′
8bp-93	5′ AATTGGATGAGAGAGATCGGAAGAGCGTCGTGTAGGGAAAGAGTGT 3′
8bp-93B	5′ CTCTTTCCCTACACGACGCTCTTCCGATCTCTCTCATCC 3′
10bp-357	5′ AATTGTCCAATGACTAGATCGGAAGAGCGTCGTGTAGGGAAAGAGTGT 3′
10bp-357B	5′ CTCTTTCCCTACACGACGCTCTTCCGATCTAGTCATTGGAC 3′
8bp-94	5′ AATTGGATCAACCAGATCGGAAGAGCGTCGTGTAGGGAAAGAGTGT 3′
8bp-94B	5′ CTCTTTCCCTACACGACGCTCTTCCGATCTGGTTGATCC 3′
10bp-363	5′ AATTGTCATATGCTAAGATCGGAAGAGCGTCGTGTAGGGAAAGAGTGT 3′
10bp-363B	5′ CTCTTTCCCTACACGACGCTCTTCCGATCTTAGCATATGAC 3′
8bp-95	5′ AATTGGAGTCATGAGATCGGAAGAGCGTCGTGTAGGGAAAGAGTGT 3′
8bp-95B	5′ CTCTTTCCCTACACGACGCTCTTCCGATCTCATGACTCC 3′
9bp-95	5′ AATTGTCTTGAGCCAGATCGGAAGAGCGTCGTGTAGGGAAAGAGTGT 3′
9bp-95B	5′ CTCTTTCCCTACACGACGCTCTTCCGATCTGGCTCAAGAC 3′
10bp-370	5′ AATTGTCAGATATAAAGATCGGAAGAGCGTCGTGTAGGGAAAGAGTGT 3′
10bp-370B	5′ CTCTTTCCCTACACGACGCTCTTCCGATCTTTATATCTGAC 3′
8bp-96	5′ AATTGGAGGCCGTAGATCGGAAGAGCGTCGTGTAGGGAAAGAGTGT 3′
8bp-96B	5′ CTCTTTCCCTACACGACGCTCTTCCGATCTACGGCCTCC 3′
10bp-371	5′ AATTGTCAGAACTGGAGATCGGAAGAGCGTCGTGTAGGGAAAGAGTGT 3′
10bp-371B	5′ CTCTTTCCCTACACGACGCTCTTCCGATCTCCAGTTCTGAC 3′
8bp-97	5′ AATTGGAGCTAATAGATCGGAAGAGCGTCGTGTAGGGAAAGAGTGT 3′
8bp-97B	5′ CTCTTTCCCTACACGACGCTCTTCCGATCTATTAGCTCC 3′
9bp-97	5′ AATTGTCTTCGATTAGATCGGAAGAGCGTCGTGTAGGGAAAGAGTGT 3′
9bp-97B	5′ CTCTTTCCCTACACGACGCTCTTCCGATCTAATCGAAGAC 3′

Supplementary Table 2 - *Continued from previous page*

**Oligo Name**	**Sequence**
10bp-373	5′ AATTGTCAAGGACCGAGATCGGAAGAGCGTCGTGTAGGGAAAGAGTGT 3′
10bp-373B	5′ CTCTTTCCCTACACGACGCTCTTCCGATCTCGGTCCTTGAC 3′
8bp-98	5′ AATTGGAGAGTCAAGATCGGAAGAGCGTCGTGTAGGGAAAGAGTGT 3′
8bp-98B	5′ CTCTTTCCCTACACGACGCTCTTCCGATCTTGACTCTCC 3′
9bp-98	5′ AATTGTCTTATGAGAGATCGGAAGAGCGTCGTGTAGGGAAAGAGTGT 3′
9bp-98B	5′ CTCTTTCCCTACACGACGCTCTTCCGATCTCTCATAAGAC 3′
10bp-376	5′ AATTGTCAACGGCGTAGATCGGAAGAGCGTCGTGTAGGGAAAGAGTGT 3′
10bp-376B	5′ CTCTTTCCCTACACGACGCTCTTCCGATCTACGCCGTTGAC 3′
8bp-99	5′ AATTGGAATTCGCAGATCGGAAGAGCGTCGTGTAGGGAAAGAGTGT 3′
8bp-99B	5′ CTCTTTCCCTACACGACGCTCTTCCGATCTGCGAATTCC 3′
9bp-99	5′ AATTGTCTTACCTCAGATCGGAAGAGCGTCGTGTAGGGAAAGAGTGT 3′
9bp-99B	5′ CTCTTTCCCTACACGACGCTCTTCCGATCTGAGGTAAGAC 3′
10bp-379	5′ AATTGTATTGCCGCAAGATCGGAAGAGCGTCGTGTAGGGAAAGAGTGT 3′
10bp-379B	5′ CTCTTTCCCTACACGACGCTCTTCCGATCTTGCGGCAATAC 3′
8bp-100	5′ AATTGCTTGAATCAGATCGGAAGAGCGTCGTGTAGGGAAAGAGTGT 3′
8bp-100B	5′ CTCTTTCCCTACACGACGCTCTTCCGATCTGATTCAAGC 3′
9bp-100	5′ AATTGTCTGGTTCGAGATCGGAAGAGCGTCGTGTAGGGAAAGAGTGT 3′
9bp-100B	5′ CTCTTTCCCTACACGACGCTCTTCCGATCTCGAACCAGAC 3′
10bp-402	5′ AATTGTATCGTCGGCAGATCGGAAGAGCGTCGTGTAGGGAAAGAGTGT 3′
10bp-402B	5′ CTCTTTCCCTACACGACGCTCTTCCGATCTGCCGACGATAC 3′
8bp-101	5′ AATTGCTTATTAGAGATCGGAAGAGCGTCGTGTAGGGAAAGAGTGT 3′
8bp-101B	5′ CTCTTTCCCTACACGACGCTCTTCCGATCTCTAATAAGC 3′
9bp-101	5′ AATTGTCTGCGGACAGATCGGAAGAGCGTCGTGTAGGGAAAGAGTGT 3′
9bp-101B	5′ CTCTTTCCCTACACGACGCTCTTCCGATCTGTCCGCAGAC 3′
10bp-412	5′ AATTGTATAGTTATAAGATCGGAAGAGCGTCGTGTAGGGAAAGAGTGT 3′
10bp-412B	5′ CTCTTTCCCTACACGACGCTCTTCCGATCTTATAACTATAC 3′
8bp-102	5′ AATTGCTGCTCGTAGATCGGAAGAGCGTCGTGTAGGGAAAGAGTGT 3′
8bp-102B	5′ CTCTTTCCCTACACGACGCTCTTCCGATCTACGAGCAGC 3′
9bp-102	5′ AATTGTCTGCCAGGAGATCGGAAGAGCGTCGTGTAGGGAAAGAGTGT 3′
9bp-102B	5′ CTCTTTCCCTACACGACGCTCTTCCGATCTCCTGGCAGAC 3′
10bp-420	5′ AATTGTAGTAGGCGGAGATCGGAAGAGCGTCGTGTAGGGAAAGAGTGT 3′
10bp-420B	5′ CTCTTTCCCTACACGACGCTCTTCCGATCTCCGCCTACTAC 3′
8bp-103	5′ AATTGCTGCCGCCAGATCGGAAGAGCGTCGTGTAGGGAAAGAGTGT 3′
8bp-103B	5′ CTCTTTCCCTACACGACGCTCTTCCGATCTGGCGGCAGC 3′
9bp-103	5′ AATTGTCTGAAGTTAGATCGGAAGAGCGTCGTGTAGGGAAAGAGTGT 3′
9bp-103B	5′ CTCTTTCCCTACACGACGCTCTTCCGATCTAACTTCAGAC 3′
10bp-422	5′ AATTGTAGGTACTCAAGATCGGAAGAGCGTCGTGTAGGGAAAGAGTGT 3′
10bp-422B	5′ CTCTTTCCCTACACGACGCTCTTCCGATCTTGAGTACCTAC 3′
8bp-104	5′ AATTGCTCGTAGAAGATCGGAAGAGCGTCGTGTAGGGAAAGAGTGT 3′
8bp-104B	5′ CTCTTTCCCTACACGACGCTCTTCCGATCTTCTACGAGC 3′
9bp-104	5′ AATTGTCTCTTCGTAGATCGGAAGAGCGTCGTGTAGGGAAAGAGTGT 3′
9bp-104B	5′ CTCTTTCCCTACACGACGCTCTTCCGATCTACGAAGAGAC 3′
10bp-423	5′ AATTGTAGGCCTTAGAGATCGGAAGAGCGTCGTGTAGGGAAAGAGTGT 3′
10bp-423B	5′ CTCTTTCCCTACACGACGCTCTTCCGATCTCTAAGGCCTAC 3′
9bp-105	5′ AATTGTCTCTGACCAGATCGGAAGAGCGTCGTGTAGGGAAAGAGTGT 3′
9bp-105B	5′ CTCTTTCCCTACACGACGCTCTTCCGATCTGGTCAGAGAC 3′
10bp-426	5′ AATTGTAGCTATAGGAGATCGGAAGAGCGTCGTGTAGGGAAAGAGTGT 3′
10bp-426B	5′ CTCTTTCCCTACACGACGCTCTTCCGATCTCCTATAGCTAC 3′
8bp-106	5′ AATTGCTCCGGTTAGATCGGAAGAGCGTCGTGTAGGGAAAGAGTGT 3′
8bp-106B	5′ CTCTTTCCCTACACGACGCTCTTCCGATCTAACCGGAGC 3′
9bp-106	5′ AATTGTCTCCATTGAGATCGGAAGAGCGTCGTGTAGGGAAAGAGTGT 3′
9bp-106B	5′ CTCTTTCCCTACACGACGCTCTTCCGATCTCAATGGAGAC 3′
10bp-429	5′ AATTGTAGATCCTTGAGATCGGAAGAGCGTCGTGTAGGGAAAGAGTGT 3′
10bp-429B	5′ CTCTTTCCCTACACGACGCTCTTCCGATCTCAAGGATCTAC 3′
8bp-107	5′ AATTGCTATATCGAGATCGGAAGAGCGTCGTGTAGGGAAAGAGTGT 3′
8bp-107B	5′ CTCTTTCCCTACACGACGCTCTTCCGATCTCGATATAGC 3′
9bp-107	5′ AATTGTCTATCTTAAGATCGGAAGAGCGTCGTGTAGGGAAAGAGTGT 3′
9bp-107B	5′ CTCTTTCCCTACACGACGCTCTTCCGATCTTAAGATAGAC 3′
10bp-431	5′ AATTGTAGATAGGCGAGATCGGAAGAGCGTCGTGTAGGGAAAGAGTGT 3′
10bp-431B	5′ CTCTTTCCCTACACGACGCTCTTCCGATCTCGCCTATCTAC 3′
8bp-108	5′ AATTGCTAAGAGTAGATCGGAAGAGCGTCGTGTAGGGAAAGAGTGT 3′
8bp-108B	5′ CTCTTTCCCTACACGACGCTCTTCCGATCTACTCTTAGC 3′

Supplementary Table 2 - *Continued from previous page*

**Oligo Name**	**Sequence**
9bp-108	5′ AATTGTCTAGAAGTAGATCGGAAGAGCGTCGTGTAGGGAAAGAGTGT 3′
9bp-108B	5′ CTCTTTCCCTACACGACGCTCTTCCGATCTACTTCTAGAC 3′
10bp-432	5′ AATTGTAGAGTAGGAAGATCGGAAGAGCGTCGTGTAGGGAAAGAGTGT 3′
10bp-432B	5′ CTCTTTCCCTACACGACGCTCTTCCGATCTTCCTACTCTAC 3′
8bp-109	5′ AATTGCGTCTCCGAGATCGGAAGAGCGTCGTGTAGGGAAAGAGTGT 3′
8bp-109B	5′ CTCTTTCCCTACACGACGCTCTTCCGATCTCGGAGACGC 3′
9bp-109	5′ AATTGTCTAAGTCTAGATCGGAAGAGCGTCGTGTAGGGAAAGAGTGT 3′
9bp-109B	5′ CTCTTTCCCTACACGACGCTCTTCCGATCTAGACTTAGAC 3′
10bp-433	5′ AATTGTAGAGACCAAAGATCGGAAGAGCGTCGTGTAGGGAAAGAGTGT 3′
10bp-433B	5′ CTCTTTCCCTACACGACGCTCTTCCGATCTTTGGTCTCTAC 3′
8bp-110	5′ AATTGCGTCAGACAGATCGGAAGAGCGTCGTGTAGGGAAAGAGTGT 3′
8bp-110B	5′ CTCTTTCCCTACACGACGCTCTTCCGATCTGTCTGACGC 3′
9bp-110	5′ AATTGTCGTATCCTAGATCGGAAGAGCGTCGTGTAGGGAAAGAGTGT 3′
9bp-110B	5′ CTCTTTCCCTACACGACGCTCTTCCGATCTAGGATACGAC 3′
10bp-435	5′ AATTGTAGACGGTCTAGATCGGAAGAGCGTCGTGTAGGGAAAGAGTGT 3′
10bp-435B	5′ CTCTTTCCCTACACGACGCTCTTCCGATCTAGACCGTCTAC 3′
8bp-111	5′ AATTGCGTATGGTAGATCGGAAGAGCGTCGTGTAGGGAAAGAGTGT 3′
8bp-111B	5′ CTCTTTCCCTACACGACGCTCTTCCGATCTACCATACGC 3′
9bp-111	5′ AATTGTCGTAGTTAAGATCGGAAGAGCGTCGTGTAGGGAAAGAGTGT 3′
9bp-111B	5′ CTCTTTCCCTACACGACGCTCTTCCGATCTTAACTACGAC 3′
10bp-440	5′ AATTGTACTATCGCTAGATCGGAAGAGCGTCGTGTAGGGAAAGAGTGT 3′
10bp-440B	5′ CTCTTTCCCTACACGACGCTCTTCCGATCTAGCGATAGTAC 3′
8bp-112	5′ AATTGCGTACTTCAGATCGGAAGAGCGTCGTGTAGGGAAAGAGTGT 3′
8bp-112B	5′ CTCTTTCCCTACACGACGCTCTTCCGATCTGAAGTACGC 3′
9bp-112	5′ AATTGTCGGTAATAAGATCGGAAGAGCGTCGTGTAGGGAAAGAGTGT 3′
9bp-112B	5′ CTCTTTCCCTACACGACGCTCTTCCGATCTTATTACCGAC 3′
10bp-441	5′ AATTGTACTAAGTAGAGATCGGAAGAGCGTCGTGTAGGGAAAGAGTGT 3′
10bp-441B	5′ CTCTTTCCCTACACGACGCTCTTCCGATCTCTACTTAGTAC 3′
8bp-113	5′ AATTGCGGTTACCAGATCGGAAGAGCGTCGTGTAGGGAAAGAGTGT 3′
8bp-113B	5′ CTCTTTCCCTACACGACGCTCTTCCGATCTGGTAACCGC 3′
9bp-113	5′ AATTGTCGGACGCGAGATCGGAAGAGCGTCGTGTAGGGAAAGAGTGT 3′
9bp-113B	5′ CTCTTTCCCTACACGACGCTCTTCCGATCTCGCGTCCGAC 3′
10bp-446	5′ AATTGTACCGGCTTAAGATCGGAAGAGCGTCGTGTAGGGAAAGAGTGT 3′
10bp-446B	5′ CTCTTTCCCTACACGACGCTCTTCCGATCTTAAGCCGGTAC 3′
8bp-114	5′ AATTGCGGACCAGAGATCGGAAGAGCGTCGTGTAGGGAAAGAGTGT 3′
8bp-114B	5′ CTCTTTCCCTACACGACGCTCTTCCGATCTCTGGTCCGC 3′
9bp-114	5′ AATTGTCGCTCGTCAGATCGGAAGAGCGTCGTGTAGGGAAAGAGTGT 3′
9bp-114B	5′ CTCTTTCCCTACACGACGCTCTTCCGATCTGACGAGCGAC 3′
10bp-447	5′ AATTGTACCAGCGACAGATCGGAAGAGCGTCGTGTAGGGAAAGAGTGT 3′
10bp-447B	5′ CTCTTTCCCTACACGACGCTCTTCCGATCTGTCGCTGGTAC 3′
8bp-115	5′ AATTGCGCTTCATAGATCGGAAGAGCGTCGTGTAGGGAAAGAGTGT 3′
8bp-115B	5′ CTCTTTCCCTACACGACGCTCTTCCGATCTATGAAGCGC 3′
9bp-115	5′ AATTGTCGCGAACGAGATCGGAAGAGCGTCGTGTAGGGAAAGAGTGT 3′
9bp-115B	5′ CTCTTTCCCTACACGACGCTCTTCCGATCTCGTTCGCGAC 3′
10bp-448	5′ AATTGTACCAGACCAAGATCGGAAGAGCGTCGTGTAGGGAAAGAGTGT 3′
10bp-448B	5′ CTCTTTCCCTACACGACGCTCTTCCGATCTTGGTCTGGTAC 3′
8bp-116	5′ AATTGCGCGAACTAGATCGGAAGAGCGTCGTGTAGGGAAAGAGTGT 3′
8bp-116B	5′ CTCTTTCCCTACACGACGCTCTTCCGATCTAGTTCGCGC 3′
9bp-116	5′ AATTGTCGCCTAGAAGATCGGAAGAGCGTCGTGTAGGGAAAGAGTGT 3′
9bp-116B	5′ CTCTTTCCCTACACGACGCTCTTCCGATCTTCTAGGCGAC 3′
10bp-449	5′ AATTGTACCAATGCGAGATCGGAAGAGCGTCGTGTAGGGAAAGAGTGT 3′
10bp-449B	5′ CTCTTTCCCTACACGACGCTCTTCCGATCTCGCATTGGTAC 3′
8bp-117	5′ AATTGCGCATATGAGATCGGAAGAGCGTCGTGTAGGGAAAGAGTGT 3′
8bp-117B	5′ CTCTTTCCCTACACGACGCTCTTCCGATCTCATATGCGC 3′
9bp-117	5′ AATTGTCGCCGCATAGATCGGAAGAGCGTCGTGTAGGGAAAGAGTGT 3′
9bp-117B	5′ CTCTTTCCCTACACGACGCTCTTCCGATCTATGCGGCGAC 3′
10bp-451	5′ AATTGTAATCGCGGAAGATCGGAAGAGCGTCGTGTAGGGAAAGAGTGT 3′
10bp-451B	5′ CTCTTTCCCTACACGACGCTCTTCCGATCTTCCGCGATTAC 3′
8bp-118	5′ AATTGCCTTCAACAGATCGGAAGAGCGTCGTGTAGGGAAAGAGTGT 3′
8bp-118B	5′ CTCTTTCCCTACACGACGCTCTTCCGATCTGTTGAAGGC 3′
9bp-118	5′ AATTGTCGATAGATAGATCGGAAGAGCGTCGTGTAGGGAAAGAGTGT 3′
9bp-118B	5′ CTCTTTCCCTACACGACGCTCTTCCGATCTATCTATCGAC 3′

Supplementary Table 2 - *Continued from previous page*

**Oligo Name**	**Sequence**
10bp-453	5′ AATTGTAAGTCGGCAAGATCGGAAGAGCGTCGTGTAGGGAAAGAGTGT 3′
10bp-453B	5′ CTCTTTCCCTACACGACGCTCTTCCGATCTTGCCGACTTAC 3′
8bp-119	5′ AATTGCCTGAGGAAGATCGGAAGAGCGTCGTGTAGGGAAAGAGTGT 3′
8bp-119B	5′ CTCTTTCCCTACACGACGCTCTTCCGATCTTCCTCAGGC 3′
9bp-119	5′ AATTGTCGACTTAGAGATCGGAAGAGCGTCGTGTAGGGAAAGAGTGT 3′
9bp-119B	5′ CTCTTTCCCTACACGACGCTCTTCCGATCTCTAAGTCGAC 3′
10bp-456	5′ AATTGTAAGCGCAACAGATCGGAAGAGCGTCGTGTAGGGAAAGAGTGT 3′
10bp-456B	5′ CTCTTTCCCTACACGACGCTCTTCCGATCTGTTGCGCTTAC 3′
8bp-120	5′ AATTGCCTAGACGAGATCGGAAGAGCGTCGTGTAGGGAAAGAGTGT 3′
8bp-120B	5′ CTCTTTCCCTACACGACGCTCTTCCGATCTCGTCTAGGC 3′
9bp-120	5′ AATTGTCCTTCAACAGATCGGAAGAGCGTCGTGTAGGGAAAGAGTGT 3′
9bp-120B	5′ CTCTTTCCCTACACGACGCTCTTCCGATCTGTTGAAGGAC 3′
10bp-457	5′ AATTGTAAGCCGAGGAGATCGGAAGAGCGTCGTGTAGGGAAAGAGTGT 3′
10bp-457B	5′ CTCTTTCCCTACACGACGCTCTTCCGATCTCCTCGGCTTAC 3′
8bp-121	5′ AATTGCCGGTTCTAGATCGGAAGAGCGTCGTGTAGGGAAAGAGTGT 3′
8bp-121B	5′ CTCTTTCCCTACACGACGCTCTTCCGATCTAGAACCGGC 3′
9bp-121	5′ AATTGTCCTCTCTGAGATCGGAAGAGCGTCGTGTAGGGAAAGAGTGT 3′
9bp-121B	5′ CTCTTTCCCTACACGACGCTCTTCCGATCTCAGAGAGGAC 3′
10bp-458	5′ AATTGTAAGCAATGAAGATCGGAAGAGCGTCGTGTAGGGAAAGAGTGT 3′
10bp-458B	5′ CTCTTTCCCTACACGACGCTCTTCCGATCTTCATTGCTTAC 3′
8bp-122	5′ AATTGCCGCCATTAGATCGGAAGAGCGTCGTGTAGGGAAAGAGTGT 3′
8bp-122B	5′ CTCTTTCCCTACACGACGCTCTTCCGATCTAATGGCGGC 3′
9bp-122	5′ AATTGTCCTAGACGAGATCGGAAGAGCGTCGTGTAGGGAAAGAGTGT 3′
9bp-122B	5′ CTCTTTCCCTACACGACGCTCTTCCGATCTCGTCTAGGAC 3′
10bp-459	5′ AATTGTAAGAGGTCCAGATCGGAAGAGCGTCGTGTAGGGAAAGAGTGT 3′
10bp-459B	5′ CTCTTTCCCTACACGACGCTCTTCCGATCTGGACCTCTTAC 3′
8bp-123	5′ AATTGCCGAATACAGATCGGAAGAGCGTCGTGTAGGGAAAGAGTGT 3′
8bp-123B	5′ CTCTTTCCCTACACGACGCTCTTCCGATCTGTATTCGGC 3′
9bp-123	5′ AATTGTCCGCCGTAAGATCGGAAGAGCGTCGTGTAGGGAAAGAGTGT 3′
9bp-123B	5′ CTCTTTCCCTACACGACGCTCTTCCGATCTTACGGCGGAC 3′
10bp-460	5′ AATTGTAAGAGACGTAGATCGGAAGAGCGTCGTGTAGGGAAAGAGTGT 3′
10bp-460B	5′ CTCTTTCCCTACACGACGCTCTTCCGATCTACGTCTCTTAC 3′
8bp-124	5′ AATTGCCAGGCTGAGATCGGAAGAGCGTCGTGTAGGGAAAGAGTGT 3′
8bp-124B	5′ CTCTTTCCCTACACGACGCTCTTCCGATCTCAGCCTGGC 3′
9bp-124	5′ AATTGTCCGCATCTAGATCGGAAGAGCGTCGTGTAGGGAAAGAGTGT 3′
9bp-124B	5′ CTCTTTCCCTACACGACGCTCTTCCGATCTAGATGCGGAC 3′
10bp-461	5′ AATTGTAACTTCTCCAGATCGGAAGAGCGTCGTGTAGGGAAAGAGTGT 3′
10bp-461B	5′ CTCTTTCCCTACACGACGCTCTTCCGATCTGGAGAAGTTAC 3′
8bp-125	5′ AATTGCCAGCGATAGATCGGAAGAGCGTCGTGTAGGGAAAGAGTGT 3′
8bp-125B	5′ CTCTTTCCCTACACGACGCTCTTCCGATCTATCGCTGGC 3′
9bp-125	5′ AATTGTCCGATAGCAGATCGGAAGAGCGTCGTGTAGGGAAAGAGTGT 3′
9bp-125B	5′ CTCTTTCCCTACACGACGCTCTTCCGATCTGCTATCGGAC 3′
10bp-462	5′ AATTGTAACGGTTAGAGATCGGAAGAGCGTCGTGTAGGGAAAGAGTGT 3′
10bp-462B	5′ CTCTTTCCCTACACGACGCTCTTCCGATCTCTAACCGTTAC 3′
8bp-126	5′ AATTGCCAACTCAAGATCGGAAGAGCGTCGTGTAGGGAAAGAGTGT 3′
8bp-126B	5′ CTCTTTCCCTACACGACGCTCTTCCGATCTTGAGTTGGC 3′
10bp-464	5′ AATTGTAACGAAGAAAGATCGGAAGAGCGTCGTGTAGGGAAAGAGTGT 3′
10bp-464B	5′ CTCTTTCCCTACACGACGCTCTTCCGATCTTTCTTCGTTAC 3′
8bp-127	5′ AATTGCATGGTTAAGATCGGAAGAGCGTCGTGTAGGGAAAGAGTGT 3′
8bp-127B	5′ CTCTTTCCCTACACGACGCTCTTCCGATCTTAACCATGC 3′
9bp-127	5′ AATTGTCATTGGCTAGATCGGAAGAGCGTCGTGTAGGGAAAGAGTGT 3′
9bp-127B	5′ CTCTTTCCCTACACGACGCTCTTCCGATCTAGCCAATGAC 3′
10bp-465	5′ AATTGTAACCGTAGTAGATCGGAAGAGCGTCGTGTAGGGAAAGAGTGT 3′
10bp-465B	5′ CTCTTTCCCTACACGACGCTCTTCCGATCTACTACGGTTAC 3′
8bp-128	5′ AATTGCAGTAGGTAGATCGGAAGAGCGTCGTGTAGGGAAAGAGTGT 3′
8bp-128B	5′ CTCTTTCCCTACACGACGCTCTTCCGATCTACCTACTGC 3′
9bp-128	5′ AATTGTCATTATGAAGATCGGAAGAGCGTCGTGTAGGGAAAGAGTGT 3′
9bp-128B	5′ CTCTTTCCCTACACGACGCTCTTCCGATCTTCATAATGAC 3′
10bp-466	5′ AATTGTAACCAGTTCAGATCGGAAGAGCGTCGTGTAGGGAAAGAGTGT 3′
10bp-466B	5′ CTCTTTCCCTACACGACGCTCTTCCGATCTGAACTGGTTAC 3′
9bp-129	5′ AATTGTCAGTTGAAAGATCGGAAGAGCGTCGTGTAGGGAAAGAGTGT 3′
9bp-129B	5′ CTCTTTCCCTACACGACGCTCTTCCGATCTTTCAACTGAC 3′

Supplementary Table 2 - *Continued from previous page*

**Oligo Name**	**Sequence**
10bp-467	5′ AATTGGTTGGTTGAAAGATCGGAAGAGCGTCGTGTAGGGAAAGAGTGT 3′
10bp-467B	5′ CTCTTTCCCTACACGACGCTCTTCCGATCTTTCAACCAACC 3′
8bp-130	5′ AATTGCAAGTAACAGATCGGAAGAGCGTCGTGTAGGGAAAGAGTGT 3′
8bp-130B	5′ CTCTTTCCCTACACGACGCTCTTCCGATCTGTTACTTGC 3′
9bp-130	5′ AATTGTCAAGTCATAGATCGGAAGAGCGTCGTGTAGGGAAAGAGTGT 3′
9bp-130B	5′ CTCTTTCCCTACACGACGCTCTTCCGATCTATGACTTGAC 3′
10bp-468	5′ AATTGGTTGGTCCTTAGATCGGAAGAGCGTCGTGTAGGGAAAGAGTGT 3′
10bp-468B	5′ CTCTTTCCCTACACGACGCTCTTCCGATCTAAGGACCAACC 3′
8bp-131	5′ AATTGATTACGCTAGATCGGAAGAGCGTCGTGTAGGGAAAGAGTGT 3′
8bp-131B	5′ CTCTTTCCCTACACGACGCTCTTCCGATCTAGCGTAATC 3′
9bp-131	5′ AATTGTCAACGCCGAGATCGGAAGAGCGTCGTGTAGGGAAAGAGTGT 3′
9bp-131B	5′ CTCTTTCCCTACACGACGCTCTTCCGATCTCGGCGTTGAC 3′
10bp-469	5′ AATTGGTTGGCCTCCAGATCGGAAGAGCGTCGTGTAGGGAAAGAGTGT 3′
10bp-469B	5′ CTCTTTCCCTACACGACGCTCTTCCGATCTGGAGGCCAACC 3′
8bp-132	5′ AATTGATTAACTGAGATCGGAAGAGCGTCGTGTAGGGAAAGAGTGT 3′
8bp-132B	5′ CTCTTTCCCTACACGACGCTCTTCCGATCTCAGTTAATC 3′
9bp-132	5′ AATTGTATTGGTTCAGATCGGAAGAGCGTCGTGTAGGGAAAGAGTGT 3′
9bp-132B	5′ CTCTTTCCCTACACGACGCTCTTCCGATCTGAACCAATAC 3′
10bp-472	5′ AATTGGTTGCGAGACAGATCGGAAGAGCGTCGTGTAGGGAAAGAGTGT 3′
10bp-472B	5′ CTCTTTCCCTACACGACGCTCTTCCGATCTGTCTCGCAACC 3′
8bp-133	5′ AATTGATGGAAGTAGATCGGAAGAGCGTCGTGTAGGGAAAGAGTGT 3′
8bp-133B	5′ CTCTTTCCCTACACGACGCTCTTCCGATCTACTTCCATC 3′
9bp-133	5′ AATTGTATTCTGCTAGATCGGAAGAGCGTCGTGTAGGGAAAGAGTGT 3′
9bp-133B	5′ CTCTTTCCCTACACGACGCTCTTCCGATCTAGCAGAATAC 3′
10bp-473	5′ AATTGGTTGCCGTAGAGATCGGAAGAGCGTCGTGTAGGGAAAGAGTGT 3′
10bp-473B	5′ CTCTTTCCCTACACGACGCTCTTCCGATCTCTACGGCAACC 3′
8bp-134	5′ AATTGATCCGCGCAGATCGGAAGAGCGTCGTGTAGGGAAAGAGTGT 3′
8bp-134B	5′ CTCTTTCCCTACACGACGCTCTTCCGATCTGCGCGGATC 3′
9bp-134	5′ AATTGTATTAGCCAAGATCGGAAGAGCGTCGTGTAGGGAAAGAGTGT 3′
9bp-134B	5′ CTCTTTCCCTACACGACGCTCTTCCGATCTTGGCTAATAC 3′
8bp-135	5′ AATTGATACGACGAGATCGGAAGAGCGTCGTGTAGGGAAAGAGTGT 3′
8bp-135B	5′ CTCTTTCCCTACACGACGCTCTTCCGATCTCGTCGTATC 3′
9bp-135	5′ AATTGTATTAATGGAGATCGGAAGAGCGTCGTGTAGGGAAAGAGTGT 3′
9bp-135B	5′ CTCTTTCCCTACACGACGCTCTTCCGATCTCCATTAATAC 3′
10bp-482	5′ AATTGGTTCTTGGCTAGATCGGAAGAGCGTCGTGTAGGGAAAGAGTGT 3′
10bp-482B	5′ CTCTTTCCCTACACGACGCTCTTCCGATCTAGCCAAGAACC 3′
8bp-136	5′ AATTGAGTTACGTAGATCGGAAGAGCGTCGTGTAGGGAAAGAGTGT 3′
8bp-136B	5′ CTCTTTCCCTACACGACGCTCTTCCGATCTACGTAACTC 3′
9bp-136	5′ AATTGTATGGTCAAAGATCGGAAGAGCGTCGTGTAGGGAAAGAGTGT 3′
9bp-136B	5′ CTCTTTCCCTACACGACGCTCTTCCGATCTTTGACCATAC 3′
10bp-484	5′ AATTGGTTCTGCCAAAGATCGGAAGAGCGTCGTGTAGGGAAAGAGTGT 3′
10bp-484B	5′ CTCTTTCCCTACACGACGCTCTTCCGATCTTTGGCAGAACC 3′
8bp-137	5′ AATTGAGTATAACAGATCGGAAGAGCGTCGTGTAGGGAAAGAGTGT 3′
8bp-137B	5′ CTCTTTCCCTACACGACGCTCTTCCGATCTGTTATACTC 3′
9bp-137	5′ AATTGTATGGCATTAGATCGGAAGAGCGTCGTGTAGGGAAAGAGTGT 3′
9bp-137B	5′ CTCTTTCCCTACACGACGCTCTTCCGATCTAATGCCATAC 3′
8bp-138	5′ AATTGAGCTAGAGAGATCGGAAGAGCGTCGTGTAGGGAAAGAGTGT 3′
8bp-138B	5′ CTCTTTCCCTACACGACGCTCTTCCGATCTCTCTAGCTC 3′
9bp-138	5′ AATTGTATGCCTCCAGATCGGAAGAGCGTCGTGTAGGGAAAGAGTGT 3′
9bp-138B	5′ CTCTTTCCCTACACGACGCTCTTCCGATCTGGAGGCATAC 3′
10bp-496	5′ AATTGGTTATGGCTTAGATCGGAAGAGCGTCGTGTAGGGAAAGAGTGT 3′
10bp-496B	5′ CTCTTTCCCTACACGACGCTCTTCCGATCTAAGCCATAACC 3′
8bp-139	5′ AATTGAGATGCCAAGATCGGAAGAGCGTCGTGTAGGGAAAGAGTGT 3′
8bp-139B	5′ CTCTTTCCCTACACGACGCTCTTCCGATCTTGGCATCTC 3′
9bp-139	5′ AATTGTATCTGGTTAGATCGGAAGAGCGTCGTGTAGGGAAAGAGTGT 3′
9bp-139B	5′ CTCTTTCCCTACACGACGCTCTTCCGATCTAACCAGATAC 3′
10bp-499	5′ AATTGGTTAGTTAGTAGATCGGAAGAGCGTCGTGTAGGGAAAGAGTGT 3′
10bp-499B	5′ CTCTTTCCCTACACGACGCTCTTCCGATCTACTAACTAACC 3′
8bp-140	5′ AATTGACTCATTCAGATCGGAAGAGCGTCGTGTAGGGAAAGAGTGT 3′
8bp-140B	5′ CTCTTTCCCTACACGACGCTCTTCCGATCTGAATGAGTC 3′
9bp-140	5′ AATTGTATCGGAAGAGATCGGAAGAGCGTCGTGTAGGGAAAGAGTGT 3′
9bp-140B	5′ CTCTTTCCCTACACGACGCTCTTCCGATCTCTTCCGATAC 3′

Supplementary Table 2 - *Continued from previous page*

Oligo Name	Sequence
10bp-501	5′ AATTGGTTAGCTCTAAGATCGGAAGAGCGTCGTGTAGGGAAAGAGTGT 3′
10bp-501B	5′ CTCTTTCCCTACACGACGCTCTTCCGATCTTAGAGCTAACC 3′
8bp-141	5′ AATTGACGACGGCAGATCGGAAGAGCGTCGTGTAGGGAAAGAGTGT 3′
8bp-141B	5′ CTCTTTCCCTACACGACGCTCTTCCGATCTGCCGTCGTC 3′
9bp-141	5′ AATTGTATCGCTGAAGATCGGAAGAGCGTCGTGTAGGGAAAGAGTGT 3′
9bp-141B	5′ CTCTTTCCCTACACGACGCTCTTCCGATCTTCAGCGATAC 3′
10bp-506	5′ AATTGGTCTTGATACAGATCGGAAGAGCGTCGTGTAGGGAAAGAGTGT 3′
10bp-506B	5′ CTCTTTCCCTACACGACGCTCTTCCGATCTGTATCAAGACC 3′
8bp-142	5′ AATTGACCGCCGGAGATCGGAAGAGCGTCGTGTAGGGAAAGAGTGT 3′
8bp-142B	5′ CTCTTTCCCTACACGACGCTCTTCCGATCTCCGGCGGTC 3′
9bp-142	5′ AATTGTATCGACCTAGATCGGAAGAGCGTCGTGTAGGGAAAGAGTGT 3′
9bp-142B	5′ CTCTTTCCCTACACGACGCTCTTCCGATCTAGGTCGATAC 3′
10bp-509	5′ AATTGGTCTGCTAATAGATCGGAAGAGCGTCGTGTAGGGAAAGAGTGT 3′
10bp-509B	5′ CTCTTTCCCTACACGACGCTCTTCCGATCTATTAGCAGACC 3′
8bp-143	5′ AATTGACCATAGTAGATCGGAAGAGCGTCGTGTAGGGAAAGAGTGT 3′
8bp-143B	5′ CTCTTTCCCTACACGACGCTCTTCCGATCTACTATGGTC 3′
9bp-143	5′ AATTGTATCCGCGCAGATCGGAAGAGCGTCGTGTAGGGAAAGAGTGT 3′
9bp-143B	5′ CTCTTTCCCTACACGACGCTCTTCCGATCTGCGCGGATAC 3′
10bp-512	5′ AATTGGTCTCCGACGAGATCGGAAGAGCGTCGTGTAGGGAAAGAGTGT 3′
10bp-512B	5′ CTCTTTCCCTACACGACGCTCTTCCGATCTCGTCGGAGACC 3′
8bp-144	5′ AATTGAATCTTCTAGATCGGAAGAGCGTCGTGTAGGGAAAGAGTGT 3′
8bp-144B	5′ CTCTTTCCCTACACGACGCTCTTCCGATCTAGAAGATTC 3′
9bp-144	5′ AATTGTATCAAGACAGATCGGAAGAGCGTCGTGTAGGGAAAGAGTGT 3′
9bp-144B	5′ CTCTTTCCCTACACGACGCTCTTCCGATCTGTCTTGATAC 3′
10bp-524	5′ AATTGGTCGATAGTAAGATCGGAAGAGCGTCGTGTAGGGAAAGAGTGT 3′
10bp-524B	5′ CTCTTTCCCTACACGACGCTCTTCCGATCTTACTATCGACC 3′
9bp-145	5′ AATTGTATATTATCAGATCGGAAGAGCGTCGTGTAGGGAAAGAGTGT 3′
9bp-145B	5′ CTCTTTCCCTACACGACGCTCTTCCGATCTGATAATATAC 3′
10bp-532	5′ AATTGGTCCAACGGAAGATCGGAAGAGCGTCGTGTAGGGAAAGAGTGT 3′
10bp-532B	5′ CTCTTTCCCTACACGACGCTCTTCCGATCTTCCGTTGGACC 3′
8bp-146	5′ AATTGAAGGATCCAGATCGGAAGAGCGTCGTGTAGGGAAAGAGTGT 3′
8bp-146B	5′ CTCTTTCCCTACACGACGCTCTTCCGATCTGGATCCTTC 3′
9bp-146	5′ AATTGTATATACGAAGATCGGAAGAGCGTCGTGTAGGGAAAGAGTGT 3′
9bp-146B	5′ CTCTTTCCCTACACGACGCTCTTCCGATCTTCGTATATAC 3′
10bp-538	5′ AATTGGTCAGAGGTTAGATCGGAAGAGCGTCGTGTAGGGAAAGAGTGT 3′
10bp-538B	5′ CTCTTTCCCTACACGACGCTCTTCCGATCTAACCTCTGACC 3′
8bp-147	5′ AATTGAAGCGTTGAGATCGGAAGAGCGTCGTGTAGGGAAAGAGTGT 3′
8bp-147B	5′ CTCTTTCCCTACACGACGCTCTTCCGATCTCAACGCTTC 3′
9bp-147	5′ AATTGTATACCGAGAGATCGGAAGAGCGTCGTGTAGGGAAAGAGTGT 3′
9bp-147B	5′ CTCTTTCCCTACACGACGCTCTTCCGATCTCTCGGTATAC 3′
10bp-565	5′ AATTGGTACCATTAAAGATCGGAAGAGCGTCGTGTAGGGAAAGAGTGT 3′
10bp-565B	5′ CTCTTTCCCTACACGACGCTCTTCCGATCTTTAATGGTACC 3′
8bp-148	5′ AATTGAACTGACCAGATCGGAAGAGCGTCGTGTAGGGAAAGAGTGT 3′
8bp-148B	5′ CTCTTTCCCTACACGACGCTCTTCCGATCTGGTCAGTTC 3′
9bp-148	5′ AATTGTAGTCATAAAGATCGGAAGAGCGTCGTGTAGGGAAAGAGTGT 3′
9bp-148B	5′ CTCTTTCCCTACACGACGCTCTTCCGATCTTTATGACTAC 3′
10bp-566	5′ AATTGGTAATCCGAAAGATCGGAAGAGCGTCGTGTAGGGAAAGAGTGT 3′
10bp-566B	5′ CTCTTTCCCTACACGACGCTCTTCCGATCTTTCGGATTACC 3′
8bp-149	5′ AATTGAACGCAATAGATCGGAAGAGCGTCGTGTAGGGAAAGAGTGT 3′
8bp-149B	5′ CTCTTTCCCTACACGACGCTCTTCCGATCTATTGCGTTC 3′
9bp-149	5′ AATTGTAGTAGAACAGATCGGAAGAGCGTCGTGTAGGGAAAGAGTGT 3′
9bp-149B	5′ CTCTTTCCCTACACGACGCTCTTCCGATCTGTTCTACTAC 3′
10bp-567	5′ AATTGGTAAGTCTTAAGATCGGAAGAGCGTCGTGTAGGGAAAGAGTGT 3′
10bp-567B	5′ CTCTTTCCCTACACGACGCTCTTCCGATCTTAAGACTTACC 3′
9bp-150	5′ AATTGTAGGCTATGAGATCGGAAGAGCGTCGTGTAGGGAAAGAGTGT 3′
9bp-150B	5′ CTCTTTCCCTACACGACGCTCTTCCGATCTCATAGCCTAC 3′
10bp-568	5′ AATTGGTAACTAACTAGATCGGAAGAGCGTCGTGTAGGGAAAGAGTGT 3′
10bp-568B	5′ CTCTTTCCCTACACGACGCTCTTCCGATCTAGTTAGTTACC 3′
9bp-151	5′ AATTGTAGGAATTCAGATCGGAAGAGCGTCGTGTAGGGAAAGAGTGT 3′
9bp-151B	5′ CTCTTTCCCTACACGACGCTCTTCCGATCTGAATTCCTAC 3′
10bp-570	5′ AATTGGGTTGGATATAGATCGGAAGAGCGTCGTGTAGGGAAAGAGTGT 3′
10bp-570B	5′ CTCTTTCCCTACACGACGCTCTTCCGATCTATATCCAACCC 3′

Supplementary Table 2 - *Continued from previous page*

Oligo Name	Sequence
9bp-152	5′ AATTGTAGATGACTAGATCGGAAGAGCGTCGTGTAGGGAAAGAGTGT 3′
9bp-152B	5′ CTCTTTCCCTACACGACGCTCTTCCGATCTAGTCATCTAC 3′
10bp-600	5′ AATTGGGTACGCTGAAGATCGGAAGAGCGTCGTGTAGGGAAAGAGTGT 3′
10bp-600B	5′ CTCTTTCCCTACACGACGCTCTTCCGATCTTCAGCGTACCC 3′
9bp-153	5′ AATTGTAGAGAGGCAGATCGGAAGAGCGTCGTGTAGGGAAAGAGTGT 3′
9bp-153B	5′ CTCTTTCCCTACACGACGCTCTTCCGATCTGCCTCTCTAC 3′
9bp-154	5′ AATTGTAGAATGCAAGATCGGAAGAGCGTCGTGTAGGGAAAGAGTGT 3′
9bp-154B	5′ CTCTTTCCCTACACGACGCTCTTCCGATCTTGCATTCTAC 3′
10bp-606	5′ AATTGGGCTTATCTGAGATCGGAAGAGCGTCGTGTAGGGAAAGAGTGT 3′
10bp-606B	5′ CTCTTTCCCTACACGACGCTCTTCCGATCTCAGATAAGCCC 3′
9bp-155	5′ AATTGTAGAACCTTAGATCGGAAGAGCGTCGTGTAGGGAAAGAGTGT 3′
9bp-155B	5′ CTCTTTCCCTACACGACGCTCTTCCGATCTAAGGTTCTAC 3′
10bp-610	5′ AATTGGGCTAGAAGGAGATCGGAAGAGCGTCGTGTAGGGAAAGAGTGT 3′
10bp-610B	5′ CTCTTTCCCTACACGACGCTCTTCCGATCTCCTTCTAGCCC 3′
9bp-156	5′ AATTGTACGGAAGGAGATCGGAAGAGCGTCGTGTAGGGAAAGAGTGT 3′
9bp-156B	5′ CTCTTTCCCTACACGACGCTCTTCCGATCTCCTTCCGTAC 3′
10bp-615	5′ AATTGGGCGGCGAGAAGATCGGAAGAGCGTCGTGTAGGGAAAGAGTGT 3′
10bp-615B	5′ CTCTTTCCCTACACGACGCTCTTCCGATCTTCTCGCCGCCC 3′
9bp-157	5′ AATTGTACCTCTATAGATCGGAAGAGCGTCGTGTAGGGAAAGAGTGT 3′
9bp-157B	5′ CTCTTTCCCTACACGACGCTCTTCCGATCTATAGAGGTAC 3′
10bp-622	5′ AATTGGGCGACCTGGAGATCGGAAGAGCGTCGTGTAGGGAAAGAGTGT 3′
10bp-622B	5′ CTCTTTCCCTACACGACGCTCTTCCGATCTCCAGGTCGCCC 3′
9bp-158	5′ AATTGTACCTACTGAGATCGGAAGAGCGTCGTGTAGGGAAAGAGTGT 3′
9bp-158B	5′ CTCTTTCCCTACACGACGCTCTTCCGATCTCAGTAGGTAC 3′
10bp-623	5′ AATTGGGCGAAGTTAAGATCGGAAGAGCGTCGTGTAGGGAAAGAGTGT 3′
10bp-623B	5′ CTCTTTCCCTACACGACGCTCTTCCGATCTTAACTTCGCCC 3′
9bp-159	5′ AATTGTAAGTAGCGAGATCGGAAGAGCGTCGTGTAGGGAAAGAGTGT 3′
9bp-159B	5′ CTCTTTCCCTACACGACGCTCTTCCGATCTCGCTACTTAC 3′
10bp-631	5′ AATTGGGCCAGAGAAAGATCGGAAGAGCGTCGTGTAGGGAAAGAGTGT 3′
10bp-631B	5′ CTCTTTCCCTACACGACGCTCTTCCGATCTTTCTCTGGCCC 3′
9bp-160	5′ AATTGTAAGCGAATAGATCGGAAGAGCGTCGTGTAGGGAAAGAGTGT 3′
9bp-160B	5′ CTCTTTCCCTACACGACGCTCTTCCGATCTATTCGCTTAC 3′
10bp-632	5′ AATTGGGCAGTCAGCAGATCGGAAGAGCGTCGTGTAGGGAAAGAGTGT 3′
10bp-632B	5′ CTCTTTCCCTACACGACGCTCTTCCGATCTGCTGACTGCCC 3′
9bp-161	5′ AATTGTAAGATGGTAGATCGGAAGAGCGTCGTGTAGGGAAAGAGTGT 3′
9bp-161B	5′ CTCTTTCCCTACACGACGCTCTTCCGATCTACCATCTTAC 3′
10bp-633	5′ AATTGGGCAGTAGAGAGATCGGAAGAGCGTCGTGTAGGGAAAGAGTGT 3′
10bp-633B	5′ CTCTTTCCCTACACGACGCTCTTCCGATCTCTCTACTGCCC 3′
9bp-162	5′ AATTGTAACCTCTTAGATCGGAAGAGCGTCGTGTAGGGAAAGAGTGT 3′
9bp-162B	5′ CTCTTTCCCTACACGACGCTCTTCCGATCTAAGAGGTTAC 3′
10bp-634	5′ AATTGGGCAGGAATTAGATCGGAAGAGCGTCGTGTAGGGAAAGAGTGT 3′
10bp-634B	5′ CTCTTTCCCTACACGACGCTCTTCCGATCTAATTCCTGCCC 3′
9bp-163	5′ AATTGTAACCAACCAGATCGGAAGAGCGTCGTGTAGGGAAAGAGTGT 3′
9bp-163B	5′ CTCTTTCCCTACACGACGCTCTTCCGATCTGGTTGGTTAC 3′
10bp-635	5′ AATTGGGCAGACTCTAGATCGGAAGAGCGTCGTGTAGGGAAAGAGTGT 3′
10bp-635B	5′ CTCTTTCCCTACACGACGCTCTTCCGATCTAGAGTCTGCCC 3′
9bp-164	5′ AATTGGTTGGTCGCAGATCGGAAGAGCGTCGTGTAGGGAAAGAGTGT 3′
9bp-164B	5′ CTCTTTCCCTACACGACGCTCTTCCGATCTGCGACCAACC 3′
10bp-637	5′ AATTGGGCAATCGCAAGATCGGAAGAGCGTCGTGTAGGGAAAGAGTGT 3′
10bp-637B	5′ CTCTTTCCCTACACGACGCTCTTCCGATCTTGCGATTGCCC 3′
9bp-165	5′ AATTGGTTGCGTAAAGATCGGAAGAGCGTCGTGTAGGGAAAGAGTGT 3′
9bp-165B	5′ CTCTTTCCCTACACGACGCTCTTCCGATCTTTACGCAACC 3′
10bp-639	5′ AATTGGGATGGCAGAAGATCGGAAGAGCGTCGTGTAGGGAAAGAGTGT 3′
10bp-639B	5′ CTCTTTCCCTACACGACGCTCTTCCGATCTTCTGCCATCCC 3′
9bp-166	5′ AATTGGTTGCAGTCAGATCGGAAGAGCGTCGTGTAGGGAAAGAGTGT 3′
9bp-166B	5′ CTCTTTCCCTACACGACGCTCTTCCGATCTGACTGCAACC 3′
10bp-641	5′ AATTGGGATGAGCAGAGATCGGAAGAGCGTCGTGTAGGGAAAGAGTGT 3′
10bp-641B	5′ CTCTTTCCCTACACGACGCTCTTCCGATCTCTGCTCATCCC 3′
10bp-643	5′ AATTGGGATCAATCTAGATCGGAAGAGCGTCGTGTAGGGAAAGAGTGT 3′
10bp-643B	5′ CTCTTTCCCTACACGACGCTCTTCCGATCTAGATTGATCCC 3′
9bp-168	5′ AATTGGTTCTTACGAGATCGGAAGAGCGTCGTGTAGGGAAAGAGTGT 3′
9bp-168B	5′ CTCTTTCCCTACACGACGCTCTTCCGATCTCGTAAGAACC 3′

Supplementary Table 2 - *Continued from previous page*

**Oligo Name**	**Sequence**
10bp-645	5′ AATTGGGATAATATTAGATCGGAAGAGCGTCGTGTAGGGAAAGAGTGT 3′
10bp-645B	5′ CTCTTTCCCTACACGACGCTCTTCCGATCTAATATTATCCC 3′
9bp-169	5′ AATTGGTTCTCGACAGATCGGAAGAGCGTCGTGTAGGGAAAGAGTGT 3′
9bp-169B	5′ CTCTTTCCCTACACGACGCTCTTCCGATCTGTCGAGAACC 3′
10bp-646	5′ AATTGGGAGTTCCATAGATCGGAAGAGCGTCGTGTAGGGAAAGAGTGT 3′
10bp-646B	5′ CTCTTTCCCTACACGACGCTCTTCCGATCTATGGAACTCCC 3′
9bp-170	5′ AATTGGTTCGGTTGAGATCGGAAGAGCGTCGTGTAGGGAAAGAGTGT 3′
9bp-170B	5′ CTCTTTCCCTACACGACGCTCTTCCGATCTCAACCGAACC 3′
10bp-647	5′ AATTGGGAGTTATTAAGATCGGAAGAGCGTCGTGTAGGGAAAGAGTGT 3′
10bp-647B	5′ CTCTTTCCCTACACGACGCTCTTCCGATCTTAATAACTCCC 3′
9bp-171	5′ AATTGGTTCCTCATAGATCGGAAGAGCGTCGTGTAGGGAAAGAGTGT 3′
9bp-171B	5′ CTCTTTCCCTACACGACGCTCTTCCGATCTATGAGGAACC 3′
10bp-650	5′ AATTGGGAGCCGCCAAGATCGGAAGAGCGTCGTGTAGGGAAAGAGTGT 3′
10bp-650B	5′ CTCTTTCCCTACACGACGCTCTTCCGATCTTGGCGGCTCCC 3′
9bp-172	5′ AATTGGTTCAGAGCAGATCGGAAGAGCGTCGTGTAGGGAAAGAGTGT 3′
9bp-172B	5′ CTCTTTCCCTACACGACGCTCTTCCGATCTGCTCTGAACC 3′
10bp-651	5′ AATTGGGAGACTGCGAGATCGGAAGAGCGTCGTGTAGGGAAAGAGTGT 3′
10bp-651B	5′ CTCTTTCCCTACACGACGCTCTTCCGATCTCGCAGTCTCCC 3′
9bp-173	5′ AATTGGTTCAATCAAGATCGGAAGAGCGTCGTGTAGGGAAAGAGTGT 3′
9bp-173B	5′ CTCTTTCCCTACACGACGCTCTTCCGATCTTGATTGAACC 3′
9bp-174	5′ AATTGGTTATCCGTAGATCGGAAGAGCGTCGTGTAGGGAAAGAGTGT 3′
9bp-174B	5′ CTCTTTCCCTACACGACGCTCTTCCGATCTACGGATAACC 3′
10bp-655	5′ AATTGGGACCGCCTAAGATCGGAAGAGCGTCGTGTAGGGAAAGAGTGT 3′
10bp-655B	5′ CTCTTTCCCTACACGACGCTCTTCCGATCTTAGGCGGTCCC 3′
9bp-175	5′ AATTGGTTAGGCCAAGATCGGAAGAGCGTCGTGTAGGGAAAGAGTGT 3′
9bp-175B	5′ CTCTTTCCCTACACGACGCTCTTCCGATCTTGGCCTAACC 3′
10bp-656	5′ AATTGGGACCAAGTCAGATCGGAAGAGCGTCGTGTAGGGAAAGAGTGT 3′
10bp-656B	5′ CTCTTTCCCTACACGACGCTCTTCCGATCTGACTTGGTCCC 3′
9bp-176	5′ AATTGGTTAGCAAGAGATCGGAAGAGCGTCGTGTAGGGAAAGAGTGT 3′
9bp-176B	5′ CTCTTTCCCTACACGACGCTCTTCCGATCTCTTGCTAACC 3′
10bp-658	5′ AATTGGGAATAATACAGATCGGAAGAGCGTCGTGTAGGGAAAGAGTGT 3′
10bp-658B	5′ CTCTTTCCCTACACGACGCTCTTCCGATCTGTATTATTCCC 3′
9bp-177	5′ AATTGGTTAATTGGAGATCGGAAGAGCGTCGTGTAGGGAAAGAGTGT 3′
9bp-177B	5′ CTCTTTCCCTACACGACGCTCTTCCGATCTCCAATTAACC 3′
10bp-659	5′ AATTGGGAAGAAGCAAGATCGGAAGAGCGTCGTGTAGGGAAAGAGTGT 3′
10bp-659B	5′ CTCTTTCCCTACACGACGCTCTTCCGATCTTGCTTCTTCCC 3′
9bp-178	5′ AATTGGTCTTATAGAGATCGGAAGAGCGTCGTGTAGGGAAAGAGTGT 3′
9bp-178B	5′ CTCTTTCCCTACACGACGCTCTTCCGATCTCTATAAGACC 3′
10bp-661	5′ AATTGGGAACGCGACAGATCGGAAGAGCGTCGTGTAGGGAAAGAGTGT 3′
10bp-661B	5′ CTCTTTCCCTACACGACGCTCTTCCGATCTGTCGCGTTCCC 3′
9bp-179	5′ AATTGGTCTGGAGAAGATCGGAAGAGCGTCGTGTAGGGAAAGAGTGT 3′
9bp-179B	5′ CTCTTTCCCTACACGACGCTCTTCCGATCTTCTCCAGACC 3′
10bp-662	5′ AATTGGCTTGCGAACAGATCGGAAGAGCGTCGTGTAGGGAAAGAGTGT 3′
10bp-662B	5′ CTCTTTCCCTACACGACGCTCTTCCGATCTGTTCGCAAGCC 3′
9bp-180	5′ AATTGGTCTCCGGTAGATCGGAAGAGCGTCGTGTAGGGAAAGAGTGT 3′
9bp-180B	5′ CTCTTTCCCTACACGACGCTCTTCCGATCTACCGGAGACC 3′
10bp-663	5′ AATTGGCTTGAATGGAGATCGGAAGAGCGTCGTGTAGGGAAAGAGTGT 3′
10bp-663B	5′ CTCTTTCCCTACACGACGCTCTTCCGATCTCCATTCAAGCC 3′
9bp-181	5′ AATTGGTCTATAACAGATCGGAAGAGCGTCGTGTAGGGAAAGAGTGT 3′
9bp-181B	5′ CTCTTTCCCTACACGACGCTCTTCCGATCTGTTATAGACC 3′
10bp-666	5′ AATTGGCTTATTGCGAGATCGGAAGAGCGTCGTGTAGGGAAAGAGTGT 3′
10bp-666B	5′ CTCTTTCCCTACACGACGCTCTTCCGATCTCGCAATAAGCC 3′
9bp-182	5′ AATTGGTCGCGACTAGATCGGAAGAGCGTCGTGTAGGGAAAGAGTGT 3′
9bp-182B	5′ CTCTTTCCCTACACGACGCTCTTCCGATCTAGTCGCGACC 3′
10bp-667	5′ AATTGGCTTAGCCTCAGATCGGAAGAGCGTCGTGTAGGGAAAGAGTGT 3′
10bp-667B	5′ CTCTTTCCCTACACGACGCTCTTCCGATCTGAGGCTAAGCC 3′
9bp-183	5′ AATTGGTCGAGGAGAGATCGGAAGAGCGTCGTGTAGGGAAAGAGTGT 3′
9bp-183B	5′ CTCTTTCCCTACACGACGCTCTTCCGATCTCTCCTCGACC 3′
10bp-668	5′ AATTGGCTTACGTCTAGATCGGAAGAGCGTCGTGTAGGGAAAGAGTGT 3′
10bp-668B	5′ CTCTTTCCCTACACGACGCTCTTCCGATCTAGACGTAAGCC 3′
9bp-184	5′ AATTGGTCGAACGTAGATCGGAAGAGCGTCGTGTAGGGAAAGAGTGT 3′
9bp-184B	5′ CTCTTTCCCTACACGACGCTCTTCCGATCTACGTTCGACC 3′

Supplementary Table 2 - *Continued from previous page*

**Oligo Name**	**Sequence**
10bp-669	5′ AATTGGCTGCGGACCAGATCGGAAGAGCGTCGTGTAGGGAAAGAGTGT 3′
10bp-669B	5′ CTCTTTCCCTACACGACGCTCTTCCGATCTGGTCCGCAGCC 3′
9bp-185	5′ AATTGGTCCTAATCAGATCGGAAGAGCGTCGTGTAGGGAAAGAGTGT 3′
9bp-185B	5′ CTCTTTCCCTACACGACGCTCTTCCGATCTGATTAGGACC 3′
10bp-670	5′ AATTGGCTGCCTAGTAGATCGGAAGAGCGTCGTGTAGGGAAAGAGTGT 3′
10bp-670B	5′ CTCTTTCCCTACACGACGCTCTTCCGATCTACTAGGCAGCC 3′
9bp-186	5′ AATTGGTCCGCTAAAGATCGGAAGAGCGTCGTGTAGGGAAAGAGTGT 3′
9bp-186B	5′ CTCTTTCCCTACACGACGCTCTTCCGATCTTTAGCGGACC 3′
10bp-675	5′ AATTGGCTCTAGCCAAGATCGGAAGAGCGTCGTGTAGGGAAAGAGTGT 3′
10bp-675B	5′ CTCTTTCCCTACACGACGCTCTTCCGATCTTGGCTAGAGCC 3′
9bp-187	5′ AATTGGTCCGAGCTAGATCGGAAGAGCGTCGTGTAGGGAAAGAGTGT 3′
9bp-187B	5′ CTCTTTCCCTACACGACGCTCTTCCGATCTAGCTCGGACC 3′
10bp-676	5′ AATTGGCTCGTCATCAGATCGGAAGAGCGTCGTGTAGGGAAAGAGTGT 3′
10bp-676B	5′ CTCTTTCCCTACACGACGCTCTTCCGATCTGATGACGAGCC 3′
9bp-188	5′ AATTGGTCCAGCTAAGATCGGAAGAGCGTCGTGTAGGGAAAGAGTGT 3′
9bp-188B	5′ CTCTTTCCCTACACGACGCTCTTCCGATCTTAGCTGGACC 3′
10bp-679	5′ AATTGGCTCCGTCCTAGATCGGAAGAGCGTCGTGTAGGGAAAGAGTGT 3′
10bp-679B	5′ CTCTTTCCCTACACGACGCTCTTCCGATCTAGGACGGAGCC 3′
9bp-189	5′ AATTGGTCAGCCTCAGATCGGAAGAGCGTCGTGTAGGGAAAGAGTGT 3′
9bp-189B	5′ CTCTTTCCCTACACGACGCTCTTCCGATCTGAGGCTGACC 3′
10bp-685	5′ AATTGGCTACCATGCAGATCGGAAGAGCGTCGTGTAGGGAAAGAGTGT 3′
10bp-685B	5′ CTCTTTCCCTACACGACGCTCTTCCGATCTGCATGGTAGCC 3′
9bp-190	5′ AATTGGTCAACTCTAGATCGGAAGAGCGTCGTGTAGGGAAAGAGTGT 3′
9bp-190B	5′ CTCTTTCCCTACACGACGCTCTTCCGATCTAGAGTTGACC 3′
10bp-692	5′ AATTGGCGTCTAGACAGATCGGAAGAGCGTCGTGTAGGGAAAGAGTGT 3′
10bp-692B	5′ CTCTTTCCCTACACGACGCTCTTCCGATCTGTCTAGACGCC 3′
9bp-191	5′ AATTGGTATCTGCAAGATCGGAAGAGCGTCGTGTAGGGAAAGAGTGT 3′
9bp-191B	5′ CTCTTTCCCTACACGACGCTCTTCCGATCTTGCAGATACC 3′
10bp-694	5′ AATTGGCGTCAGATAAGATCGGAAGAGCGTCGTGTAGGGAAAGAGTGT 3′
10bp-694B	5′ CTCTTTCCCTACACGACGCTCTTCCGATCTTATCTGACGCC 3′
9bp-192	5′ AATTGGTATACTTCAGATCGGAAGAGCGTCGTGTAGGGAAAGAGTGT 3′
9bp-192B	5′ CTCTTTCCCTACACGACGCTCTTCCGATCTGAAGTATACC 3′
10bp-696	5′ AATTGGCGTACCGCAAGATCGGAAGAGCGTCGTGTAGGGAAAGAGTGT 3′
10bp-696B	5′ CTCTTTCCCTACACGACGCTCTTCCGATCTTGCGGTACGCC 3′
9bp-193	5′ AATTGGTAGGCTCGAGATCGGAAGAGCGTCGTGTAGGGAAAGAGTGT 3′
9bp-193B	5′ CTCTTTCCCTACACGACGCTCTTCCGATCTCGAGCCTACC 3′
10bp-702	5′ AATTGGCGGCTTCAAAGATCGGAAGAGCGTCGTGTAGGGAAAGAGTGT 3′
10bp-702B	5′ CTCTTTCCCTACACGACGCTCTTCCGATCTTTGAAGCCGCC 3′
9bp-194	5′ AATTGGTAGCTAGGAGATCGGAAGAGCGTCGTGTAGGGAAAGAGTGT 3′
9bp-194B	5′ CTCTTTCCCTACACGACGCTCTTCCGATCTCCTAGCTACC 3′
10bp-710	5′ AATTGGCGCTGCCGGAGATCGGAAGAGCGTCGTGTAGGGAAAGAGTGT 3′
10bp-710B	5′ CTCTTTCCCTACACGACGCTCTTCCGATCTCCGGCAGCGCC 3′
9bp-195	5′ AATTGGTACTGCCTAGATCGGAAGAGCGTCGTGTAGGGAAAGAGTGT 3′
9bp-195B	5′ CTCTTTCCCTACACGACGCTCTTCCGATCTAGGCAGTACC 3′
10bp-712	5′ AATTGGCGCGTTGCTAGATCGGAAGAGCGTCGTGTAGGGAAAGAGTGT 3′
10bp-712B	5′ CTCTTTCCCTACACGACGCTCTTCCGATCTAGCAACGCGCC 3′
9bp-196	5′ AATTGGTAATTCAAAGATCGGAAGAGCGTCGTGTAGGGAAAGAGTGT 3′
9bp-196B	5′ CTCTTTCCCTACACGACGCTCTTCCGATCTTTGAATTACC 3′
10bp-713	5′ AATTGGCGCGGTTGCAGATCGGAAGAGCGTCGTGTAGGGAAAGAGTGT 3′
10bp-713B	5′ CTCTTTCCCTACACGACGCTCTTCCGATCTGCAACCGCGCC 3′
9bp-197	5′ AATTGGTAAGATTAAGATCGGAAGAGCGTCGTGTAGGGAAAGAGTGT 3′
9bp-197B	5′ CTCTTTCCCTACACGACGCTCTTCCGATCTTAATCTTACC 3′
10bp-717	5′ AATTGGCGCATATTCAGATCGGAAGAGCGTCGTGTAGGGAAAGAGTGT 3′
10bp-717B	5′ CTCTTTCCCTACACGACGCTCTTCCGATCTGAATATGCGCC 3′
9bp-198	5′ AATTGGTAACGGATAGATCGGAAGAGCGTCGTGTAGGGAAAGAGTGT 3′
9bp-198B	5′ CTCTTTCCCTACACGACGCTCTTCCGATCTATCCGTTACC 3′
10bp-724	5′ AATTGGCGACGCGTAAGATCGGAAGAGCGTCGTGTAGGGAAAGAGTGT 3′
10bp-724B	5′ CTCTTTCCCTACACGACGCTCTTCCGATCTTACGCGTCGCC 3′
9bp-199	5′ AATTGGGTTGGCTTAGATCGGAAGAGCGTCGTGTAGGGAAAGAGTGT 3′
9bp-199B	5′ CTCTTTCCCTACACGACGCTCTTCCGATCTAAGCCAACCC 3′
10bp-725	5′ AATTGGCGAAGACGTAGATCGGAAGAGCGTCGTGTAGGGAAAGAGTGT 3′
10bp-725B	5′ CTCTTTCCCTACACGACGCTCTTCCGATCTACGTCTTCGCC 3′

Supplementary Table 2 - *Continued from previous page*

**Oligo Name**	**Sequence**
9bp-200	5′ AATTGGGTTGAAGGAGATCGGAAGAGCGTCGTGTAGGGAAAGAGTGT 3′
9bp-200B	5′ CTCTTTCCCTACACGACGCTCTTCCGATCTCCTTCAACCC 3′
10bp-726	5′ AATTGGCGAACCATGAGATCGGAAGAGCGTCGTGTAGGGAAAGAGTGT 3′
10bp-726B	5′ CTCTTTCCCTACACGACGCTCTTCCGATCTCATGGTTCGCC 3′
9bp-201	5′ AATTGGGTTCTTCGAGATCGGAAGAGCGTCGTGTAGGGAAAGAGTGT 3′
9bp-201B	5′ CTCTTTCCCTACACGACGCTCTTCCGATCTCGAAGAACCC 3′
10bp-727	5′ AATTGGCCTTCGTAAAGATCGGAAGAGCGTCGTGTAGGGAAAGAGTGT 3′
10bp-727B	5′ CTCTTTCCCTACACGACGCTCTTCCGATCTTTACGAAGGCC 3′
9bp-202	5′ AATTGGGTTCCAATAGATCGGAAGAGCGTCGTGTAGGGAAAGAGTGT 3′
9bp-202B	5′ CTCTTTCCCTACACGACGCTCTTCCGATCTATTGGAACCC 3′
10bp-730	5′ AATTGGCCTGACTTCAGATCGGAAGAGCGTCGTGTAGGGAAAGAGTGT 3′
10bp-730B	5′ CTCTTTCCCTACACGACGCTCTTCCGATCTGAAGTCAGGCC 3′
9bp-203	5′ AATTGGGTTATATAAGATCGGAAGAGCGTCGTGTAGGGAAAGAGTGT 3′
9bp-203B	5′ CTCTTTCCCTACACGACGCTCTTCCGATCTTATATAACCC 3′
10bp-734	5′ AATTGGCCGGCGGATAGATCGGAAGAGCGTCGTGTAGGGAAAGAGTGT 3′
10bp-734B	5′ CTCTTTCCCTACACGACGCTCTTCCGATCTATCCGCCGGCC 3′
9bp-204	5′ AATTGGGTTAATACAGATCGGAAGAGCGTCGTGTAGGGAAAGAGTGT 3′
9bp-204B	5′ CTCTTTCCCTACACGACGCTCTTCCGATCTGTATTAACCC 3′
10bp-735	5′ AATTGGCCGGATATGAGATCGGAAGAGCGTCGTGTAGGGAAAGAGTGT 3′
10bp-735B	5′ CTCTTTCCCTACACGACGCTCTTCCGATCTCATATCCGGCC 3′
9bp-205	5′ AATTGGGTCGCGGTAGATCGGAAGAGCGTCGTGTAGGGAAAGAGTGT 3′
9bp-205B	5′ CTCTTTCCCTACACGACGCTCTTCCGATCTACCGCGACCC 3′
10bp-736	5′ AATTGGCCGCTCATAAGATCGGAAGAGCGTCGTGTAGGGAAAGAGTGT 3′
10bp-736B	5′ CTCTTTCCCTACACGACGCTCTTCCGATCTTATGAGCGGCC 3′
9bp-206	5′ AATTGGGTATTGATAGATCGGAAGAGCGTCGTGTAGGGAAAGAGTGT 3′
9bp-206B	5′ CTCTTTCCCTACACGACGCTCTTCCGATCTATCAATACCC 3′
10bp-738	5′ AATTGGCCGAGAGGCAGATCGGAAGAGCGTCGTGTAGGGAAAGAGTGT 3′
10bp-738B	5′ CTCTTTCCCTACACGACGCTCTTCCGATCTGCCTCTCGGCC 3′
9bp-207	5′ AATTGGGTAGATCTAGATCGGAAGAGCGTCGTGTAGGGAAAGAGTGT 3′
9bp-207B	5′ CTCTTTCCCTACACGACGCTCTTCCGATCTAGATCTACCC 3′
10bp-739	5′ AATTGGCCATCTTCGAGATCGGAAGAGCGTCGTGTAGGGAAAGAGTGT 3′
10bp-739B	5′ CTCTTTCCCTACACGACGCTCTTCCGATCTCGAAGATGGCC 3′
9bp-208	5′ AATTGGGTACTCTCAGATCGGAAGAGCGTCGTGTAGGGAAAGAGTGT 3′
9bp-208B	5′ CTCTTTCCCTACACGACGCTCTTCCGATCTGAGAGTACCC 3′
10bp-740	5′ AATTGGCCAAGCTCCAGATCGGAAGAGCGTCGTGTAGGGAAAGAGTGT 3′
10bp-740B	5′ CTCTTTCCCTACACGACGCTCTTCCGATCTGGAGCTTGGCC 3′
9bp-209	5′ AATTGGGTAACCAAAGATCGGAAGAGCGTCGTGTAGGGAAAGAGTGT 3′
9bp-209B	5′ CTCTTTCCCTACACGACGCTCTTCCGATCTTTGGTTACCC 3′
10bp-743	5′ AATTGGCATCCGCGCAGATCGGAAGAGCGTCGTGTAGGGAAAGAGTGT 3′
10bp-743B	5′ CTCTTTCCCTACACGACGCTCTTCCGATCTGCGCGGATGCC 3′
10bp-744	5′ AATTGGCATAATCAAAGATCGGAAGAGCGTCGTGTAGGGAAAGAGTGT 3′
10bp-744B	5′ CTCTTTCCCTACACGACGCTCTTCCGATCTTTGATTATGCC 3′
9bp-211	5′ AATTGGGCTTACGCAGATCGGAAGAGCGTCGTGTAGGGAAAGAGTGT 3′
9bp-211B	5′ CTCTTTCCCTACACGACGCTCTTCCGATCTGCGTAAGCCC 3′
10bp-745	5′ AATTGGCAGTAACGCAGATCGGAAGAGCGTCGTGTAGGGAAAGAGTGT 3′
10bp-745B	5′ CTCTTTCCCTACACGACGCTCTTCCGATCTGCGTTACTGCC 3′
9bp-212	5′ AATTGGGCTCGATGAGATCGGAAGAGCGTCGTGTAGGGAAAGAGTGT 3′
9bp-212B	5′ CTCTTTCCCTACACGACGCTCTTCCGATCTCATCGAGCCC 3′
10bp-747	5′ AATTGGCAGGCTACCAGATCGGAAGAGCGTCGTGTAGGGAAAGAGTGT 3′
10bp-747B	5′ CTCTTTCCCTACACGACGCTCTTCCGATCTGGTAGCCTGCC 3′
9bp-213	5′ AATTGGGCGTCCTTAGATCGGAAGAGCGTCGTGTAGGGAAAGAGTGT 3′
9bp-213B	5′ CTCTTTCCCTACACGACGCTCTTCCGATCTAAGGACGCCC 3′
10bp-748	5′ AATTGGCAGCGCTAAAGATCGGAAGAGCGTCGTGTAGGGAAAGAGTGT 3′
10bp-748B	5′ CTCTTTCCCTACACGACGCTCTTCCGATCTTTAGCGCTGCC 3′
9bp-214	5′ AATTGGGCGGTTCCAGATCGGAAGAGCGTCGTGTAGGGAAAGAGTGT 3′
9bp-214B	5′ CTCTTTCCCTACACGACGCTCTTCCGATCTGGAACCGCCC 3′
10bp-749	5′ AATTGGCAGAGGATGAGATCGGAAGAGCGTCGTGTAGGGAAAGAGTGT 3′
10bp-749B	5′ CTCTTTCCCTACACGACGCTCTTCCGATCTCATCCTCTGCC 3′
9bp-215	5′ AATTGGGCCTTCAGAGATCGGAAGAGCGTCGTGTAGGGAAAGAGTGT 3′
9bp-215B	5′ CTCTTTCCCTACACGACGCTCTTCCGATCTCTGAAGGCCC 3′
10bp-751	5′ AATTGGCAATTGGTTAGATCGGAAGAGCGTCGTGTAGGGAAAGAGTGT 3′
10bp-751B	5′ CTCTTTCCCTACACGACGCTCTTCCGATCTAACCAATTGCC 3′

Supplementary Table 2 - *Continued from previous page*

**Oligo Name**	**Sequence**
9bp-216	5′ AATTGGGCCATGGCAGATCGGAAGAGCGTCGTGTAGGGAAAGAGTGT 3′
9bp-216B	5′ CTCTTTCCCTACACGACGCTCTTCCGATCTGCCATGGCCC 3′
10bp-752	5′ AATTGGCAATGAATCAGATCGGAAGAGCGTCGTGTAGGGAAAGAGTGT 3′
10bp-752B	5′ CTCTTTCCCTACACGACGCTCTTCCGATCTGATTCATTGCC 3′
9bp-217	5′ AATTGGGCCAGTCGAGATCGGAAGAGCGTCGTGTAGGGAAAGAGTGT 3′
9bp-217B	5′ CTCTTTCCCTACACGACGCTCTTCCGATCTCGACTGGCCC 3′
10bp-753	5′ AATTGGCAAGGAGATAGATCGGAAGAGCGTCGTGTAGGGAAAGAGTGT 3′
10bp-753B	5′ CTCTTTCCCTACACGACGCTCTTCCGATCTATCTCCTTGCC 3′
9bp-218	5′ AATTGGGCATGAGTAGATCGGAAGAGCGTCGTGTAGGGAAAGAGTGT 3′
9bp-218B	5′ CTCTTTCCCTACACGACGCTCTTCCGATCTACTCATGCCC 3′
10bp-754	5′ AATTGGCAAGCTTGAAGATCGGAAGAGCGTCGTGTAGGGAAAGAGTGT 3′
10bp-754B	5′ CTCTTTCCCTACACGACGCTCTTCCGATCTTCAAGCTTGCC 3′
9bp-219	5′ AATTGGGATGGACCAGATCGGAAGAGCGTCGTGTAGGGAAAGAGTGT 3′
9bp-219B	5′ CTCTTTCCCTACACGACGCTCTTCCGATCTGGTCCATCCC 3′
10bp-756	5′ AATTGGCAACCTCATAGATCGGAAGAGCGTCGTGTAGGGAAAGAGTGT 3′
10bp-756B	5′ CTCTTTCCCTACACGACGCTCTTCCGATCTATGAGGTTGCC 3′
9bp-220	5′ AATTGGGATATCGTAGATCGGAAGAGCGTCGTGTAGGGAAAGAGTGT 3′
9bp-220B	5′ CTCTTTCCCTACACGACGCTCTTCCGATCTACGATATCCC 3′
10bp-758	5′ AATTGGATTGGACCAAGATCGGAAGAGCGTCGTGTAGGGAAAGAGTGT 3′
10bp-758B	5′ CTCTTTCCCTACACGACGCTCTTCCGATCTTGGTCCAATCC 3′
9bp-221	5′ AATTGGGAGTTAACAGATCGGAAGAGCGTCGTGTAGGGAAAGAGTGT 3′
9bp-221B	5′ CTCTTTCCCTACACGACGCTCTTCCGATCTGTTAACTCCC 3′
10bp-765	5′ AATTGGATGATTCTCAGATCGGAAGAGCGTCGTGTAGGGAAAGAGTGT 3′
10bp-765B	5′ CTCTTTCCCTACACGACGCTCTTCCGATCTGAGAATCATCC 3′
9bp-222	5′ AATTGGGAGGAGATAGATCGGAAGAGCGTCGTGTAGGGAAAGAGTGT 3′
9bp-222B	5′ CTCTTTCCCTACACGACGCTCTTCCGATCTATCTCCTCCC 3′
10bp-768	5′ AATTGGATCTAGGAGAGATCGGAAGAGCGTCGTGTAGGGAAAGAGTGT 3′
10bp-768B	5′ CTCTTTCCCTACACGACGCTCTTCCGATCTCTCCTAGATCC 3′
9bp-223	5′ AATTGGGAGCGTTCAGATCGGAAGAGCGTCGTGTAGGGAAAGAGTGT 3′
9bp-223B	5′ CTCTTTCCCTACACGACGCTCTTCCGATCTGAACGCTCCC 3′
10bp-769	5′ AATTGGATCGTAAGAAGATCGGAAGAGCGTCGTGTAGGGAAAGAGTGT 3′
10bp-769B	5′ CTCTTTCCCTACACGACGCTCTTCCGATCTTCTTACGATCC 3′
10bp-770	5′ AATTGGATCCATATTAGATCGGAAGAGCGTCGTGTAGGGAAAGAGTGT 3′
10bp-770B	5′ CTCTTTCCCTACACGACGCTCTTCCGATCTAATATGGATCC 3′
9bp-225	5′ AATTGGGAGAACCGAGATCGGAAGAGCGTCGTGTAGGGAAAGAGTGT 3′
9bp-225B	5′ CTCTTTCCCTACACGACGCTCTTCCGATCTCGGTTCTCCC 3′
10bp-773	5′ AATTGGATATCAATAAGATCGGAAGAGCGTCGTGTAGGGAAAGAGTGT 3′
10bp-773B	5′ CTCTTTCCCTACACGACGCTCTTCCGATCTTATTGATATCC 3′
9bp-226	5′ AATTGGGACTCTTGAGATCGGAAGAGCGTCGTGTAGGGAAAGAGTGT 3′
9bp-226B	5′ CTCTTTCCCTACACGACGCTCTTCCGATCTCAAGAGTCCC 3′
10bp-774	5′ AATTGGATATATGCCAGATCGGAAGAGCGTCGTGTAGGGAAAGAGTGT 3′
10bp-774B	5′ CTCTTTCCCTACACGACGCTCTTCCGATCTGGCATATATCC 3′
9bp-227	5′ AATTGGGACGGCGGAGATCGGAAGAGCGTCGTGTAGGGAAAGAGTGT 3′
9bp-227B	5′ CTCTTTCCCTACACGACGCTCTTCCGATCTCCGCCGTCCC 3′
10bp-776	5′ AATTGGATACGAAGTAGATCGGAAGAGCGTCGTGTAGGGAAAGAGTGT 3′
10bp-776B	5′ CTCTTTCCCTACACGACGCTCTTCCGATCTACTTCGTATCC 3′
9bp-228	5′ AATTGGGACCTTGAAGATCGGAAGAGCGTCGTGTAGGGAAAGAGTGT 3′
9bp-228B	5′ CTCTTTCCCTACACGACGCTCTTCCGATCTTCAAGGTCCC 3′
10bp-783	5′ AATTGGAGTATGGTCAGATCGGAAGAGCGTCGTGTAGGGAAAGAGTGT 3′
10bp-783B	5′ CTCTTTCCCTACACGACGCTCTTCCGATCTGACCATACTCC 3′
9bp-229	5′ AATTGGGAATGGCGAGATCGGAAGAGCGTCGTGTAGGGAAAGAGTGT 3′
9bp-229B	5′ CTCTTTCCCTACACGACGCTCTTCCGATCTCGCCATTCCC 3′
10bp-784	5′ AATTGGAGTATATAAAGATCGGAAGAGCGTCGTGTAGGGAAAGAGTGT 3′
10bp-784B	5′ CTCTTTCCCTACACGACGCTCTTCCGATCTTTATATACTCC 3′
9bp-230	5′ AATTGGGAAGTTAGAGATCGGAAGAGCGTCGTGTAGGGAAAGAGTGT 3′
9bp-230B	5′ CTCTTTCCCTACACGACGCTCTTCCGATCTCTAACTTCCC 3′
9bp-231	5′ AATTGGGAACTACTAGATCGGAAGAGCGTCGTGTAGGGAAAGAGTGT 3′
9bp-231B	5′ CTCTTTCCCTACACGACGCTCTTCCGATCTAGTAGTTCCC 3′
10bp-788	5′ AATTGGAGGTCCGGAAGATCGGAAGAGCGTCGTGTAGGGAAAGAGTGT 3′
10bp-788B	5′ CTCTTTCCCTACACGACGCTCTTCCGATCTTCCGGACCTCC 3′
9bp-232	5′ AATTGGCTTCAGGAAGATCGGAAGAGCGTCGTGTAGGGAAAGAGTGT 3′
9bp-232B	5′ CTCTTTCCCTACACGACGCTCTTCCGATCTTCCTGAAGCC 3′

Supplementary Table 2 - *Continued from previous page*

**Oligo Name**	**Sequence**
10bp-791	5′ AATTGGAGGCAGCCTAGATCGGAAGAGCGTCGTGTAGGGAAAGAGTGT 3′
10bp-791B	5′ CTCTTTCCCTACACGACGCTCTTCCGATCTAGGCTGCCTCC 3′
9bp-233	5′ AATTGGCTGCTACCAGATCGGAAGAGCGTCGTGTAGGGAAAGAGTGT 3′
9bp-233B	5′ CTCTTTCCCTACACGACGCTCTTCCGATCTGGTAGCAGCC 3′
10bp-793	5′ AATTGGAGGAATTGGAGATCGGAAGAGCGTCGTGTAGGGAAAGAGTGT 3′
10bp-793B	5′ CTCTTTCCCTACACGACGCTCTTCCGATCTCCAATTCCTCC 3′
9bp-234	5′ AATTGGCTGCGCTGAGATCGGAAGAGCGTCGTGTAGGGAAAGAGTGT 3′
9bp-234B	5′ CTCTTTCCCTACACGACGCTCTTCCGATCTCAGCGCAGCC 3′
10bp-800	5′ AATTGGAGCATTATGAGATCGGAAGAGCGTCGTGTAGGGAAAGAGTGT 3′
10bp-800B	5′ CTCTTTCCCTACACGACGCTCTTCCGATCTCATAATGCTCC 3′
9bp-235	5′ AATTGGCTGAGAATAGATCGGAAGAGCGTCGTGTAGGGAAAGAGTGT 3′
9bp-235B	5′ CTCTTTCCCTACACGACGCTCTTCCGATCTATTCTCAGCC 3′
10bp-801	5′ AATTGGAGCAAGTACAGATCGGAAGAGCGTCGTGTAGGGAAAGAGTGT 3′
10bp-801B	5′ CTCTTTCCCTACACGACGCTCTTCCGATCTGTACTTGCTCC 3′
9bp-236	5′ AATTGGCTGACTGCAGATCGGAAGAGCGTCGTGTAGGGAAAGAGTGT 3′
9bp-236B	5′ CTCTTTCCCTACACGACGCTCTTCCGATCTGCAGTCAGCC 3′
10bp-802	5′ AATTGGAGATGGTAAAGATCGGAAGAGCGTCGTGTAGGGAAAGAGTGT 3′
10bp-802B	5′ CTCTTTCCCTACACGACGCTCTTCCGATCTTTACCATCTCC 3′
9bp-237	5′ AATTGGCTCTCTCTAGATCGGAAGAGCGTCGTGTAGGGAAAGAGTGT 3′
9bp-237B	5′ CTCTTTCCCTACACGACGCTCTTCCGATCTAGAGAGAGCC 3′
10bp-803	5′ AATTGGAGATATCGTAGATCGGAAGAGCGTCGTGTAGGGAAAGAGTGT 3′
10bp-803B	5′ CTCTTTCCCTACACGACGCTCTTCCGATCTACGATATCTCC 3′
9bp-238	5′ AATTGGCTCGTTACAGATCGGAAGAGCGTCGTGTAGGGAAAGAGTGT 3′
9bp-238B	5′ CTCTTTCCCTACACGACGCTCTTCCGATCTGTAACGAGCC 3′
10bp-805	5′ AATTGGAGAGCGGTAAGATCGGAAGAGCGTCGTGTAGGGAAAGAGTGT 3′
10bp-805B	5′ CTCTTTCCCTACACGACGCTCTTCCGATCTTACCGCTCTCC 3′
9bp-239	5′ AATTGGCTCAACGGAGATCGGAAGAGCGTCGTGTAGGGAAAGAGTGT 3′
9bp-239B	5′ CTCTTTCCCTACACGACGCTCTTCCGATCTCCGTTGAGCC 3′
10bp-806	5′ AATTGGAGAGCATACAGATCGGAAGAGCGTCGTGTAGGGAAAGAGTGT 3′
10bp-806B	5′ CTCTTTCCCTACACGACGCTCTTCCGATCTGTATGCTCTCC 3′
9bp-240	5′ AATTGGCTATGGTCAGATCGGAAGAGCGTCGTGTAGGGAAAGAGTGT 3′
9bp-240B	5′ CTCTTTCCCTACACGACGCTCTTCCGATCTGACCATAGCC 3′
10bp-808	5′ AATTGGAGACCTCTCAGATCGGAAGAGCGTCGTGTAGGGAAAGAGTGT 3′
10bp-808B	5′ CTCTTTCCCTACACGACGCTCTTCCGATCTGAGAGGTCTCC 3′
9bp-241	5′ AATTGGCGTTGAGGAGATCGGAAGAGCGTCGTGTAGGGAAAGAGTGT 3′
9bp-241B	5′ CTCTTTCCCTACACGACGCTCTTCCGATCTCCTCAACGCC 3′
10bp-812	5′ AATTGGACTCGTAGAAGATCGGAAGAGCGTCGTGTAGGGAAAGAGTGT 3′
10bp-812B	5′ CTCTTTCCCTACACGACGCTCTTCCGATCTTCTACGAGTCC 3′
9bp-242	5′ AATTGGCGTTCCATAGATCGGAAGAGCGTCGTGTAGGGAAAGAGTGT 3′
9bp-242B	5′ CTCTTTCCCTACACGACGCTCTTCCGATCTATGGAACGCC 3′
10bp-817	5′ AATTGGACGCGGTTCAGATCGGAAGAGCGTCGTGTAGGGAAAGAGTGT 3′
10bp-817B	5′ CTCTTTCCCTACACGACGCTCTTCCGATCTGAACCGCGTCC 3′
9bp-243	5′ AATTGGCGTTATTCAGATCGGAAGAGCGTCGTGTAGGGAAAGAGTGT 3′
9bp-243B	5′ CTCTTTCCCTACACGACGCTCTTCCGATCTGAATAACGCC 3′
10bp-819	5′ AATTGGACGAGCCAAAGATCGGAAGAGCGTCGTGTAGGGAAAGAGTGT 3′
10bp-819B	5′ CTCTTTCCCTACACGACGCTCTTCCGATCTTTGGCTCGTCC 3′
9bp-244	5′ AATTGGCGTCGGCCAGATCGGAAGAGCGTCGTGTAGGGAAAGAGTGT 3′
9bp-244B	5′ CTCTTTCCCTACACGACGCTCTTCCGATCTGGCCGACGCC 3′
10bp-822	5′ AATTGGACCAGGATAAGATCGGAAGAGCGTCGTGTAGGGAAAGAGTGT 3′
10bp-822B	5′ CTCTTTCCCTACACGACGCTCTTCCGATCTTATCCTGGTCC 3′
9bp-245	5′ AATTGGCGGCTGATAGATCGGAAGAGCGTCGTGTAGGGAAAGAGTGT 3′
9bp-245B	5′ CTCTTTCCCTACACGACGCTCTTCCGATCTATCAGCCGCC 3′
10bp-824	5′ AATTGGAATTACGACAGATCGGAAGAGCGTCGTGTAGGGAAAGAGTGT 3′
10bp-824B	5′ CTCTTTCCCTACACGACGCTCTTCCGATCTGTCGTAATTCC 3′
9bp-246	5′ AATTGGCGGCATGGAGATCGGAAGAGCGTCGTGTAGGGAAAGAGTGT 3′
9bp-246B	5′ CTCTTTCCCTACACGACGCTCTTCCGATCTCCATGCCGCC 3′
10bp-830	5′ AATTGGAAGGAATCGAGATCGGAAGAGCGTCGTGTAGGGAAAGAGTGT 3′
10bp-830B	5′ CTCTTTCCCTACACGACGCTCTTCCGATCTCGATTCCTTCC 3′
9bp-247	5′ AATTGGCGCGTCTTAGATCGGAAGAGCGTCGTGTAGGGAAAGAGTGT 3′
9bp-247B	5′ CTCTTTCCCTACACGACGCTCTTCCGATCTAAGACGCGCC 3′
10bp-831	5′ AATTGGAAGCTTAGCAGATCGGAAGAGCGTCGTGTAGGGAAAGAGTGT 3′
10bp-831B	5′ CTCTTTCCCTACACGACGCTCTTCCGATCTGCTAAGCTTCC 3′

Supplementary Table 2 - *Continued from previous page*

Oligo Name	Sequence
9bp-248	5′ AATTGGCGCGAGAAAGATCGGAAGAGCGTCGTGTAGGGAAAGAGTGT 3′
9bp-248B	5′ CTCTTTCCCTACACGACGCTCTTCCGATCTTTCTCGCGCC 3′
10bp-832	5′ AATTGGAAGCCAGAGAGATCGGAAGAGCGTCGTGTAGGGAAAGAGTGT 3′
10bp-832B	5′ CTCTTTCCCTACACGACGCTCTTCCGATCTCTCTGGCTTCC 3′
9bp-249	5′ AATTGGCGCATAAGAGATCGGAAGAGCGTCGTGTAGGGAAAGAGTGT 3′
9bp-249B	5′ CTCTTTCCCTACACGACGCTCTTCCGATCTCTTATGCGCC 3′
10bp-833	5′ AATTGGAAGCAGGTAAGATCGGAAGAGCGTCGTGTAGGGAAAGAGTGT 3′
10bp-833B	5′ CTCTTTCCCTACACGACGCTCTTCCGATCTTACCTGCTTCC 3′
9bp-250	5′ AATTGGCGATTCGCAGATCGGAAGAGCGTCGTGTAGGGAAAGAGTGT 3′
9bp-250B	5′ CTCTTTCCCTACACGACGCTCTTCCGATCTGCGAATCGCC 3′
10bp-835	5′ AATTGGAACTATGGAAGATCGGAAGAGCGTCGTGTAGGGAAAGAGTGT 3′
10bp-835B	5′ CTCTTTCCCTACACGACGCTCTTCCGATCTTCCATAGTTCC 3′
9bp-251	5′ AATTGGCGAGGTCGAGATCGGAAGAGCGTCGTGTAGGGAAAGAGTGT 3′
9bp-251B	5′ CTCTTTCCCTACACGACGCTCTTCCGATCTCGACCTCGCC 3′
10bp-837	5′ AATTGGAACGAGAGGAGATCGGAAGAGCGTCGTGTAGGGAAAGAGTGT 3′
10bp-837B	5′ CTCTTTCCCTACACGACGCTCTTCCGATCTCCTCTCGTTCC 3′
9bp-252	5′ AATTGGCGACCAGTAGATCGGAAGAGCGTCGTGTAGGGAAAGAGTGT 3′
9bp-252B	5′ CTCTTTCCCTACACGACGCTCTTCCGATCTACTGGTCGCC 3′
10bp-838	5′ AATTGCTTGGCATAAAGATCGGAAGAGCGTCGTGTAGGGAAAGAGTGT 3′
10bp-838B	5′ CTCTTTCCCTACACGACGCTCTTCCGATCTTTATGCCAAGC 3′
9bp-253	5′ AATTGGCGAACGACAGATCGGAAGAGCGTCGTGTAGGGAAAGAGTGT 3′
9bp-253B	5′ CTCTTTCCCTACACGACGCTCTTCCGATCTGTCGTTCGCC 3′
10bp-839	5′ AATTGCTTGGAGGATAGATCGGAAGAGCGTCGTGTAGGGAAAGAGTGT 3′
10bp-839B	5′ CTCTTTCCCTACACGACGCTCTTCCGATCTATCCTCCAAGC 3′
9bp-254	5′ AATTGGCCTTCGCGAGATCGGAAGAGCGTCGTGTAGGGAAAGAGTGT 3′
9bp-254B	5′ CTCTTTCCCTACACGACGCTCTTCCGATCTCGCGAAGGCC 3′
10bp-840	5′ AATTGCTTGGAACGCAGATCGGAAGAGCGTCGTGTAGGGAAAGAGTGT 3′
10bp-840B	5′ CTCTTTCCCTACACGACGCTCTTCCGATCTGCGTTCCAAGC 3′
9bp-255	5′ AATTGGCCTAGGTTAGATCGGAAGAGCGTCGTGTAGGGAAAGAGTGT 3′
9bp-255B	5′ CTCTTTCCCTACACGACGCTCTTCCGATCTAACCTAGGCC 3′
10bp-841	5′ AATTGCTTGCGCCTCAGATCGGAAGAGCGTCGTGTAGGGAAAGAGTGT 3′
10bp-841B	5′ CTCTTTCCCTACACGACGCTCTTCCGATCTGAGGCGCAAGC 3′
9bp-256	5′ AATTGGCCGGTATGAGATCGGAAGAGCGTCGTGTAGGGAAAGAGTGT 3′
9bp-256B	5′ CTCTTTCCCTACACGACGCTCTTCCGATCTCATACCGGCC 3′
10bp-842	5′ AATTGCTTGCCGCGTAGATCGGAAGAGCGTCGTGTAGGGAAAGAGTGT 3′
10bp-842B	5′ CTCTTTCCCTACACGACGCTCTTCCGATCTACGCGGCAAGC 3′
9bp-257	5′ AATTGGCCGGAGGCAGATCGGAAGAGCGTCGTGTAGGGAAAGAGTGT 3′
9bp-257B	5′ CTCTTTCCCTACACGACGCTCTTCCGATCTGCCTCCGGCC 3′
10bp-843	5′ AATTGCTTGCCAGCGAGATCGGAAGAGCGTCGTGTAGGGAAAGAGTGT 3′
10bp-843B	5′ CTCTTTCCCTACACGACGCTCTTCCGATCTCGCTGGCAAGC 3′
9bp-258	5′ AATTGGCCGATCAAAGATCGGAAGAGCGTCGTGTAGGGAAAGAGTGT 3′
9bp-258B	5′ CTCTTTCCCTACACGACGCTCTTCCGATCTTTGATCGGCC 3′
10bp-844	5′ AATTGCTTGAGATCCAGATCGGAAGAGCGTCGTGTAGGGAAAGAGTGT 3′
10bp-844B	5′ CTCTTTCCCTACACGACGCTCTTCCGATCTGGATCTCAAGC 3′
9bp-259	5′ AATTGGCCAGGAACAGATCGGAAGAGCGTCGTGTAGGGAAAGAGTGT 3′
9bp-259B	5′ CTCTTTCCCTACACGACGCTCTTCCGATCTGTTCCTGGCC 3′
10bp-845	5′ AATTGCTTGACCTTGAGATCGGAAGAGCGTCGTGTAGGGAAAGAGTGT 3′
10bp-845B	5′ CTCTTTCCCTACACGACGCTCTTCCGATCTCAAGGTCAAGC 3′
9bp-260	5′ AATTGGCATGCCTGAGATCGGAAGAGCGTCGTGTAGGGAAAGAGTGT 3′
9bp-260B	5′ CTCTTTCCCTACACGACGCTCTTCCGATCTCAGGCATGCC 3′
10bp-846	5′ AATTGCTTCTGATAGAGATCGGAAGAGCGTCGTGTAGGGAAAGAGTGT 3′
10bp-846B	5′ CTCTTTCCCTACACGACGCTCTTCCGATCTCTATCAGAAGC 3′
9bp-261	5′ AATTGGCATCGTAGAGATCGGAAGAGCGTCGTGTAGGGAAAGAGTGT 3′
9bp-261B	5′ CTCTTTCCCTACACGACGCTCTTCCGATCTCTACGATGCC 3′
10bp-847	5′ AATTGCTTCGCTACCAGATCGGAAGAGCGTCGTGTAGGGAAAGAGTGT 3′
10bp-847B	5′ CTCTTTCCCTACACGACGCTCTTCCGATCTGGTAGCGAAGC 3′
9bp-262	5′ AATTGGCAGTCGGTAGATCGGAAGAGCGTCGTGTAGGGAAAGAGTGT 3′
9bp-262B	5′ CTCTTTCCCTACACGACGCTCTTCCGATCTACCGACTGCC 3′
10bp-848	5′ AATTGCTTCCGGACTAGATCGGAAGAGCGTCGTGTAGGGAAAGAGTGT 3′
10bp-848B	5′ CTCTTTCCCTACACGACGCTCTTCCGATCTAGTCCGGAAGC 3′
9bp-263	5′ AATTGGCAGCAATTAGATCGGAAGAGCGTCGTGTAGGGAAAGAGTGT 3′
9bp-263B	5′ CTCTTTCCCTACACGACGCTCTTCCGATCTAATTGCTGCC 3′

Supplementary Table 2 - *Continued from previous page*

**Oligo Name**	**Sequence**
10bp-849	5′ AATTGCTTCATTGGTAGATCGGAAGAGCGTCGTGTAGGGAAAGAGTGT 3′
10bp-849B	5′ CTCTTTCCCTACACGACGCTCTTCCGATCTACCAATGAAGC 3′
9bp-264	5′ AATTGGCAATAACCAGATCGGAAGAGCGTCGTGTAGGGAAAGAGTGT 3′
9bp-264B	5′ CTCTTTCCCTACACGACGCTCTTCCGATCTGGTTATTGCC 3′
10bp-850	5′ AATTGCTTCATCTACAGATCGGAAGAGCGTCGTGTAGGGAAAGAGTGT 3′
10bp-850B	5′ CTCTTTCCCTACACGACGCTCTTCCGATCTGTAGATGAAGC 3′
9bp-265	5′ AATTGGCAAGCGCAAGATCGGAAGAGCGTCGTGTAGGGAAAGAGTGT 3′
9bp-265B	5′ CTCTTTCCCTACACGACGCTCTTCCGATCTTGCGCTTGCC 3′
9bp-266	5′ AATTGGATCTAAGTAGATCGGAAGAGCGTCGTGTAGGGAAAGAGTGT 3′
9bp-266B	5′ CTCTTTCCCTACACGACGCTCTTCCGATCTACTTAGATCC 3′
10bp-852	5′ AATTGCTTAGCCGACAGATCGGAAGAGCGTCGTGTAGGGAAAGAGTGT 3′
10bp-852B	5′ CTCTTTCCCTACACGACGCTCTTCCGATCTGTCGGCTAAGC 3′
9bp-267	5′ AATTGGATATGCAGAGATCGGAAGAGCGTCGTGTAGGGAAAGAGTGT 3′
9bp-267B	5′ CTCTTTCCCTACACGACGCTCTTCCGATCTCTGCATATCC 3′
10bp-853	5′ AATTGCTTACTCCATAGATCGGAAGAGCGTCGTGTAGGGAAAGAGTGT 3′
10bp-853B	5′ CTCTTTCCCTACACGACGCTCTTCCGATCTATGGAGTAAGC 3′
9bp-268	5′ AATTGGATACTAGAAGATCGGAAGAGCGTCGTGTAGGGAAAGAGTGT 3′
9bp-268B	5′ CTCTTTCCCTACACGACGCTCTTCCGATCTTCTAGTATCC 3′
10bp-854	5′ AATTGCTTACGCTCGAGATCGGAAGAGCGTCGTGTAGGGAAAGAGTGT 3′
10bp-854B	5′ CTCTTTCCCTACACGACGCTCTTCCGATCTCGAGCGTAAGC 3′
9bp-269	5′ AATTGGAGTCGCGTAGATCGGAAGAGCGTCGTGTAGGGAAAGAGTGT 3′
9bp-269B	5′ CTCTTTCCCTACACGACGCTCTTCCGATCTACGCGACTCC 3′
10bp-856	5′ AATTGCTGGCTAATTAGATCGGAAGAGCGTCGTGTAGGGAAAGAGTGT 3′
10bp-856B	5′ CTCTTTCCCTACACGACGCTCTTCCGATCTAATTAGCCAGC 3′
9bp-270	5′ AATTGGAGTCCATCAGATCGGAAGAGCGTCGTGTAGGGAAAGAGTGT 3′
9bp-270B	5′ CTCTTTCCCTACACGACGCTCTTCCGATCTGATGGACTCC 3′
10bp-857	5′ AATTGCTGGCATAGGAGATCGGAAGAGCGTCGTGTAGGGAAAGAGTGT 3′
10bp-857B	5′ CTCTTTCCCTACACGACGCTCTTCCGATCTCCTATGCCAGC 3′
9bp-271	5′ AATTGGAGTATTGCAGATCGGAAGAGCGTCGTGTAGGGAAAGAGTGT 3′
9bp-271B	5′ CTCTTTCCCTACACGACGCTCTTCCGATCTGCAATACTCC 3′
10bp-858	5′ AATTGCTGGATGCGCAGATCGGAAGAGCGTCGTGTAGGGAAAGAGTGT 3′
10bp-858B	5′ CTCTTTCCCTACACGACGCTCTTCCGATCTGCGCATCCAGC 3′
9bp-272	5′ AATTGGAGTACGCTAGATCGGAAGAGCGTCGTGTAGGGAAAGAGTGT 3′
9bp-272B	5′ CTCTTTCCCTACACGACGCTCTTCCGATCTAGCGTACTCC 3′
10bp-861	5′ AATTGCTGCTATGATAGATCGGAAGAGCGTCGTGTAGGGAAAGAGTGT 3′
10bp-861B	5′ CTCTTTCCCTACACGACGCTCTTCCGATCTATCATAGCAGC 3′
10bp-866	5′ AATTGCTGCAACGTCAGATCGGAAGAGCGTCGTGTAGGGAAAGAGTGT 3′
10bp-866B	5′ CTCTTTCCCTACACGACGCTCTTCCGATCTGACGTTGCAGC 3′
9bp-274	5′ AATTGGAGGTCTACAGATCGGAAGAGCGTCGTGTAGGGAAAGAGTGT 3′
9bp-274B	5′ CTCTTTCCCTACACGACGCTCTTCCGATCTGTAGACCTCC 3′
10bp-867	5′ AATTGCTGATTACTCAGATCGGAAGAGCGTCGTGTAGGGAAAGAGTGT 3′
10bp-867B	5′ CTCTTTCCCTACACGACGCTCTTCCGATCTGAGTAATCAGC 3′
9bp-275	5′ AATTGGAGCTATCGAGATCGGAAGAGCGTCGTGTAGGGAAAGAGTGT 3′
9bp-275B	5′ CTCTTTCCCTACACGACGCTCTTCCGATCTCGATAGCTCC 3′
10bp-868	5′ AATTGCTGAGGCTGCAGATCGGAAGAGCGTCGTGTAGGGAAAGAGTGT 3′
10bp-868B	5′ CTCTTTCCCTACACGACGCTCTTCCGATCTGCAGCCTCAGC 3′
9bp-276	5′ AATTGGAGCGGTATAGATCGGAAGAGCGTCGTGTAGGGAAAGAGTGT 3′
9bp-276B	5′ CTCTTTCCCTACACGACGCTCTTCCGATCTATACCGCTCC 3′
10bp-873	5′ AATTGCTCTGCTCGCAGATCGGAAGAGCGTCGTGTAGGGAAAGAGTGT 3′
10bp-873B	5′ CTCTTTCCCTACACGACGCTCTTCCGATCTGCGAGCAGAGC 3′
9bp-277	5′ AATTGGAGCGCCGCAGATCGGAAGAGCGTCGTGTAGGGAAAGAGTGT 3′
9bp-277B	5′ CTCTTTCCCTACACGACGCTCTTCCGATCTGCGGCGCTCC 3′
10bp-874	5′ AATTGCTCTCTCAAGAGATCGGAAGAGCGTCGTGTAGGGAAAGAGTGT 3′
10bp-874B	5′ CTCTTTCCCTACACGACGCTCTTCCGATCTCTTGAGAGAGC 3′
9bp-278	5′ AATTGGAGCCAGTTAGATCGGAAGAGCGTCGTGTAGGGAAAGAGTGT 3′
9bp-278B	5′ CTCTTTCCCTACACGACGCTCTTCCGATCTAACTGGCTCC 3′
10bp-876	5′ AATTGCTCTACTATGAGATCGGAAGAGCGTCGTGTAGGGAAAGAGTGT 3′
10bp-876B	5′ CTCTTTCCCTACACGACGCTCTTCCGATCTCATAGTAGAGC 3′
9bp-279	5′ AATTGGACTGCTGGAGATCGGAAGAGCGTCGTGTAGGGAAAGAGTGT 3′
9bp-279B	5′ CTCTTTCCCTACACGACGCTCTTCCGATCTCCAGCAGTCC 3′
10bp-887	5′ AATTGCTCATCAGTTAGATCGGAAGAGCGTCGTGTAGGGAAAGAGTGT 3′
10bp-887B	5′ CTCTTTCCCTACACGACGCTCTTCCGATCTAACTGATGAGC 3′

Supplementary Table 2 - *Continued from previous page*

**Oligo Name**	**Sequence**
9bp-280	5′ AATTGGACTCTGAGAGATCGGAAGAGCGTCGTGTAGGGAAAGAGTGT 3′
9bp-280B	5′ CTCTTTCCCTACACGACGCTCTTCCGATCTCTCAGAGTCC 3′
10bp-889	5′ AATTGCTCAATATCAAGATCGGAAGAGCGTCGTGTAGGGAAAGAGTGT 3′
10bp-889B	5′ CTCTTTCCCTACACGACGCTCTTCCGATCTTGATATTGAGC 3′
9bp-281	5′ AATTGGACTCATCCAGATCGGAAGAGCGTCGTGTAGGGAAAGAGTGT 3′
9bp-281B	5′ CTCTTTCCCTACACGACGCTCTTCCGATCTGGATGAGTCC 3′
10bp-892	5′ AATTGCTATCCGGAAAGATCGGAAGAGCGTCGTGTAGGGAAAGAGTGT 3′
10bp-892B	5′ CTCTTTCCCTACACGACGCTCTTCCGATCTTTCCGGATAGC 3′
9bp-282	5′ AATTGGACCTGGCCAGATCGGAAGAGCGTCGTGTAGGGAAAGAGTGT 3′
9bp-282B	5′ CTCTTTCCCTACACGACGCTCTTCCGATCTGGCCAGGTCC 3′
10bp-895	5′ AATTGCTAGTTACGGAGATCGGAAGAGCGTCGTGTAGGGAAAGAGTGT 3′
10bp-895B	5′ CTCTTTCCCTACACGACGCTCTTCCGATCTCCGTAACTAGC 3′
9bp-283	5′ AATTGGACCATCCTAGATCGGAAGAGCGTCGTGTAGGGAAAGAGTGT 3′
9bp-283B	5′ CTCTTTCCCTACACGACGCTCTTCCGATCTAGGATGGTCC 3′
10bp-899	5′ AATTGCTAGACCAGAAGATCGGAAGAGCGTCGTGTAGGGAAAGAGTGT 3′
10bp-899B	5′ CTCTTTCCCTACACGACGCTCTTCCGATCTTCTGGTCTAGC 3′
9bp-284	5′ AATTGGAATTGCTCAGATCGGAAGAGCGTCGTGTAGGGAAAGAGTGT 3′
9bp-284B	5′ CTCTTTCCCTACACGACGCTCTTCCGATCTGAGCAATTCC 3′
10bp-902	5′ AATTGCTACGCTTGGAGATCGGAAGAGCGTCGTGTAGGGAAAGAGTGT 3′
10bp-902B	5′ CTCTTTCCCTACACGACGCTCTTCCGATCTCCAAGCGTAGC 3′
9bp-285	5′ AATTGGAATGCGACAGATCGGAAGAGCGTCGTGTAGGGAAAGAGTGT 3′
9bp-285B	5′ CTCTTTCCCTACACGACGCTCTTCCGATCTGTCGCATTCC 3′
10bp-903	5′ AATTGCTACCTCCTGAGATCGGAAGAGCGTCGTGTAGGGAAAGAGTGT 3′
10bp-903B	5′ CTCTTTCCCTACACGACGCTCTTCCGATCTCAGGAGGTAGC 3′
9bp-286	5′ AATTGGAAGACCATAGATCGGAAGAGCGTCGTGTAGGGAAAGAGTGT 3′
9bp-286B	5′ CTCTTTCCCTACACGACGCTCTTCCGATCTATGGTCTTCC 3′
10bp-906	5′ AATTGCTAATATATGAGATCGGAAGAGCGTCGTGTAGGGAAAGAGTGT 3′
10bp-906B	5′ CTCTTTCCCTACACGACGCTCTTCCGATCTCATATATTAGC 3′
9bp-287	5′ AATTGGAACGAATGAGATCGGAAGAGCGTCGTGTAGGGAAAGAGTGT 3′
9bp-287B	5′ CTCTTTCCCTACACGACGCTCTTCCGATCTCATTCGTTCC 3′
10bp-908	5′ AATTGCTAAGAGCGAAGATCGGAAGAGCGTCGTGTAGGGAAAGAGTGT 3′
10bp-908B	5′ CTCTTTCCCTACACGACGCTCTTCCGATCTTCGCTCTTAGC 3′
9bp-288	5′ AATTGCTTGACTATAGATCGGAAGAGCGTCGTGTAGGGAAAGAGTGT 3′
9bp-288B	5′ CTCTTTCCCTACACGACGCTCTTCCGATCTATAGTCAAGC 3′
10bp-909	5′ AATTGCGTTGGCGCGAGATCGGAAGAGCGTCGTGTAGGGAAAGAGTGT 3′
10bp-909B	5′ CTCTTTCCCTACACGACGCTCTTCCGATCTCGCGCCAACGC 3′
10bp-910	5′ AATTGCGTTGCATGCAGATCGGAAGAGCGTCGTGTAGGGAAAGAGTGT 3′
10bp-910B	5′ CTCTTTCCCTACACGACGCTCTTCCGATCTGCATGCAACGC 3′
9bp-290	5′ AATTGCTTAGATACAGATCGGAAGAGCGTCGTGTAGGGAAAGAGTGT 3′
9bp-290B	5′ CTCTTTCCCTACACGACGCTCTTCCGATCTGTATCTAAGC 3′
10bp-911	5′ AATTGCGTTCGCAACAGATCGGAAGAGCGTCGTGTAGGGAAAGAGTGT 3′
10bp-911B	5′ CTCTTTCCCTACACGACGCTCTTCCGATCTGTTGCGAACGC 3′
9bp-291	5′ AATTGCTTACGATGAGATCGGAAGAGCGTCGTGTAGGGAAAGAGTGT 3′
9bp-291B	5′ CTCTTTCCCTACACGACGCTCTTCCGATCTCATCGTAAGC 3′
10bp-912	5′ AATTGCGTTCCGCTCAGATCGGAAGAGCGTCGTGTAGGGAAAGAGTGT 3′
10bp-912B	5′ CTCTTTCCCTACACGACGCTCTTCCGATCTGAGCGGAACGC 3′
9bp-292	5′ AATTGCTTAACGCGAGATCGGAAGAGCGTCGTGTAGGGAAAGAGTGT 3′
9bp-292B	5′ CTCTTTCCCTACACGACGCTCTTCCGATCTCGCGTTAAGC 3′
10bp-925	5′ AATTGCGTAGGTACTAGATCGGAAGAGCGTCGTGTAGGGAAAGAGTGT 3′
10bp-925B	5′ CTCTTTCCCTACACGACGCTCTTCCGATCTAGTACCTACGC 3′
9bp-293	5′ AATTGCTGGATATCAGATCGGAAGAGCGTCGTGTAGGGAAAGAGTGT 3′
9bp-293B	5′ CTCTTTCCCTACACGACGCTCTTCCGATCTGATATCCAGC 3′
10bp-926	5′ AATTGCGTACTAGGCAGATCGGAAGAGCGTCGTGTAGGGAAAGAGTGT 3′
10bp-926B	5′ CTCTTTCCCTACACGACGCTCTTCCGATCTGCCTAGTACGC 3′
9bp-294	5′ AATTGCTGCCATCTAGATCGGAAGAGCGTCGTGTAGGGAAAGAGTGT 3′
9bp-294B	5′ CTCTTTCCCTACACGACGCTCTTCCGATCTAGATGGCAGC 3′
10bp-934	5′ AATTGCGGCTGCTTGAGATCGGAAGAGCGTCGTGTAGGGAAAGAGTGT 3′
10bp-934B	5′ CTCTTTCCCTACACGACGCTCTTCCGATCTCAAGCAGCCGC 3′
9bp-295	5′ AATTGCTGATTGAGAGATCGGAAGAGCGTCGTGTAGGGAAAGAGTGT 3′
9bp-295B	5′ CTCTTTCCCTACACGACGCTCTTCCGATCTCTCAATCAGC 3′
10bp-936	5′ AATTGCGGCCATACCAGATCGGAAGAGCGTCGTGTAGGGAAAGAGTGT 3′
10bp-936B	5′ CTCTTTCCCTACACGACGCTCTTCCGATCTGGTATGGCCGC 3′

Supplementary Table 2 - *Continued from previous page*

**Oligo Name**	**Sequence**
9bp-296	5′ AATTGCTGAAGCATAGATCGGAAGAGCGTCGTGTAGGGAAAGAGTGT 3′
9bp-296B	5′ CTCTTTCCCTACACGACGCTCTTCCGATCTATGCTTCAGC 3′
10bp-937	5′ AATTGCGGCATTAATAGATCGGAAGAGCGTCGTGTAGGGAAAGAGTGT 3′
10bp-937B	5′ CTCTTTCCCTACACGACGCTCTTCCGATCTATTAATGCCGC 3′
9bp-297	5′ AATTGCTCTACCGGAGATCGGAAGAGCGTCGTGTAGGGAAAGAGTGT 3′
9bp-297B	5′ CTCTTTCCCTACACGACGCTCTTCCGATCTCCGGTAGAGC 3′
10bp-938	5′ AATTGCGGCATCCGGAGATCGGAAGAGCGTCGTGTAGGGAAAGAGTGT 3′
10bp-938B	5′ CTCTTTCCCTACACGACGCTCTTCCGATCTCCGGATGCCGC 3′
9bp-298	5′ AATTGCTCTAAGATAGATCGGAAGAGCGTCGTGTAGGGAAAGAGTGT 3′
9bp-298B	5′ CTCTTTCCCTACACGACGCTCTTCCGATCTATCTTAGAGC 3′
10bp-939	5′ AATTGCGGAGCGCGCAGATCGGAAGAGCGTCGTGTAGGGAAAGAGTGT 3′
10bp-939B	5′ CTCTTTCCCTACACGACGCTCTTCCGATCTGCGCGCTCCGC 3′
10bp-942	5′ AATTGCGGAGAGTTGAGATCGGAAGAGCGTCGTGTAGGGAAAGAGTGT 3′
10bp-942B	5′ CTCTTTCCCTACACGACGCTCTTCCGATCTCAACTCTCCGC 3′
9bp-300	5′ AATTGCTCCTGCGCAGATCGGAAGAGCGTCGTGTAGGGAAAGAGTGT 3′
9bp-300B	5′ CTCTTTCCCTACACGACGCTCTTCCGATCTGCGCAGGAGC 3′
10bp-943	5′ AATTGCGGACTCATCAGATCGGAAGAGCGTCGTGTAGGGAAAGAGTGT 3′
10bp-943B	5′ CTCTTTCCCTACACGACGCTCTTCCGATCTGATGAGTCCGC 3′
9bp-301	5′ AATTGCTCCGTCTGAGATCGGAAGAGCGTCGTGTAGGGAAAGAGTGT 3′
9bp-301B	5′ CTCTTTCCCTACACGACGCTCTTCCGATCTCAGACGGAGC 3′
10bp-946	5′ AATTGCGGAACCGTAAGATCGGAAGAGCGTCGTGTAGGGAAAGAGTGT 3′
10bp-946B	5′ CTCTTTCCCTACACGACGCTCTTCCGATCTTACGGTTCCGC 3′
9bp-302	5′ AATTGCTCAATAGTAGATCGGAAGAGCGTCGTGTAGGGAAAGAGTGT 3′
9bp-302B	5′ CTCTTTCCCTACACGACGCTCTTCCGATCTACTATTGAGC 3′
10bp-947	5′ AATTGCGCTTGACCTAGATCGGAAGAGCGTCGTGTAGGGAAAGAGTGT 3′
10bp-947B	5′ CTCTTTCCCTACACGACGCTCTTCCGATCTAGGTCAAGCGC 3′
9bp-303	5′ AATTGCTATGCAATAGATCGGAAGAGCGTCGTGTAGGGAAAGAGTGT 3′
9bp-303B	5′ CTCTTTCCCTACACGACGCTCTTCCGATCTATTGCATAGC 3′
10bp-953	5′ AATTGCGCGGAGCTTAGATCGGAAGAGCGTCGTGTAGGGAAAGAGTGT 3′
10bp-953B	5′ CTCTTTCCCTACACGACGCTCTTCCGATCTAAGCTCCGCGC 3′
9bp-304	5′ AATTGCTAGCGGCGAGATCGGAAGAGCGTCGTGTAGGGAAAGAGTGT 3′
9bp-304B	5′ CTCTTTCCCTACACGACGCTCTTCCGATCTCGCCGCTAGC 3′
10bp-955	5′ AATTGCGCGAGTCTGAGATCGGAAGAGCGTCGTGTAGGGAAAGAGTGT 3′
10bp-955B	5′ CTCTTTCCCTACACGACGCTCTTCCGATCTCAGACTCGCGC 3′
9bp-305	5′ AATTGCTACGCGTAAGATCGGAAGAGCGTCGTGTAGGGAAAGAGTGT 3′
9bp-305B	5′ CTCTTTCCCTACACGACGCTCTTCCGATCTTACGCGTAGC 3′
10bp-956	5′ AATTGCGCGAATTCCAGATCGGAAGAGCGTCGTGTAGGGAAAGAGTGT 3′
10bp-956B	5′ CTCTTTCCCTACACGACGCTCTTCCGATCTGGAATTCGCGC 3′
9bp-306	5′ AATTGCTACCGAACAGATCGGAAGAGCGTCGTGTAGGGAAAGAGTGT 3′
9bp-306B	5′ CTCTTTCCCTACACGACGCTCTTCCGATCTGTTCGGTAGC 3′
10bp-957	5′ AATTGCGCGAACGAAAGATCGGAAGAGCGTCGTGTAGGGAAAGAGTGT 3′
10bp-957B	5′ CTCTTTCCCTACACGACGCTCTTCCGATCTTTCGTTCGCGC 3′
9bp-307	5′ AATTGCTAATCTGAAGATCGGAAGAGCGTCGTGTAGGGAAAGAGTGT 3′
9bp-307B	5′ CTCTTTCCCTACACGACGCTCTTCCGATCTTCAGATTAGC 3′
10bp-959	5′ AATTGCGCCAGACGCAGATCGGAAGAGCGTCGTGTAGGGAAAGAGTGT 3′
10bp-959B	5′ CTCTTTCCCTACACGACGCTCTTCCGATCTGCGTCTGGCGC 3′
9bp-308	5′ AATTGCTAATACCGAGATCGGAAGAGCGTCGTGTAGGGAAAGAGTGT 3′
9bp-308B	5′ CTCTTTCCCTACACGACGCTCTTCCGATCTCGGTATTAGC 3′
10bp-960	5′ AATTGCGCCAATAGGAGATCGGAAGAGCGTCGTGTAGGGAAAGAGTGT 3′
10bp-960B	5′ CTCTTTCCCTACACGACGCTCTTCCGATCTCCTATTGGCGC 3′
9bp-309	5′ AATTGCGTTGCCGCAGATCGGAAGAGCGTCGTGTAGGGAAAGAGTGT 3′
9bp-309B	5′ CTCTTTCCCTACACGACGCTCTTCCGATCTGCGGCAACGC 3′
10bp-964	5′ AATTGCGCAGAGACCAGATCGGAAGAGCGTCGTGTAGGGAAAGAGTGT 3′
10bp-964B	5′ CTCTTTCCCTACACGACGCTCTTCCGATCTGGTCTCTGCGC 3′
9bp-310	5′ AATTGCGTTAGAAGAGATCGGAAGAGCGTCGTGTAGGGAAAGAGTGT 3′
9bp-310B	5′ CTCTTTCCCTACACGACGCTCTTCCGATCTCTTCTAACGC 3′
10bp-965	5′ AATTGCGCAAGTAACAGATCGGAAGAGCGTCGTGTAGGGAAAGAGTGT 3′
10bp-965B	5′ CTCTTTCCCTACACGACGCTCTTCCGATCTGTTACTTGCGC 3′
9bp-311	5′ AATTGCGTCTCCATAGATCGGAAGAGCGTCGTGTAGGGAAAGAGTGT 3′
9bp-311B	5′ CTCTTTCCCTACACGACGCTCTTCCGATCTATGGAGACGC 3′
10bp-966	5′ AATTGCGCAACGATTAGATCGGAAGAGCGTCGTGTAGGGAAAGAGTGT 3′
10bp-966B	5′ CTCTTTCCCTACACGACGCTCTTCCGATCTAATCGTTGCGC 3′

Supplementary Table 2 - *Continued from previous page*

**Oligo Name**	**Sequence**
9bp-312	5′ AATTGCGTCTATCCAGATCGGAAGAGCGTCGTGTAGGGAAAGAGTGT 3′
9bp-312B	5′ CTCTTTCCCTACACGACGCTCTTCCGATCTGGATAGACGC 3′
10bp-968	5′ AATTGCGATATGATGAGATCGGAAGAGCGTCGTGTAGGGAAAGAGTGT 3′
10bp-968B	5′ CTCTTTCCCTACACGACGCTCTTCCGATCTCATCATATCGC 3′
9bp-313	5′ AATTGCGTCCTGAAAGATCGGAAGAGCGTCGTGTAGGGAAAGAGTGT 3′
9bp-313B	5′ CTCTTTCCCTACACGACGCTCTTCCGATCTTTCAGGACGC 3′
10bp-969	5′ AATTGCGAGTCCGTTAGATCGGAAGAGCGTCGTGTAGGGAAAGAGTGT 3′
10bp-969B	5′ CTCTTTCCCTACACGACGCTCTTCCGATCTAACGGACTCGC 3′
9bp-314	5′ AATTGCGTCATACTAGATCGGAAGAGCGTCGTGTAGGGAAAGAGTGT 3′
9bp-314B	5′ CTCTTTCCCTACACGACGCTCTTCCGATCTAGTATGACGC 3′
10bp-970	5′ AATTGCGAGCTCGCGAGATCGGAAGAGCGTCGTGTAGGGAAAGAGTGT 3′
10bp-970B	5′ CTCTTTCCCTACACGACGCTCTTCCGATCTCGCGAGCTCGC 3′
9bp-315	5′ AATTGCGTACGTATAGATCGGAAGAGCGTCGTGTAGGGAAAGAGTGT 3′
9bp-315B	5′ CTCTTTCCCTACACGACGCTCTTCCGATCTATACGTACGC 3′
10bp-972	5′ AATTGCGAGCAATAGAGATCGGAAGAGCGTCGTGTAGGGAAAGAGTGT 3′
10bp-972B	5′ CTCTTTCCCTACACGACGCTCTTCCGATCTCTATTGCTCGC 3′
9bp-316	5′ AATTGCGGTTCTCGAGATCGGAAGAGCGTCGTGTAGGGAAAGAGTGT 3′
9bp-316B	5′ CTCTTTCCCTACACGACGCTCTTCCGATCTCGAGAACCGC 3′
10bp-973	5′ AATTGCGAGAGGCCTAGATCGGAAGAGCGTCGTGTAGGGAAAGAGTGT 3′
10bp-973B	5′ CTCTTTCCCTACACGACGCTCTTCCGATCTAGGCCTCTCGC 3′
9bp-317	5′ AATTGCGGTCTATTAGATCGGAAGAGCGTCGTGTAGGGAAAGAGTGT 3′
9bp-317B	5′ CTCTTTCCCTACACGACGCTCTTCCGATCTAATAGACCGC 3′
10bp-976	5′ AATTGCGACTGCGCAAGATCGGAAGAGCGTCGTGTAGGGAAAGAGTGT 3′
10bp-976B	5′ CTCTTTCCCTACACGACGCTCTTCCGATCTTGCGCAGTCGC 3′
9bp-318	5′ AATTGCGGTCATGCAGATCGGAAGAGCGTCGTGTAGGGAAAGAGTGT 3′
9bp-318B	5′ CTCTTTCCCTACACGACGCTCTTCCGATCTGCATGACCGC 3′
10bp-977	5′ AATTGCGACTCGATAAGATCGGAAGAGCGTCGTGTAGGGAAAGAGTGT 3′
10bp-977B	5′ CTCTTTCCCTACACGACGCTCTTCCGATCTTATCGAGTCGC 3′
9bp-319	5′ AATTGCGGTACGTCAGATCGGAAGAGCGTCGTGTAGGGAAAGAGTGT 3′
9bp-319B	5′ CTCTTTCCCTACACGACGCTCTTCCGATCTGACGTACCGC 3′
10bp-978	5′ AATTGCGACTATCAGAGATCGGAAGAGCGTCGTGTAGGGAAAGAGTGT 3′
10bp-978B	5′ CTCTTTCCCTACACGACGCTCTTCCGATCTCTGATAGTCGC 3′
9bp-320	5′ AATTGCGGCGATTGAGATCGGAAGAGCGTCGTGTAGGGAAAGAGTGT 3′
9bp-320B	5′ CTCTTTCCCTACACGACGCTCTTCCGATCTCAATCGCCGC 3′
10bp-979	5′ AATTGCGACCTCTGCAGATCGGAAGAGCGTCGTGTAGGGAAAGAGTGT 3′
10bp-979B	5′ CTCTTTCCCTACACGACGCTCTTCCGATCTGCAGAGGTCGC 3′
9bp-321	5′ AATTGCGGATTCCTAGATCGGAAGAGCGTCGTGTAGGGAAAGAGTGT 3′
9bp-321B	5′ CTCTTTCCCTACACGACGCTCTTCCGATCTAGGAATCCGC 3′
10bp-980	5′ AATTGCGAATAGCTCAGATCGGAAGAGCGTCGTGTAGGGAAAGAGTGT 3′
10bp-980B	5′ CTCTTTCCCTACACGACGCTCTTCCGATCTGAGCTATTCGC 3′
9bp-322	5′ AATTGCGGAGCCAGAGATCGGAAGAGCGTCGTGTAGGGAAAGAGTGT 3′
9bp-322B	5′ CTCTTTCCCTACACGACGCTCTTCCGATCTCTGGCTCCGC 3′
10bp-983	5′ AATTGCCTTGCAGCTAGATCGGAAGAGCGTCGTGTAGGGAAAGAGTGT 3′
10bp-983B	5′ CTCTTTCCCTACACGACGCTCTTCCGATCTAGCTGCAAGGC 3′
9bp-323	5′ AATTGCGGAAGTCCAGATCGGAAGAGCGTCGTGTAGGGAAAGAGTGT 3′
9bp-323B	5′ CTCTTTCCCTACACGACGCTCTTCCGATCTGGACTTCCGC 3′
10bp-984	5′ AATTGCCTTGAGCCGAGATCGGAAGAGCGTCGTGTAGGGAAAGAGTGT 3′
10bp-984B	5′ CTCTTTCCCTACACGACGCTCTTCCGATCTCGGCTCAAGGC 3′
9bp-324	5′ AATTGCGCTATGCGAGATCGGAAGAGCGTCGTGTAGGGAAAGAGTGT 3′
9bp-324B	5′ CTCTTTCCCTACACGACGCTCTTCCGATCTCGCATAGCGC 3′
10bp-985	5′ AATTGCCTTCTATCGAGATCGGAAGAGCGTCGTGTAGGGAAAGAGTGT 3′
10bp-985B	5′ CTCTTTCCCTACACGACGCTCTTCCGATCTCGATAGAAGGC 3′
9bp-325	5′ AATTGCGCGGCAACAGATCGGAAGAGCGTCGTGTAGGGAAAGAGTGT 3′
9bp-325B	5′ CTCTTTCCCTACACGACGCTCTTCCGATCTGTTGCCGCGC 3′
10bp-986	5′ AATTGCCTTATCCAAAGATCGGAAGAGCGTCGTGTAGGGAAAGAGTGT 3′
10bp-986B	5′ CTCTTTCCCTACACGACGCTCTTCCGATCTTTGGATAAGGC 3′
9bp-326	5′ AATTGCGCGCAGGTAGATCGGAAGAGCGTCGTGTAGGGAAAGAGTGT 3′
9bp-326B	5′ CTCTTTCCCTACACGACGCTCTTCCGATCTACCTGCGCGC 3′
10bp-987	5′ AATTGCCTTAATTGCAGATCGGAAGAGCGTCGTGTAGGGAAAGAGTGT 3′
10bp-987B	5′ CTCTTTCCCTACACGACGCTCTTCCGATCTGCAATTAAGGC 3′
9bp-327	5′ AATTGCGCGAGCTGAGATCGGAAGAGCGTCGTGTAGGGAAAGAGTGT 3′
9bp-327B	5′ CTCTTTCCCTACACGACGCTCTTCCGATCTCAGCTCGCGC 3′

Supplementary Table 2 - *Continued from previous page*

**Oligo Name**	**Sequence**
10bp-990	5′ AATTGCCTGCTTGGAAGATCGGAAGAGCGTCGTGTAGGGAAAGAGTGT 3′
10bp-990B	5′ CTCTTTCCCTACACGACGCTCTTCCGATCTTCCAAGCAGGC 3′
9bp-328	5′ AATTGCGCATCGCCAGATCGGAAGAGCGTCGTGTAGGGAAAGAGTGT 3′
9bp-328B	5′ CTCTTTCCCTACACGACGCTCTTCCGATCTGGCGATGCGC 3′
10bp-994	5′ AATTGCCTCCATTCAAGATCGGAAGAGCGTCGTGTAGGGAAAGAGTGT 3′
10bp-994B	5′ CTCTTTCCCTACACGACGCTCTTCCGATCTTGAATGGAGGC 3′
9bp-329	5′ AATTGCGATTAGAGAGATCGGAAGAGCGTCGTGTAGGGAAAGAGTGT 3′
9bp-329B	5′ CTCTTTCCCTACACGACGCTCTTCCGATCTCTCTAATCGC 3′

Supplementary Table 3: Mapping summary metrics across reference genomes. We found close correspondence amongst bioinformatic analyses when using any one of the three reference genomes listed above. As a result, our final conclusions in the main text focuses on reads mapped to the pan-genome reference as we believe this captures the range in genetic variation in our system.

	**Scaffold 1260**	**Concatenated**	**Pan-genome**
**# of reads (total)**	12.32 mil	7.62 mil	11.03 mil
**# of reads (>80 bp)**	10.01 mil (82.15%)	6.32 mil (82.92%)	8.75 mil (79.27%)
**avg. # of reads mapped to Wolbachia**	19.50%	21.35%	19.37%
**# of infected individuals**	2,101 (88.38%)	2,114 (88.94%)	2,117 (89.06%)
**length of pseudo-haplotype**	97 bp	116 bp	115 bp

Supplementary Table 4: Gene annotations for regions in the pseudo-haplotype obtained across different references in the NCBI data base. Melan is short for reference genome of *Wolbachia* in *Drosophila melanogaster*, similarly Aalbo for *Aedes albopictus* and Lectu for *Cimex lectularius* (see main text for details).

**start**	**end**	**ref**	**gene**	**product**	**protein_id**
1	6	Melan	ribA	GTP cyclohydrolase II	AAS13771.1
7	10	Melan	acnA	aconitate hydratase	AAS13860.1
11	11	Melan	NA	pyruvate dehydrogenase complex, E1 component, pyruvate dehydroge nase beta subunit, putative	AAS14193.1
12	19	Melan	NA	frame shift, not sequencing error	NA
20	26	Melan	NA	glycosyl transferase, group 1 family protein moaA/nifB/pqqE fami ly protein	AAS14314.1
27	33	Melan	gltX	glutamyl-tRNA synthetase	AAS14466.1
34	39	Melan	proS	prolyl-tRNA synthetase	AAS14500.1
40	46	Melan	NA	phosphoribosylformylglycinamidine synthase II domain protein	AAS14587.1
47	52	Melan	uvrD	helicase II - UvrD/PcrA	AAS14625.1
53	56	Melan	thrS	threonyl-tRNA synthetase	AAS14640.1
57	59	Melan	NA	glutamate–cysteine ligase-related protein	AAS14834.1
60	63	Melan	NA	hypothetical protein	AAS14876.1
64	65	Melan	NA	ATP-dependent RNA helicase, DeaD/DeaH box family	AAS14882.1
66	67	Aalbo	NA	mannose-1-phosphate guanyltransferase (partial)	NA
68	71	Aalbo	NA	hypothetical protein	WP_006015763.1
72	74	Aalbo	NA	glycine–tRNA ligase subunit beta	WP_006015767.1

Supplementary Table 5: Numbers of infected host individuals detected for 107 *Lycaeides* butterfly collection localities using different thresholds: the column labeled "# Infected 1x" indicates the number of infected individuals detected using a threshold of a minimum of one sequence read, the column labeled "# Infected 5x" indicates the number of infected individuals detected using a threshold of a minimum of five sequence reads, and the column labeled "# Infected 20x" indicates the number of infected individuals detected using a threshold of a minimum of 20 sequence reads. All sequence reads were at least 80bp in length.

#	Locality	Nominal Species	n	# Infected 1x	# Infected 5x	# Infected 20x
1	Fish Lk	L. samuelis	20	20	20	20
2	Eau Claire	L. samuelis	22	22	21	19
3	Black River	L. samuelis	17	17	17	17
4	Fort McCoy	L. samuelis	23	23	23	23
5	Indiana Dunes	L. samuelis	21	8	1	0
6	Allegan	L. samuelis	30	7	0	0
7	Saratoga Spr.s	L. samuelis	27	6	0	0
8	Fall Cr	L. anna	20	20	20	19
9	Yuba Gap	L. anna	20	20	20	20
10	Castle Pk	L. anna	18	17	16	16
11	Donner Pass	L. anna	18	17	17	17
12	Marlette Lk	L. anna	19	19	19	18
13	Leek Spr.s	L. anna	20	20	20	20
14	Cottonwood	L. idas	25	25	25	25
15	White Mt.	L. idas	24	24	24	24
16	StrawB Mt.s	L. idas	20	20	20	20
17	Siyeh Cr	L. idas	20	20	20	20
18	Soldier Cr	L. idas	20	20	19	19
19	Tibbs Butte	L. idas	20	20	20	20
20	King's Hill	L. idas	18	18	18	18
21	Garnet Pk	L. idas	20	19	19	19
22	Shook Mtn	L. idas	28	28	28	28
23	Wolftone Rd	L. idas	4	4	4	4
24	Bunsen Pk	L. idas	20	20	19	19
25	Hayden V	L. idas	22	22	22	22
26	Animas RH	L. idas	13	13	13	13
27	Red Mt. P	L. idas	4	4	4	4
28	Tomboy Rd	L. idas	24	24	24	24
29	Nolan Rd	L. idas	8	8	8	7
30	Spruce Barley	L. idas	20	20	20	20
31	Tok	L. idas	14	14	14	13
32	Tolovana Cr	L. idas	9	9	9	8
33	Soda Mt.	L. ricei	20	20	19	19
34	Rainy Pass	L. ricei	20	20	20	20
35	Chinook Pass	L. ricei	25	25	25	25
36	Big Lk	L. ricei	20	20	20	20
37	Cave Lake	L. ricei	24	24	24	24
38	Marble Mts.	L. ricei	12	8	7	7
39	Shovel Cr	L. ricei	21	20	20	20
40	Beulah	L. melissa - East	10	10	10	9
41	Brandon	L. melissa - East	20	19	18	18
42	Silver Cr	L. melissa - East	6	6	6	6
43	Richfield	L. melissa - East	6	5	5	5
44	Victor	L. melissa - East	20	20	20	20
45	Cokeville	L. melissa - East	10	10	10	10
46	Montrose	L. melissa - East	20	20	20	20
47	De Beque	L. melissa - East	20	20	19	19
48	Cimarron	L. melissa - East	6	6	6	6
49	Goose Lk	L. melissa - East	20	20	20	20
50	Montague	L. melissa - East	19	19	19	19
51	Susanville	L. melissa - East	10	10	10	10

Supplementary Table 5 - *Continued from previous page*

#	Locality	Nominal Species	n	# Infected 1x	# Infected 5x	# Infected 20x
52	Abel Cr	L. melissa - East	19	19	19	19
53	Deeth	L. melissa - East	20	20	20	20
54	Mill Cr	L. melissa - East	24	24	24	24
55	East Cr CG	L. melissa - East	25	25	25	25
56	Lamoille	L. melissa - East	20	19	19	19
57	Ophir City	L. melissa - East	19	19	19	19
58	Star Cr	L. melissa - East	16	16	16	16
59	Upper Alkali	L. melissa - East	20	19	19	19
60	Surprise V	L. melissa - East	20	20	20	20
61	Cody	L. melissa - Rockies	23	23	22	22
62	Lander	L. melissa - Rockies	24	23	23	23
63	Wheatland	L. melissa - Rockies	16	16	16	16
64	Yellow Pine CG	L. melissa - Rockies	20	20	19	19
65	Albion Meadow	L. melissa - Rockies	46	46	46	46
66	Lake Davis	L. melissa - West	4	4	4	4
67	Sierravalley	L. melissa - West	20	20	20	20
68	White Lk	L. melissa - West	27	27	27	27
69	Silver Lk	L. melissa - West	18	17	17	17
70	Girl Farm	L. melissa - West	24	23	23	23
71	Verdi Crystal	L. melissa - West	73	70	68	68
72	Verdi Classic	L. melissa - West	26	26	25	25
73	Verdi Tracks	L. melissa - West	20	17	16	16
74	Verdi Hwy	L. melissa - West	11	11	11	10
75	Qui	L. melissa - West	18	17	16	16
76	Deer Mt Rd	L. melissa - West	27	25	23	23
77	Washoe Lk	L. melissa - West	20	19	18	18
78	Gardnerville	L. melissa - West	18	17	17	17
79	Red Earth	L. melissa - West	20	20	20	18
80	Bishop	L. melissa - West	20	20	20	20
81	Trout Pond	L. melissa - West	13	13	13	13
82	Big Ice	hybrid	18	18	18	18
83	Blacktail Butte	hybrid	46	45	45	45
84	Bull Cr	hybrid	46	46	45	45
85	Dubois	hybrid zone	41	41	41	41
86	Hunt Mt	hybrid	30	30	30	30
87	Periodic Spr	hybrid	20	20	20	20
88	Pinnacles Butte	hybrid	20	20	19	19
89	Rendezvous Mt	hybrid	32	32	32	32
90	Riddle Lk	hybrid	30	29	28	28
91	Sheffield Cr	hybrid	26	26	26	26
92	Swift Cr	hybrid	4	3	3	3
93	Buck Mt	hybrid	44	44	44	44
94	Eagle Pk	hybrid	40	40	40	40
95	Steens Mt	hybrid	13	13	11	11
96	Hinkley	hybrid?	26	26	26	26
97	Jarbidge	hybrid?	42	40	40	40
98	Mt Rose	hybrid	52	17	8	3
99	Carson Pass	hybrid	50	38	32	29
100	Corey Pk	hybrid	8	8	8	8
101	Sonora Pass	hybrid	44	34	28	25
102	Lake Emma	hybrid	33	26	17	12
103	Sweetwater	hybrid	23	16	13	12
104	Tioga Crest	hybrid	38	25	21	13
105	South Fork	hybrid	14	7	5	5
106	County Line	hybrid	40	36	35	35
107	Reed Flat	hybrid	9	9	8	8

Supplementary Table 6: Numbers of genotyped individuals for *Wolbachia* strains per year at the Verdi Crystal (71) locality.

**Year**	**strain *wLyc*A**	**strain *wLyc*B**	**strain *wLyc*H**
2011	1	0	0
2012	7	1	0
2017	6	4	1
2018	5	7	0

`BEAUti .xml` files

Listing 1: Settings for Net A

```
<run id="mcmc" spec="MCMC" chainLength="75000000" preBurnin="5000000" storeEvery="50000">
    <state id="state" spec="State" storeEvery="5000">
        <tree id="Tree.t:netA" spec="beast.evolution.tree.Tree" name="stateNode">
            <trait id="dateTrait.t:netA" spec="beast.evolution.tree.TraitSet" traitname="date" value="">
                <taxa id="TaxonSet.netA" spec="TaxonSet">
                    <alignment idref="netA"/>
                </taxa>
            </trait>
            <taxonset idref="TaxonSet.netA"/>
        </tree>
        <parameter id="clockRate.c:netA" spec="parameter.RealParameter" lower="0.0" name="stateNode" upper="2.0">1.0</parameter>
        <parameter id="kappa.s:netA" spec="parameter.RealParameter" lower="0.0" name="stateNode">2.0</parameter>
        <parameter id="bPopSizes.t:netA" spec="parameter.RealParameter" dimension="5" lower="0.0" name="stateNode">380.0</parameter>
        <stateNode id="bGroupSizes.t:netA" spec="parameter.IntegerParameter" dimension="5">1</stateNode>
    </state>

    <init id="RandomTree.t:netA" spec="beast.evolution.tree.RandomTree" estimate="false" initial="@Tree.t:netA" taxa="@netA">
        <populationModel id="ConstantPopulation0.t:netA" spec="ConstantPopulation">
            <parameter id="randomPopSize.t:netA" spec="parameter.RealParameter" name="popSize">1.0</parameter>
        </populationModel>
    </init>

    <distribution id="posterior" spec="util.CompoundDistribution">
        <distribution id="prior" spec="util.CompoundDistribution">
```

```
            <distribution id="BayesianSkyline.t:netA" spec="BayesianSkyline" groupSizes="
                 ↪ @bGroupSizes.t:netA" popSizes="@bPopSizes.t:netA">
                <treeIntervals id="BSPTreeIntervals.t:netA" spec="TreeIntervals" tree="@Tree.
                     ↪ t:netA"/>
            </distribution>
            <distribution id="MarkovChainedPopSizes.t:netA" spec="beast.math.distributions.
                 ↪ MarkovChainDistribution" jeffreys="true" parameter="@bPopSizes.t:netA"/>
            <prior id="ClockPrior.c:netA" name="distribution" x="@clockRate.c:netA">
                <Uniform id="Uniform.0" name="distr" upper="Infinity"/>
            </prior>
            <prior id="KappaPrior.s:netA" name="distribution" x="@kappa.s:netA">
                <LogNormal id="LogNormalDistributionModel.0" name="distr">
                    <parameter id="RealParameter.1" spec="parameter.RealParameter" estimate="
                         ↪ false" name="M">1.0</parameter>
                    <parameter id="RealParameter.2" spec="parameter.RealParameter" estimate="
                         ↪ false" name="S">1.25</parameter>
                </LogNormal>
            </prior>
        </distribution>
        <distribution id="likelihood" spec="util.CompoundDistribution" useThreads="true">
            <distribution id="treeLikelihood.netA" spec="ThreadedTreeLikelihood" data="@netA
                 ↪ " tree="@Tree.t:netA">
                <siteModel id="SiteModel.s:netA" spec="SiteModel">
                    <parameter id="mutationRate.s:netA" spec="parameter.RealParameter"
                         ↪ estimate="false" name="mutationRate">1.0</parameter>
                    <parameter id="gammaShape.s:netA" spec="parameter.RealParameter" estimate
                         ↪ ="false" name="shape">1.0</parameter>
                    <parameter id="proportionInvariant.s:netA" spec="parameter.RealParameter"
                         ↪  estimate="false" lower="0.0" name="proportionInvariant" upper="
                         ↪ 1.0">0.0</parameter>
                    <substModel id="hky.s:netA" spec="HKY" kappa="@kappa.s:netA">
                        <frequencies id="empiricalFreqs.s:netA" spec="Frequencies" data="@netA
                             ↪ "/>
                    </substModel>
                </siteModel>
```

```
                <branchRateModel id="StrictClock.c:netA" spec="beast.evolution.
                    ↪ branchratemodel.StrictClockModel" clock.rate="@clockRate.c:netA"/>
            </distribution>
        </distribution>
    </distribution>

    <operator id="StrictClockRateScaler.c:netA" spec="ScaleOperator" parameter="@clockRate.
        ↪ c:netA" scaleFactor="0.75" weight="3.0"/>

    <operator id="strictClockUpDownOperator.c:netA" spec="UpDownOperator" scaleFactor="0.75"
        ↪  weight="3.0">
        <up idref="clockRate.c:netA"/>
        <down idref="Tree.t:netA"/>
    </operator>

    <operator id="KappaScaler.s:netA" spec="ScaleOperator" parameter="@kappa.s:netA"
        ↪ scaleFactor="0.5" weight="0.1"/>

    <operator id="BayesianSkylineTreeScaler.t:netA" spec="ScaleOperator" scaleFactor="0.5"
        ↪ tree="@Tree.t:netA" weight="3.0"/>

    <operator id="BayesianSkylineTreeRootScaler.t:netA" spec="ScaleOperator" rootOnly="true"
        ↪  scaleFactor="0.5" tree="@Tree.t:netA" weight="3.0"/>

    <operator id="BayesianSkylineUniformOperator.t:netA" spec="Uniform" tree="@Tree.t:netA"
        ↪ weight="30.0"/>

    <operator id="BayesianSkylineSubtreeSlide.t:netA" spec="SubtreeSlide" tree="@Tree.t:netA
        ↪ " weight="15.0"/>

    <operator id="BayesianSkylineNarrow.t:netA" spec="Exchange" tree="@Tree.t:netA" weight="
        ↪ 15.0"/>

    <operator id="BayesianSkylineWide.t:netA" spec="Exchange" isNarrow="false" tree="@Tree.
        ↪ t:netA" weight="3.0"/>
```

```

<operator id="BayesianSkylineWilsonBalding.t:netA" spec="WilsonBalding" tree="@Tree.
    ↪ t:netA" weight="3.0"/>

<operator id="popSizesScaler.t:netA" spec="ScaleOperator" parameter="@bPopSizes.t:netA"
    ↪ scaleFactor="0.75" weight="15.0"/>

<operator id="groupSizesDelta.t:netA" spec="DeltaExchangeOperator" integer="true" weight
    ↪ ="6.0">
    <intparameter idref="bGroupSizes.t:netA"/>
</operator>

<logger id="tracelog" spec="Logger" fileName="netA.log" logEvery="20000" model="
    ↪ @posterior" sanitiseHeaders="true" sort="smart">
    <log idref="posterior"/>
    <log idref="likelihood"/>
    <log idref="prior"/>
    <log idref="treeLikelihood.netA"/>
    <log id="TreeHeight.t:netA" spec="beast.evolution.tree.TreeHeightLogger" tree="@Tree
        ↪ .t:netA"/>
    <log idref="clockRate.c:netA"/>
    <log idref="kappa.s:netA"/>
    <log idref="BayesianSkyline.t:netA"/>
    <log idref="bPopSizes.t:netA"/>
    <log idref="bGroupSizes.t:netA"/>
</logger>

<logger id="screenlog" spec="Logger" logEvery="50000">
    <log idref="posterior"/>
    <log idref="likelihood"/>
    <log idref="prior"/>
</logger>

<logger id="treelog.t:netA" spec="Logger" fileName="$(tree).trees" logEvery="20000" mode
    ↪ ="tree">
```

```
        <log id="TreeWithMetaDataLogger.t:netA" spec="beast.evolution.tree.
            ↪ TreeWithMetaDataLogger" tree="@Tree.t:netA"/>
    </logger>

    <operatorschedule id="OperatorSchedule" spec="OperatorSchedule"/>

</run>
```

Listing 2: Settings for Net B (same settings used for Net C)

```
<run id="mcmc" spec="MCMC" chainLength="50000000" preBurnin="1000000" storeEvery="50000">
    <state id="state" spec="State" storeEvery="5000">
        <tree id="Tree.t:netB" spec="beast.evolution.tree.Tree" name="stateNode">
            <trait id="dateTrait.t:netB" spec="beast.evolution.tree.TraitSet" traitname="
                ↪ date" value="">
                <taxa id="TaxonSet.netB" spec="TaxonSet">
                    <alignment idref="netB"/>
                </taxa>
            </trait>
            <taxonset idref="TaxonSet.netB"/>
        </tree>
        <parameter id="bPopSizes.t:netB" spec="parameter.RealParameter" dimension="5" lower=
            ↪ "0.0" name="stateNode">380.0</parameter>
        <stateNode id="bGroupSizes.t:netB" spec="parameter.IntegerParameter" dimension="5">1
            ↪ </stateNode>
        <parameter id="clockRate.c:netB" spec="parameter.RealParameter" lower="0.0" name="
            ↪ stateNode" upper="2.0">1.0</parameter>
        <parameter id="kappa.s:netB" spec="parameter.RealParameter" lower="0.0" name="
            ↪ stateNode">2.0</parameter>
    </state>

    <init id="RandomTree.t:netB" spec="beast.evolution.tree.RandomTree" estimate="false"
        ↪ initial="@Tree.t:netB" taxa="@netB">
        <populationModel id="ConstantPopulation0.t:netB" spec="ConstantPopulation">
            <parameter id="randomPopSize.t:netB" spec="parameter.RealParameter" name="
```

```
                ↪ popSize">1.0</parameter>
        </populationModel>
    </init>

    <distribution id="posterior" spec="util.CompoundDistribution">
        <distribution id="prior" spec="util.CompoundDistribution">
            <distribution id="BayesianSkyline.t:netB" spec="BayesianSkyline" groupSizes="
                ↪ @bGroupSizes.t:netB" popSizes="@bPopSizes.t:netB">
                <treeIntervals id="BSPTreeIntervals.t:netB" spec="TreeIntervals" tree="@Tree.
                    ↪ t:netB"/>
            </distribution>
            <distribution id="MarkovChainedPopSizes.t:netB" spec="beast.math.distributions.
                ↪ MarkovChainDistribution" jeffreys="true" parameter="@bPopSizes.t:netB"/>
            <prior id="ClockPrior.c:netB" name="distribution" x="@clockRate.c:netB">
                <Uniform id="Uniform.6" name="distr" upper="Infinity"/>
            </prior>
            <prior id="KappaPrior.s:netB" name="distribution" x="@kappa.s:netB">
                <LogNormal id="LogNormalDistributionModel.0" name="distr">
                    <parameter id="RealParameter.1" spec="parameter.RealParameter" estimate="
                        ↪ false" name="M">1.0</parameter>
                    <parameter id="RealParameter.2" spec="parameter.RealParameter" estimate="
                        ↪ false" name="S">1.25</parameter>
                </LogNormal>
            </prior>
        </distribution>
        <distribution id="likelihood" spec="util.CompoundDistribution" useThreads="true">
            <distribution id="treeLikelihood.netB" spec="ThreadedTreeLikelihood" data="@netB
                ↪ " tree="@Tree.t:netB">
                <siteModel id="SiteModel.s:netB" spec="SiteModel">
                    <parameter id="mutationRate.s:netB" spec="parameter.RealParameter"
                        ↪ estimate="false" name="mutationRate">1.0</parameter>
                    <parameter id="gammaShape.s:netB" spec="parameter.RealParameter" estimate
                        ↪ ="false" name="shape">1.0</parameter>
                    <parameter id="proportionInvariant.s:netB" spec="parameter.RealParameter"
                        ↪  estimate="false" lower="0.0" name="proportionInvariant" upper="
```

```
                        ↪ 1.0">0.0</parameter>
                <substModel id="hky.s:netB" spec="HKY" kappa="@kappa.s:netB">
                    <frequencies id="empiricalFreqs.s:netB" spec="Frequencies" data="@netB
                         ↪ "/>
                </substModel>
            </siteModel>
            <branchRateModel id="StrictClock.c:netB" spec="beast.evolution.
                 ↪ branchratemodel.StrictClockModel" clock.rate="@clockRate.c:netB"/>
        </distribution>
    </distribution>
</distribution>

<operator id="BayesianSkylineTreeScaler.t:netB" spec="ScaleOperator" scaleFactor="0.5"
     ↪ tree="@Tree.t:netB" weight="3.0"/>

<operator id="BayesianSkylineTreeRootScaler.t:netB" spec="ScaleOperator" rootOnly="true"
     ↪  scaleFactor="0.5" tree="@Tree.t:netB" weight="3.0"/>

<operator id="BayesianSkylineUniformOperator.t:netB" spec="Uniform" tree="@Tree.t:netB"
     ↪ weight="30.0"/>

<operator id="BayesianSkylineSubtreeSlide.t:netB" spec="SubtreeSlide" tree="@Tree.t:netB
     ↪ " weight="15.0"/>

<operator id="BayesianSkylineNarrow.t:netB" spec="Exchange" tree="@Tree.t:netB" weight="
     ↪ 15.0"/>

<operator id="BayesianSkylineWide.t:netB" spec="Exchange" isNarrow="false" tree="@Tree.
     ↪ t:netB" weight="3.0"/>

<operator id="BayesianSkylineWilsonBalding.t:netB" spec="WilsonBalding" tree="@Tree.
     ↪ t:netB" weight="3.0"/>

<operator id="popSizesScaler.t:netB" spec="ScaleOperator" parameter="@bPopSizes.t:netB"
     ↪ scaleFactor="0.75" weight="15.0"/>
```

```
    <operator id="groupSizesDelta.t:netB" spec="DeltaExchangeOperator" integer="true" weight
        ↪ ="6.0">
        <intparameter idref="bGroupSizes.t:netB"/>
    </operator>

    <operator id="StrictClockRateScaler.c:netB" spec="ScaleOperator" parameter="@clockRate.
        ↪ c:netB" scaleFactor="0.75" weight="3.0"/>

    <operator id="strictClockUpDownOperator.c:netB" spec="UpDownOperator" scaleFactor="0.75"
        ↪  weight="3.0">
        <up idref="clockRate.c:netB"/>
        <down idref="Tree.t:netB"/>
    </operator>

    <operator id="KappaScaler.s:netB" spec="ScaleOperator" parameter="@kappa.s:netB"
        ↪ scaleFactor="0.5" weight="0.1"/>

    <logger id="tracelog" spec="Logger" fileName="netB.log" logEvery="10000" model="
        ↪ @posterior" sanitiseHeaders="true" sort="smart">
        <log idref="posterior"/>
        <log idref="likelihood"/>
        <log idref="prior"/>
        <log idref="treeLikelihood.netB"/>
        <log id="TreeHeight.t:netB" spec="beast.evolution.tree.TreeHeightLogger" tree="@Tree
            ↪ .t:netB"/>
        <log idref="BayesianSkyline.t:netB"/>
        <log idref="bPopSizes.t:netB"/>
        <log idref="bGroupSizes.t:netB"/>
        <log idref="clockRate.c:netB"/>
        <log idref="kappa.s:netB"/>
    </logger>

    <logger id="screenlog" spec="Logger" logEvery="50000">
        <log idref="posterior"/>
```

```
        <log idref="likelihood"/>
        <log idref="prior"/>
    </logger>

    <logger id="treelog.t:netB" spec="Logger" fileName="$(tree).trees" logEvery="10000" mode="tree">
        <log id="TreeWithMetaDataLogger.t:netB" spec="beast.evolution.tree.TreeWithMetaDataLogger" tree="@Tree.t:netB"/>
    </logger>

    <operatorschedule id="OperatorSchedule" spec="OperatorSchedule"/>

</run>
```

References

Chaturvedi S, Lucas LK, Nice CC, Fordyce JA, Forister ML, Gompert Z (2018) The predictability of genomic changes underlying a recent host shift in Melissa blue butterflies. *Molecular Ecology*, **27**, 2651–2666.

Gompert Z, Lucas LK, Buerkle CA, Forister ML, Fordyce JA, Nice CC (2014) Admixture and the organization of genetic diversity in a butterfly species complex revealed through common and rare genetic variants. *Molecular Ecology*, **23**, 4555–4573.
